# Histological Transformation and Progression in Follicular Lymphoma: A Clonal Evolution Study

**DOI:** 10.1371/journal.pmed.1002197

**Published:** 2016-12-13

**Authors:** Robert Kridel, Fong Chun Chan, Anja Mottok, Merrill Boyle, Pedro Farinha, King Tan, Barbara Meissner, Ali Bashashati, Andrew McPherson, Andrew Roth, Karey Shumansky, Damian Yap, Susana Ben-Neriah, Jamie Rosner, Maia A. Smith, Cydney Nielsen, Eva Giné, Adele Telenius, Daisuke Ennishi, Andrew Mungall, Richard Moore, Ryan D. Morin, Nathalie A. Johnson, Laurie H. Sehn, Thomas Tousseyn, Ahmet Dogan, Joseph M. Connors, David W. Scott, Christian Steidl, Marco A. Marra, Randy D. Gascoyne, Sohrab P. Shah

**Affiliations:** 1 Centre for Lymphoid Cancer, BC Cancer Agency, Vancouver, British Columbia, Canada; 2 Bioinformatics Graduate Program, University of British Columbia, Vancouver, British Columbia, Canada; 3 Department of Pathology and Laboratory Medicine, University of British Columbia, Vancouver, British Columbia, Canada; 4 Department of Molecular Oncology, BC Cancer Agency, Vancouver, British Columbia, Canada; 5 Michael Smith Genome Sciences Centre, BC Cancer Agency, Vancouver, British Columbia, Canada; 6 Department of Molecular Biology and Biochemistry, Simon Fraser University, Burnaby, British Columbia, Canada; 7 Jewish General Hospital, Montreal, Quebec, Canada; 8 Translational Cell and Tissue Research Lab, Department for Imaging and Pathology, University of Leuven (KU Leuven), Leuven, Belgium; 9 Department of Pathology, Universitaire Ziekenhuizen Leuven (UZ Leuven), Leuven, Belgium; 10 Department of Laboratory and Pathology, Mayo Clinic, Rochester, Minnesota, United States of America; 11 Departments of Pathology and Laboratory Medicine, Memorial Sloan Kettering Cancer Center, New York, New York, United States of America; Washington University School of Medicine, UNITED STATES

## Abstract

**Background:**

Follicular lymphoma (FL) is an indolent, yet incurable B cell malignancy. A subset of patients experience an increased mortality rate driven by two distinct clinical end points: histological transformation and early progression after immunochemotherapy. The nature of tumor clonal dynamics leading to these clinical end points is poorly understood, and previously determined genetic alterations do not explain the majority of transformed cases or accurately predict early progressive disease. We contend that detailed knowledge of the expansion patterns of specific cell populations plus their associated mutations would provide insight into therapeutic strategies and disease biology over the time course of FL clinical histories.

**Methods and Findings:**

Using a combination of whole genome sequencing, targeted deep sequencing, and digital droplet PCR on matched diagnostic and relapse specimens, we deciphered the constituent clonal populations in 15 transformation cases and 6 progression cases, and measured the change in clonal population abundance over time. We observed widely divergent patterns of clonal dynamics in transformed cases relative to progressed cases. Transformation specimens were generally composed of clones that were rare or absent in diagnostic specimens, consistent with dramatic clonal expansions that came to dominate the transformation specimens. This pattern was independent of time to transformation and treatment modality. By contrast, early progression specimens were composed of clones that were already present in the diagnostic specimens and exhibited only moderate clonal dynamics, even in the presence of immunochemotherapy. Analysis of somatic mutations impacting 94 genes was undertaken in an extension cohort consisting of 395 samples from 277 patients in order to decipher disrupted biology in the two clinical end points. We found 12 genes that were more commonly mutated in transformed samples than in the preceding FL tumors, including *TP53*, *B2M*, *CCND3*, *GNA13*, *S1PR2*, and *P2RY8*. Moreover, ten genes were more commonly mutated in diagnostic specimens of patients with early progression, including *TP53*, *BTG1*, *MKI67*, and *XBP1*.

**Conclusions:**

Our results illuminate contrasting modes of evolution shaping the clinical histories of transformation and progression. They have implications for interpretation of evolutionary dynamics in the context of treatment-induced selective pressures, and indicate that transformation and progression will require different clinical management strategies.

## Abstract 2

Sohrab Shah and colleagues explore the evolutionary histories that shape clinical and transformation dynamics in follicular lymphoma

## Author Summary

**Why Was This Study Done?:**

- Follicular lymphoma (FL) is a largely incurable malignancy in which early progression and transformation have consistently been linked to lymphoma-related mortality.
- We contended that detailed characterization of clonal dynamics would reveal fundamental biological properties with implications for future patient management strategies relating to both transformation and progression.
- We also sought to identify recurrent gene mutations associated with transformation and/or early progression in a large patient cohort.

**What Did the Researchers Do and Find?:**

- Using whole genome sequencing, deep allelic sampling by amplicon sequencing, and digital droplet PCR, we found dramatic clonal expansions in transformed disease, whereby dominant clones in transformation samples emerged from extremely low prevalence clones or from clones that were not detected in the diagnostic samples.
- The dynamics of disease progression during treatment in the absence of transformation showed markedly different characteristics, with much of the clonal architecture preserved from diagnostic to relapse specimens.
- Targeted capture-based sequencing in a large extension cohort then established genetic variants associated with transformation and early progression in the broader patient population.

**What Do These Findings Mean?:**

- Taken together, our findings illuminate previously undescribed patterns of clonal expansion underpinning FL clinical histories suggesting that contrasting management strategies will be necessary across the FL patient population.
- We uncovered novel associations of gene mutations with early progression that could inform future prognostic assay development.

## Introduction

Follicular lymphoma (FL) is the second most common subtype of non-Hodgkin lymphoma and the most frequent indolent lymphoma, accounting for 22%–32% of all new non-Hodgkin lymphoma diagnoses in Western countries [1,2]. Patient outcomes are favorable, with median overall survival extending well beyond 10 y [3–5]. However, FL remains an incurable malignancy as most patients eventually experience progressive disease. A subset of patients are at risk of early lymphoma-related mortality due to early progression after immunochemotherapy or to histological transformation to aggressive lymphoma (2%–3% of patients per year), both of which lead to shortened survival [6–13]. Hence, mutational profiling of FL specimens at the temporal boundaries of clinical inflection points represents a compelling opportunity to study the evolutionary dynamics underpinning FL disease progression.

To infer evolutionary properties, deconvolution of malignant tissues into constituent clones is required. Clonal decomposition is accomplished through analysis of allelic measurements, under the assumption that the prevalence of specific alleles in a DNA mixture extracted from a tumor quantitatively represents clonal population abundance. Here, we brought to bear targeted amplicon sequencing plus ultra-sensitive digital droplet PCR in order to measure the changing prevalence of alleles at unprecedented resolution over FL disease progression. With precise measurements of alleles, computational inference can then determine clonal composition and the phylogenetic topology of clones, yielding insight into temporal mutation acquisition and genotypes giving rise to clonal expansions over time. With this approach, longitudinal comparison of the clonal composition of tumors sampled at different time points in patient’s clinical history can be performed, deciphering which constituent populations were present at diagnosis, and which populations constituted the relapse. Thus, the degree to which a tumor is evolving and the contributions of specific clones to the evolutionary process (collectively termed clonal dynamics) can be quantitatively assessed.

To varying levels of resolution, related approaches have been applied to a variety of progression scenarios in hematologic and solid malignancies [14–17]. For example, secondary acute myeloid leukemia from underlying myelodysplastic syndrome and Richter syndrome from chronic lymphocytic leukemia arise without significant branched evolution [18,19]. By contrast, transformation of FL has most commonly been described as divergent branched evolution from a common progenitor [20,21]. The nature of clonal trajectories leading to transformation or early progression are poorly understood; it is unknown if similar, or contrasting, modes of selection underpin these clinical end points.

Discrete transformation-associated genetic alterations have been described involving *CDKN2A*, *MYC*, *TP53*, *CD58*, or *B2M* [20–29]. However, these events alone cannot explain the majority of transformed cases, leaving a discovery gap for genetic drivers of transformation. Similarly, progression has been described to occur more frequently in the presence of selected, recurrent cytogenetic aberrations or single gene mutations [30–36]. Recently, a clinicogenetic risk model (m7-FLIPI), including the mutational status of seven genes, the Follicular Lymphoma International Prognostic Index (FLIPI), and performance status, was shown to improve outcome prediction for patients requiring immunochemotherapy [37]. Nonetheless, the m7-FLIPI imperfectly captures determinants of early progression [13]. A newer prognostic model, named POD24-PI, was developed using the original m7-FLIPI data to specifically predict early progression. The POD24-PI has better sensitivity but lower specificity for the prediction of early progression [13], raising the question of whether progressive disease might be attributed to genetic lesions that are not captured by either prognostic model. Furthermore, the mechanisms underlying resistance to immunochemotherapy remain elusive; genetic profiling of early progression cases has the potential to uncover novel genetic lesions in molecular pathways leading to treatment resistance.

To address these questions, we set out to compare the clonal dynamics of tumors leading to transformation and those associated with early progression. We executed in-depth, high-resolution genome-wide profiling of mutant alleles. In addition, we aimed to establish the patient population prevalence of genetic events associated with transformation and early progression through targeted sequencing of a large cohort of samples with accompanying clinical outcome data.

## Methods

### Patients and Materials

Patient specimens were collected as part of research projects approved by the research ethics boards of the University of British Columbia–British Columbia Cancer Agency (H13-01765), UZ Leuven (S-55498), or the Mayo Clinic (08–005005).

**Fig 1:**
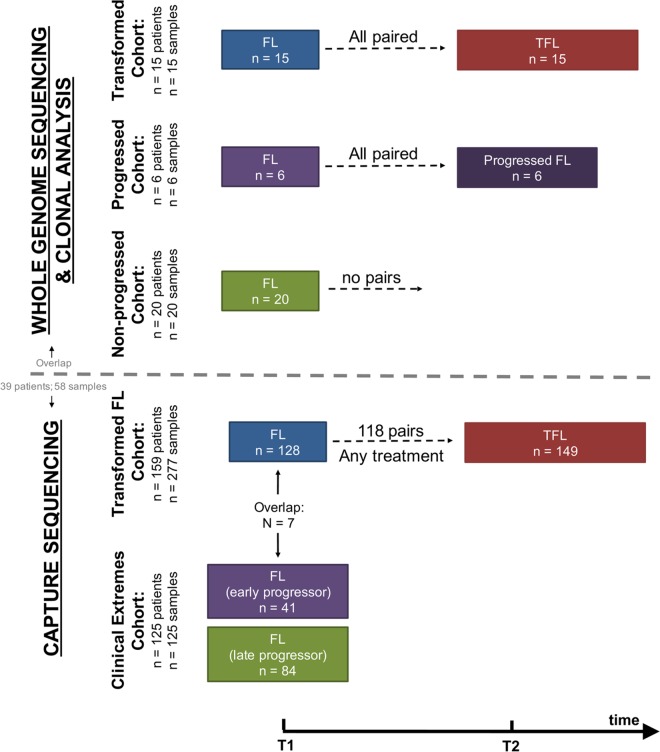
Study cohort overview. Whole genome sequencing (top) and capture sequencing (extension) (bottom) cohorts, as well as the repartition of patients and samples into clinical groups. FL, follicular lymphoma; TFL, transformed follicular lymphoma.

We assembled a cohort of tumor and normal specimens from 41 patients selected for whole genome sequencing (WGS) (Figs 1 and S1). Samples were acquired from the BC Cancer Agency lymphoma tumor bank, and patients were grouped according to three clinical end points: patients who presented with transformation (transformed FL [TFL], *n* = 15), those whose disease progressed without evidence of transformation (progressed FL [PFL], *n* = 6), and those whose lymphoma displayed no evidence of transformation or progression for more than 5 y after initial diagnosis (non-progressed FL [NPFL], *n* = 20). Paired tumor samples from fresh frozen blocks or cell suspensions consisting of diagnostic and relapse specimens for TFL and PFL patients and single diagnostic specimens for NPFL patients were acquired. We refer to samples from the primary time point as T1 samples, and those from the time of transformation (TFL cases) or progression (PFL cases) as T2 samples. Tumor and normal specimens comprising 103 WGS libraries in total were sequenced, yielding 62 tumor samples sequenced to an average 62.7-fold ± 23.0-fold coverage, and matching germline DNA sequenced to an average 35.2-fold ± 12.8-fold coverage (S2 Fig; S1 Table). Somatic single nucleotide variants (sSNVs), somatic small insertions and deletions (sIndels), somatic copy number alterations (sCNAs), and structural rearrangements were predicted for each tumor sample as described in S1 Supporting Appendix.

We next constructed a larger extension cohort consisting of samples from 277 patients used for targeted, capture-based sequencing (Fig 1). Patients were grouped into three categories: patients presenting with transformation (*n* = 159) and patients presenting with either early disease progression (*n* = 41) or late/never progression (*n* = 84). Early progression was defined as progression occurring within 2.5 y after starting treatment, which was intended to consist of rituximab chemotherapy followed by rituximab maintenance. Late/never progression was defined as no progression of lymphoma for at least 5 y after initiation of either observation or rituximab chemotherapy and rituximab maintenance. The majority of samples (96%) were acquired from the BC Cancer Agency lymphoma tumor bank, and a smaller number from the Mayo Clinic (2%) and the University of Leuven (2%). DNA from the extension cohort was subjected to a hybrid-capture-based panel of 94 genes and was sequenced to 1046.96-fold ± 229.88-fold coverage for fresh frozen samples and to 192.56-fold ± 120.49-fold coverage for formalin-fixed paraffin-embedded tissue samples.

Complete information on patient cohorts and sample preparation can be found in S2 Table and in S1 Supporting Appendix. All sequencing data are available for download through the European Genome-phenome Archive under accession number EGAS00001001709.

### Data Analysis

Detailed bioinformatics methods are presented in S1 Supporting Appendix. Briefly, WGS data were processed to provide sSNV, sIndel, sCNA, and structural rearrangement predictions. For inference of clonal structure, we selected ≥192 sSNVs or sIndels per patient and performed targeted deep amplicon sequencing, providing precise allelic measurements. A subset of mutations were profiled using digital droplet PCR. Those data, together with copy number status and tumor content, were used as an input for inference of clonal dynamics using previously described computational techniques [38,39].

In the extension cohort, we sequenced, using capture-based sequencing, the coding sequence of 86 genes as well as the 5′ regions of 20 genes that are targets of somatic hypermutation (12 genes overlapping with the 86 previously mentioned genes, i.e., 94 genes in total). sSNVs and sIndels were called as described in S1 Supporting Appendix. The proportions of samples harboring somatic mutations were compared between clinical groups using Bayesian proportion tests.

## Results

### Transformed/Progressed Follicular Lymphoma Samples Exhibit Higher Mutational Burden than Diagnostic Samples

**Fig 2:**
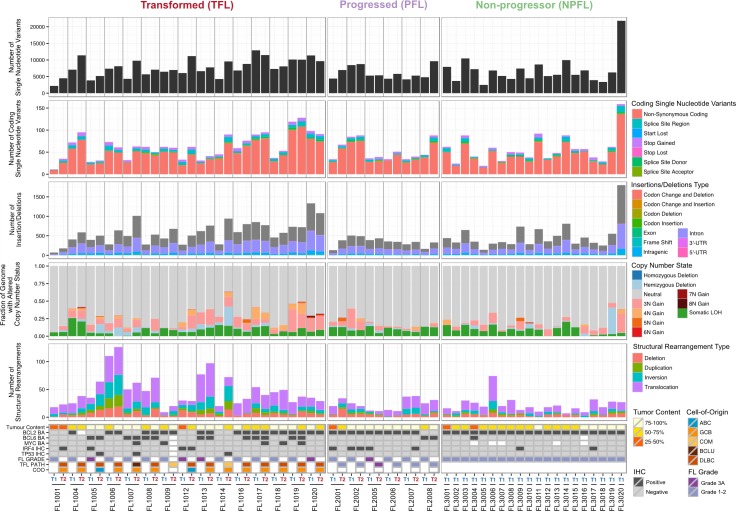
High-level WGS analysis overview. Number of genomic alterations by sample and by clinical group. For the TFL and PFL patients, the T1 and T2 sample are placed beside each other. Each panel represents a different mutation type (sSNV, sIndel, sCNA, structural rearrangement), with the number of mutations on the *y*-axes. Different colors represent the different categories of mutations within each mutation type. For sCNAs, the fraction of genome altered is plotted and copy number states are mutually exclusive. The somatic LOH class encapsulates all LOH events regardless of their absolute sCNA state (3N, 4N, etc.). Refer to S3 Table for more details on copy number state annotations. ABC, activated B-cell-like; BCLU, B cell lymphoma unclassified; COM, composite lymphoma; DLBC, diffuse large B cell lymphoma; FL, follicular lymphoma; GCB, germinal center B-cell-like; IHC, immunohistochemistry; LOH, loss of heterozygosity; NPFL, non-progressed follicular lymphoma; PFL, progressed follicular lymphoma; sCNA, somatic copy number alteration; sIndel, somatic small insertion or deletion; sSNV, somatic single nucleotide variant; TFL, transformed follicular lymphoma.

**Fig 3:**
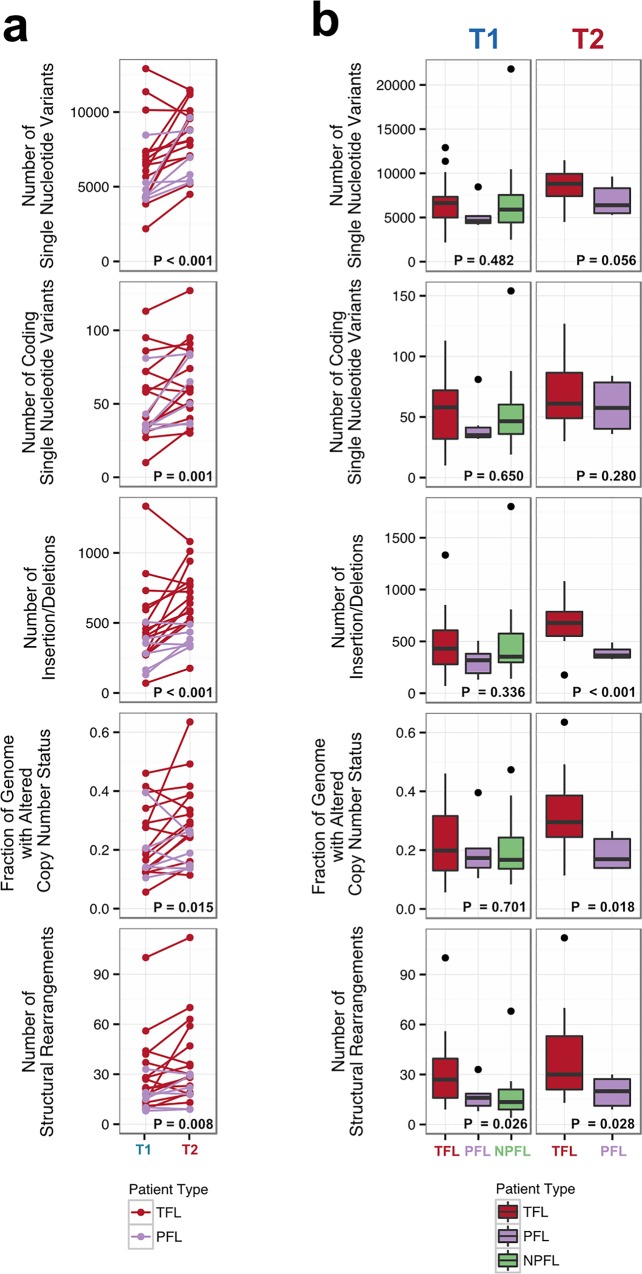
Comparative analysis between clinical groups. (A) Genomic alteration load in T1 versus T2 samples (for TFL and PFL patients). A one-tailed Wilcoxon test was used to assess whether there was a higher mutational burden in T2 samples compared to T1 samples. (B) Number of genetic alterations by time point and by clinical group. A Kruskal-Wallis test was used to assess whether there were differences in mutational burden between the T1 samples of the TFL, PFL, and NPFL clinical groups. A one-tailed Wilcoxon test was used to assess whether there were differences in mutational burden between the T2 samples of the TFL and PFL clinical groups. NPFL, non-progressed follicular lymphoma; PFL, progressed follicular lymphoma; TFL, transformed follicular lymphoma.

We began our analysis by comparing mutational burden over time in T1 and T2 samples from the WGS cohort. At T1, the average number of alterations was 7,133.29 ± 3,107.02 (range 2,184–21,802) for sSNVs, 512.63 ± 296.67 (range 70–1,801) for sIndels, and 26.24 ± 21.28 (range 4–112) for structural rearrangements across all WGS tumor samples (Fig 2). The mutational burden was significantly higher in T2 than in T1 samples for all mutation types (in both TFL and PFL patients) (Fig 3A) and was independent of the time interval between sampling (S3 Fig).

When comparing the three clinical groups (TFL, PFL, and NPFL), baseline mutation rates at T1 did not differ for sSNVs, sIndels, and sCNAs (T1 facet of Fig 3B), suggesting that the increase in mutation rate for TFL and PFL cases was acquired after diagnosis. However, the number of structural rearrangements was higher in TFL T1 samples (31.33 ± 23.29) than in PFL T1 samples (17.00 ± 8.88) and NPFL samples (16.90 ± 13.76) (Kruskal-Wallis *p* = 0.026; T1 facet of Fig 3B), consistent with TFL cases at diagnosis harboring an increased propensity to accumulate translocations. Comparison of TFL and PFL T2 samples revealed a higher number of sIndels (one-tailed Wilcox *p <* 0.001), a higher proportion of the genome altered by sCNAs (one-tailed Wilcox *p* = 0.018), and a higher number of rearrangements in TFL samples compared to PFL samples (one-tailed Wilcox *p* = 0.028; T2 facet of Fig 3B), suggesting that histological transformation is associated with a higher mutational rate in the structural genome relative to samples that progressed on therapy. Overall, a higher mutational burden in T2 samples relative to T1 samples was observed, with a more pronounced effect in TFL cases.

### Histological Transformation Emerges from Expansion of Clones That Are Rare in Diagnostic Samples

**Fig 4:**
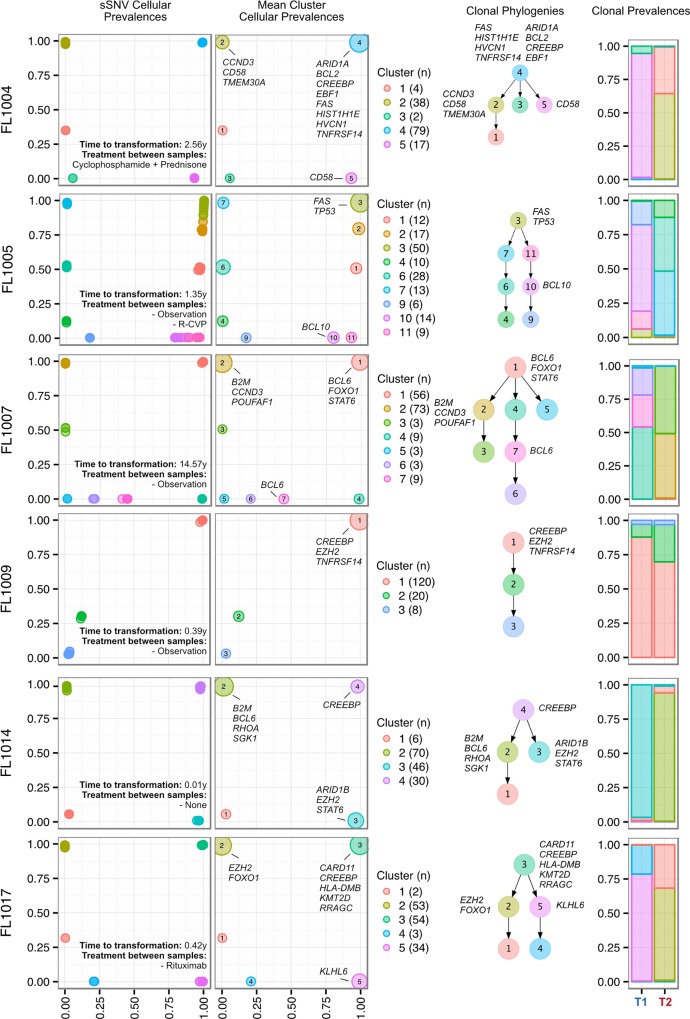
Clonal phylogenies of transformed follicular lymphoma samples. From mutation cellular prevalences to clonal phylogenies and clonal prevalences for TFL patients. For each given patient, the leftmost plot shows the PyClone cellular prevalence of each validated sSNV (i.e., somatic in the T1 and/or T2 sample) at T1 (*x*-axes) and T2 (*y*-axes), with each mutation colored by the cluster it belongs to. The next plot to the right represents the cluster cellular prevalence (mean cellular prevalence of all mutations in the cluster), with the size of the circle representing the number of mutations in the cluster. This is followed by a clonal phylogeny and then a stacked bar plot representing the clonal prevalence of each clone in the T1 and T2 sample. The colors of the clusters have no meaning across patients. The *n* in parentheses beside the cluster color and number represents the number of sSNVs in that cluster. R-CVP, cyclophosphamide, vincristine, and prednisone plus rituximab; sSNV, somatic single nucleotide variant; TFL, transformed follicular lymphoma.

**Fig 5:**
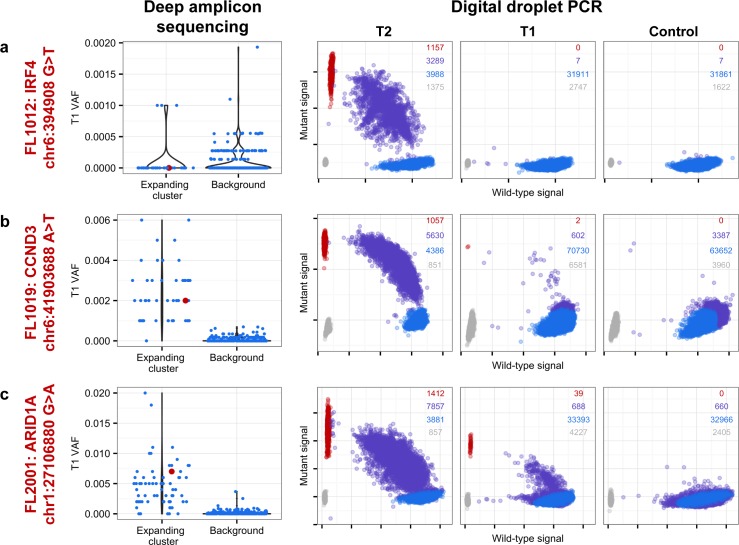
Ultra-sensitive detection of low prevalence clones in T1 samples. Shown are three mutations (A–C) in three patients (FL1012, FL1019, and FL2001) in which PyClone suggested that the expanded T2-dominant mutation clusters were present at near zero prevalence at T1. No evidence of read support, when compared to background, was found for the T2-associated mutation in the T1 sample for case FL1012, in contrast to cases FL1019 and FL2001 (leftmost plots). Background refers to variant allele frequencies of all possible single nucleotide changes in the vicinity of the mutation of interest (defined as up to 50 base pairs upstream and up to 50 base pairs downstream). The results are confirmed by digital droplet PCR (rightmost plots). Color coding in the digital droplet PCR plots is as follows: grey = empty droplets; blue = single-positive droplets for wild-type allele; purple = double-positive droplets; red = single-positive droplets for mutant allele.

We next profiled the clonal composition and evolutionary changes of T1 and T2 samples. All T1–T2 pairs exhibited uniclonal origin by virtue of shared mutations comprising an ancestral clone in addition to a substantial fraction of T1-specific (0.175 ± 0.105 [minimum–maximum, 0.038–0.431]) and T2-specific mutations (0.366 ± 0.166 [0.063–0.664]) (S4 Fig, contour density on T1 and T2 axes). However, comparative analysis of the clonal structure of T1 and T2 samples (S1 Supporting Appendix; S4 Table) revealed dramatic clonal dynamics in 13 of 15 TFL patients (87%). In these 13 patients, T2 samples were composed primarily of divergent clones (or phylogenetic lineages) that were extremely rare (<1%) in T1 samples (Figs 4 and S5). This defined a characteristic mode of evolution with massive expansion of clones in T2 samples that were rare or detectably absent in T1 samples. This suggests that diagnostic samples are not likely to possess reliable predictors of transformation in the majority of cases, and that the clonal dynamics occurring after diagnosis likely underpin histological change. This pattern was independent of time to transformation. For example, the T2 sample from FL1007 (transformed after 14.57 y; Fig 4), characterized by *FOXO1* and *BCL6* mutations in the ancestral clone (cluster 1), was entirely composed of a clonal lineage harboring *B2M* and *CCND3* mutations (clusters 2 and 3) that were near zero prevalence levels in the T1 sample. Notably, these clones were mutually exclusive to the clonal lineage dominating the T1 sample (clusters 4, 7, 6, and 5). The T2 sample from FL1017 (transformed after 0.42 y; Fig 4), characterized by *CREBBP* and *KMT2D* mutations in its ancestral clone, harbored a T2-specific lineage containing *EZH2* and *FOXO1* mutations (clusters 2 and 1), exhibiting a distribution of clones similar to that of FL1007. This pattern of clonal dynamics was independent of treatment regimen and was found in untreated cases (observation alone; FL1007, FL1006, FL1012, FL1014, and FL1019) and in cases treated with rituximab and/or chemotherapy (FL1001, FL1004, FL1005, FL1008, FL1013, FL1016, and FL1017). The pattern of expansion from undetectable or extremely rare clones (<1%) was validated using orthogonal digital droplet PCR technology (S1 Supporting Appendix) in 3/3 TFL cases attempted, confirming that a clone as rare as 2 out of approximately 10^5^ cells at diagnosis came to dominate the transformed specimen (Figs 5A, 5B and S6A–S6C). We also observed this signal in the extension cohort in 18 cases out of 32 (56%) that were available for analysis and not overlapping with our WGS cohort (S7 Fig). These observations were made from a sparse sampling of only 94 genes and yet still yielded similar patterns where at least one mutation exhibited increased prevalence from near zero in the T1 sample to dominant levels in the T2 sample.

Two TFL cases (13%) exhibited clonal dynamics that contrasted with the dominant pattern. In these cases (FL1009 and FL1020—both untreated and both with relatively short times to transformation, 0.39 y and 0.78 y, respectively), the dynamic properties showed conserved clonal architecture (FL1009) or only modest dynamics (FL1020). Thus, a small minority of cases may already contain the properties driving transformation at the time of diagnosis. Together, these results reveal a striking pattern of clonal dynamics underpinning histological transformation in the majority of TFL cases, independent of time to transformation and treatment regimen.

### Clones Dominant in Progressed Samples Were Prevalent in Diagnostic Samples

**Fig 6:**
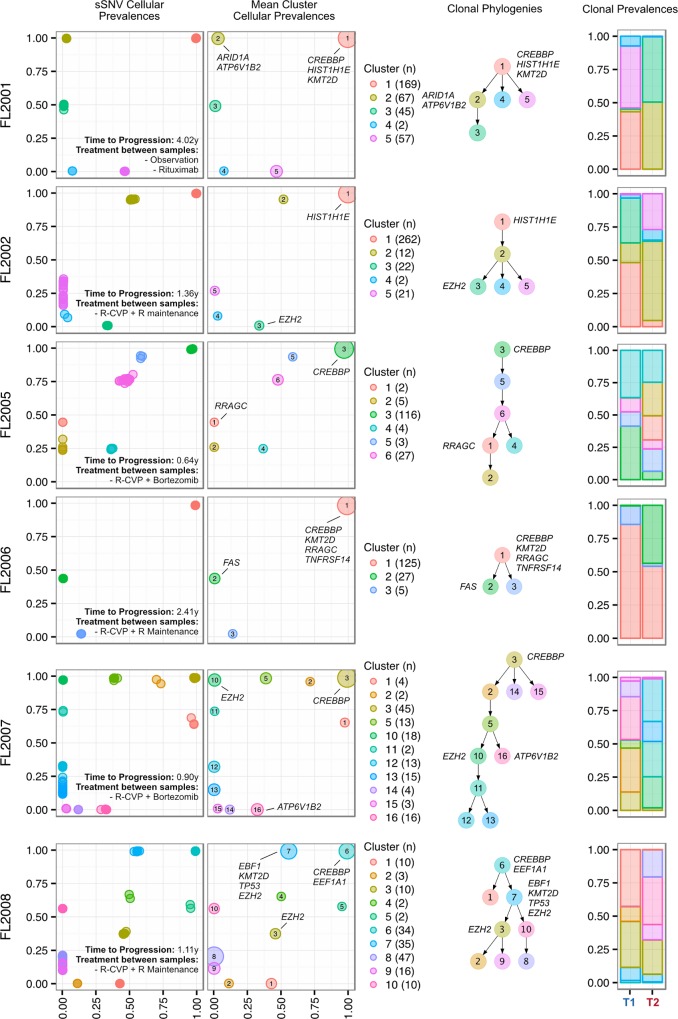
Clonal phylogenies of progressed follicular lymphoma samples. From mutation cellular prevalences to clonal phylogenies and clonal prevalences for each PFL patient. For each given patient, the leftmost plot shows the PyClone cellular prevalence of each validated sSNV (i.e., somatic in the T1 and/or T2 sample) at T1 (*x*-axes) and T2 (*y*-axes), with each mutation colored by the cluster it belongs to. The next plot to the right represents the cluster cellular prevalence (mean cellular prevalence of all mutations in the cluster), with the size of the dot representing the number of mutations in the cluster. This is followed by a clonal phylogeny and then a stacked bar plot representing the clonal prevalence of each clone in the T1 and T2 sample. The colors of the clusters have no meaning across patients. The *n* in parentheses beside the cluster color and number represents the number of sSNVs in that cluster. R, rituximab; R-CVP, cyclophosphamide, vincristine, and prednisone plus rituximab; sSNV, somatic single nucleotide variant; PFL, progressed follicular lymphoma.

Progressed samples exhibited patterns of clonal dynamics markedly different from those of transformed cases (Fig 6). Four cases (FL2002, FL2005, FL2007, and FL2008) harbored readily detectable clones at T1, which expanded to full clonal prevalence during treatment with immunochemotherapy. This suggests that clones harboring treatment resistance properties were already present at diagnosis, and that symptomatic disease progression may be attributed to selection of clones that were major constituents of the diagnostic sample. This mode of progression is reminiscent of the clonal evolution described in chronic lymphocytic leukemia, another mature, incurable, and typically relapsing lymphoid malignancy [15,16]. FL2006 showed a slightly different pattern whereby the ancestral clone dominated the T1 and T2 samples but was accompanied by modest dynamics, including expansion of a low prevalence clone (cluster 2) in the T2 sample. An exceptional case (FL2001) in the PFL group exhibited dynamics similar to those of TFL cases (validated with digital droplet PCR; Figs 5C, S6D and S6E), with a T2-specific lineage with *ARID1A* mutation (clusters 2 and 3) coming to dominate the relapse sample and with no evidence of the T1 clones (clusters 4 and 5). This patient initially presented with indolent FL, received single agent rituximab, and presented 4 y after diagnosis with symptomatic, progressive lymphoma unresponsive to three lines of systemic therapy, leading to the patient’s death. In this case, the phylogenetic structure was analogous to the TFL pattern, yet the biopsy from T2 showed no evidence of large cell transformation. Thus, treatment resistance patterns accompanied by significant clonal dynamics can occur in FL in the absence of overt transformation. PFL clonal dynamics suggest that progression on therapy is driven by a starkly different mode of evolution than what was observed for TFL. These two clinical end points are likely underpinned by non-overlapping evolutionary mechanisms, with PFL harboring intrinsically resistant properties at diagnosis and TFL generally acquiring the dominant transformation phenotype after diagnosis.

### Transformed Follicular Lymphoma Clonal Dynamics Are Inconsistent with Neutral Evolution

**Fig 7:**
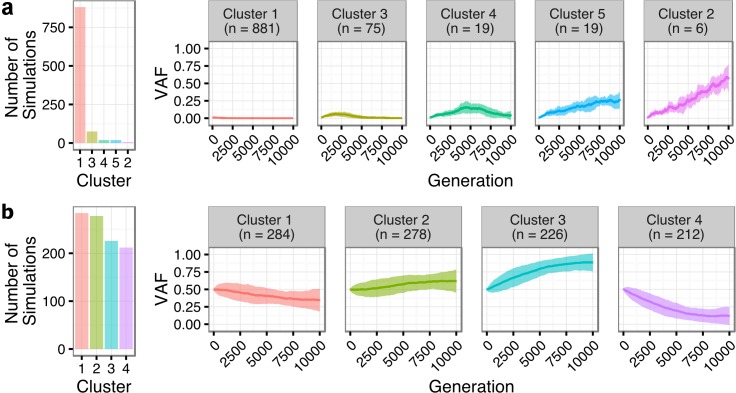
Tumor evolution modeling in transformed and progressed follicular lymphoma patients. Genetic drift modeling in TFL (A) and PFL (B) patients with an initial variant allele frequency of 1% and 50%, respectively. The far left bar plot indicates the number of simulations that follow a specific genetic drift trajectory (shown on the right). PFL, progressed follicular lymphoma; TFL, transformed follicular lymphoma; VAF, variant allele frequency.

We next sought to quantify the statistical likelihood of observing the clonal expansion of an extremely rare clone at T1 (<1%) into a dominant clone at T2 (>50%) under the assumption of neutral evolutionary dynamics for TFL patients. We modeled drift in 1,000 independent simulations under the Wright-Fisher process, to simulate the pattern of allelic “drift” without selection in asexually reproducing systems. The majority (88.1%) of the simulations resulted in an eventual loss of the mutant allele (cluster 1 of Fig 7A). Conversely, only six (0.6%; cluster 2) of the simulations exhibited a trajectory similar to the clonal expansion patterns observed in the TFL patients. As such, observing this clonal expansion pattern in 13 out of 15 TFL patients is statistically unlikely (binomial exact test *p <* 0.001) when assuming 0.6% as the expected trajectory rate. Modeling drift in PFL starting with a dominant clone at T1, the simulations demonstrate trajectories that are consistent with the observed patterns of evolution in PFL patients (Fig 7B). These results are consistent with the notion that histological transformation is driven through positive selection in the T1–T2 interval in TFL patients. In contrast, the clonal dynamic patterns between T1 and T2 in PFL patients are consistent with Wright-Fisher allelic drift without selection, suggesting that clones at T1 are not expanding under positive selection, despite treatment intervention.

### Contribution of Individual Gene Mutations to Transformation

Evolutionary analysis suggested several patterns of mutation acquisition (S8 Fig) including *TNFRSF14*, *CREBBP*, and *GNA13* mutations as predominantly ancestral (in the top level node of the clone phylogeny in 7/7, 12/13, and 5/5 mutations, respectively). *KMT2D*, *BCL6*, *HIST1H1E*, and *EZH2* mutations showed evidence of being ancestral in some cases and descendant (lower than the ancestral node) in others. The plasticity across ancestral and descendant states for recurrent gene mutations prompted us to resolve their etiology in a larger series of cases (extension cohort; 395 genomic DNA samples [T1 or T2] from 277 patients) and assess their roles in transformation and early progression (S5 and S6 Tables). Ninety-four genes were sequenced in this cohort (see S1 Supporting Appendix).

**Fig 8:**
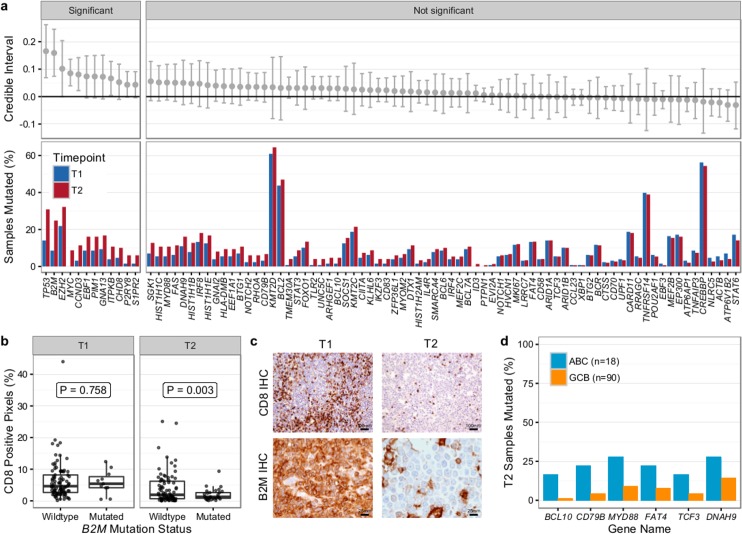
Results from targeted sequencing of 86 genes in samples from 159 transformed follicular lymphoma patients (128 T1 and 149 T2 samples). (A) Credible intervals from Bayesian proportion test (top). Genes are ranked by group difference and separated based on whether the probability of a given gene to be more commonly mutated in T2 is >0.95 or not. The percentage of samples harboring mutations in given genes is given at the bottom. (B) CD8-positive pixel count by Aperio automated imaging of immunohistochemically stained tissue cores, by time point and *B2M* mutation status. Only cases with paired information on CD8+ T cell scoring and mutation status are shown. (C) Representative microscopy images taken at 200× or 800× magnification (CD8 and B2M, respectively). (D) Proportion of mutated samples by cell of origin (activated B-cell-like [ABC] or germinal center B-cell-like [GCB]); shown are only genes that are significantly associated with either subtype (Fisher *p <* 0.05). IHC, immunohistochemistry.

We first compared T1 (*n* = 128) and T2 (*n* = 149) samples of transformed cases from 159 patients (118 paired biopsies). Similar to our findings from WGS (Fig 2B), mutational load in 86 genes in which the entire coding sequence was assessed was higher in T2 than in T1 samples (mean number of mutated genes 12.47 ± 6.80 versus 9.39 ± 5.74, Student *t* test *p* < 0.001) (S9 Fig). Mutation burden in the 5′ regions of 20 genes that are targets of somatic hypermutation did not significantly differ between the T1 and T2 samples, with the exception of *MYC* and *TMSB4X* (S10 Fig). We determined which genes had a higher likelihood of being mutated in T2 compared to T1 using a Bayesian proportion test and found 12 genes to be more commonly altered in transformed lymphoma (Fig 8A). These included previously described genes associated with transformation, such as *TP53*, *B2M*, *MYC*, and *EBF1*, as well as novel genes (e.g., *EZH2*, *CCND3*, *PIM1*, and *ITPKB*). *B2M* mutations were associated with a significantly reduced CD8+ T cell infiltrate in transformed lymphoma biopsies (Fig 8B and 8C). Moreover, mutations in *GNA13*, *S1PR2*, and *P2RY8*, all implicated in dissemination of germinal center B cells [40], were enriched in T2 samples. These findings suggest that defective DNA damage response, increased proliferation, escape from immune surveillance, and loss of confinement within the germinal center represent key features that drive histological transformation from indolent to aggressive lymphoma.

We next overlaid mutation status with detailed histological annotation. Composite morphology was associated with a lower prevalence of *TP53* mutations (8% versus 37%, Fisher *p* = 0.007) relative to diffuse large B cell lymphoma morphology. In addition, cell-of-origin classification was available for 108 cases with diffuse large B cell lymphoma histology, 18 and 90 of which were ABC and GCB subtype, respectively. More *BCL10* (16% versus 1%, Fisher *p* = 0.004), *CD79B* (22% versus 4%, Fisher *p* = 0.005), and *MYD88* mutations (28% versus 9%, *p* = 0.006) were observed in ABC TFL relative to GCB TFL (Fig 8D), suggesting that B cell receptor and NF-κB signaling are important contributors to the ABC phenotype in TFL.

### Gene Mutations in Early Progression Follicular Lymphoma

Next, we assessed the association of gene mutations with patient outcome, contrasting patients with early progression (<2.5 y after starting rituximab chemotherapy) (*n* = 41) and patients with late/never progression (no progression for >5 y) (*n* = 84). Samples from patients with early progression were enriched for high-risk clinical factors including poor performance status, tumor mass ≥ 7 cm, elevated lactate dehydrogenase, and high-risk FLIPI score (S6 Table). Median overall survival was extremely poor in these patients (3.01 y versus not reached in patients with late/never progression, log-rank *p <* 0.001; S11 Fig), highlighting the critical need for identifying these patients upfront.

**Fig 9:**
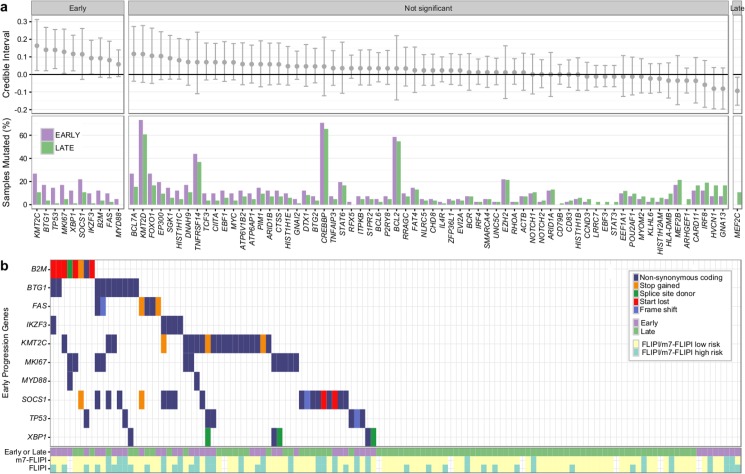
Results from targeted sequencing of 86 genes in 41 patients with early progression and 84 patients with late/never progression. (A) Credible intervals from Bayesian proportion test (top). Genes are ranked by group difference and separated based on whether the probability of a given gene to be more commonly mutated in patients with early progression is >0.95 or not. The percentage of samples harboring mutations in given genes is given at the bottom. (B) Oncoplot of genes associated with early progression, annotated with FLIPI and m7-FLIPI risk groups. FLIPI, Follicular Lymphoma International Prognostic Index.

Overall, the burden of somatic hypermutation was not significantly different in samples from patients with early versus late/never progression, but samples from patients with early progression had more mutations per sample in *BACH2*, *BTG2*, *RHOH*, and *SOCS1*, and fewer mutations in *LTB*, when compared to patients with late/never progression (S12 Fig). Patients with early progression had, on average, a higher mutation load in those 86 genes in which the entire coding sequence was assessed, when compared to patients with late/never progression (13.44 ± 9.17 versus 9.75 ± 5.71, Student *t* test *p* = 0.022) (S13 Fig). Ten genes were mutated more commonly in patients with early progression than in patients with late/never progression, including *KMT2C*, *TP53*, *BTG1*, *MKI67*, *XBP1*, and *SOCS1* (Fig 9A). Only *MEF2C* was more commonly mutated in patients with late/never progression. Overall, 33 out of 41 patients with early progression (80%) had mutations in any of the ten early-progression-associated genes, but none of the early-progression-associated genes were mutated at a frequency > 27% (Fig 9B). Thus, early progression appears to be related to relatively infrequent genetic alterations. Furthermore, none of the early-progression-associated gene mutations form part of the m7-FLIPI outcome predictor, and, in our cohort that was enriched for clinical extremes, the m7-FLIPI was similarly associated with early progression when compared with the FLIPI, but not superior, having better specificity (88% versus 76%) but worse sensitivity (36% versus 63%). Taken together, our results identify early progression as a distinct clinicogenetic disease category that is imperfectly captured by traditional prognostic tools.

## Discussion

**Fig 10:**
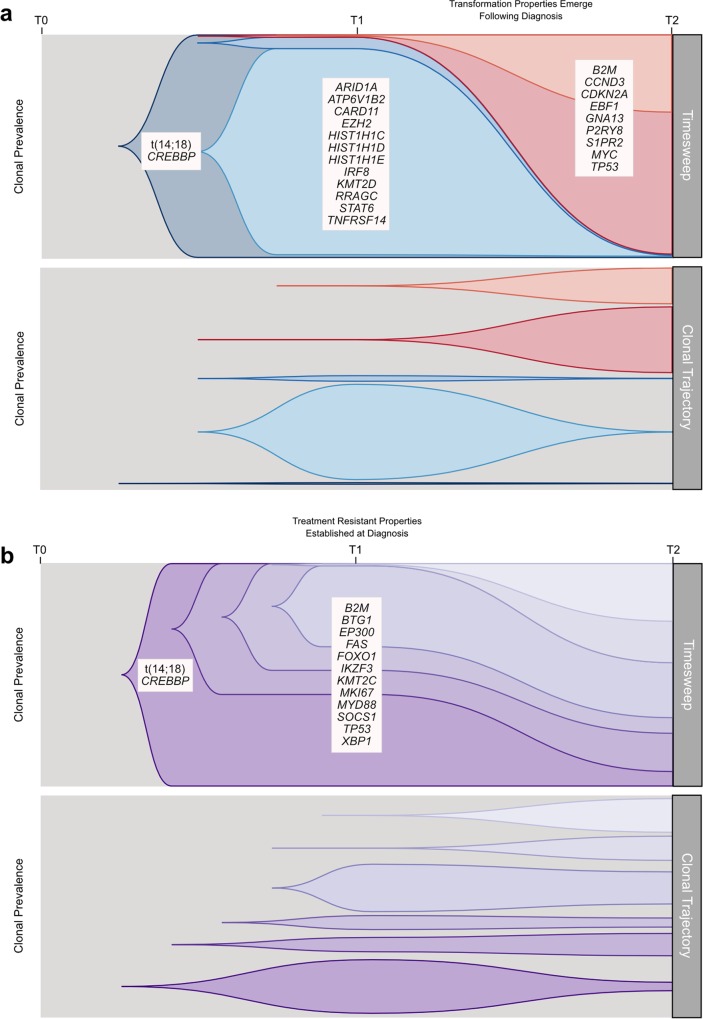
Schematic models of evolutionary progression in transformed and progressed follicular lymphoma. (A) The timesweep facet (top) shows the diagram of a prototypical transformed case in follicular lymphoma, indicating dynamics of clonal composition from germline to diagnostic T1 specimen to histologically transformed T2 specimen. The clonal trajectory facet (bottom) presents an alternative view of the timesweep facet, without the clonal hierarchy, demonstrating the trajectory of the individual clones over time. Listed genes include those found to be predominantly enriched at one time point in our study and/or in the literature. (B) The timesweep facet (top) shows the diagram of a prototypical follicular lymphoma case that progressed on treatment, indicating dynamics of clonal composition from germline to diagnostic T1 specimen to progressed T2 specimen, with the trajectory of each clone presented in the clonal trajectory facet (bottom).

We established that transformation and progression in FL are driven by disparate modes of evolutionary change. Shown schematically in Fig 10, TFL is characterized by the emergence of clones that become dominant at T2 and that typically lie below the detection limit of even highly sensitive methods at the T1 (FL) time point (Fig 10A), implying that the aggressive phenotypes emerge after diagnosis. By contrast, early progression of FL commonly results from prevalent clones at T1, such that much of the clonal architecture is maintained despite treatment, implying that resistant properties are well established at diagnosis (Fig 10B). The content of gene mutations associated with transformation and early progression also differed. We found novel associations of gene mutations with transformation (including *CCND3*, *GNA13*, *S1PR2*, and *P2RY8* mutations) and showed that TFL is molecularly heterogeneous, with, for example, the ABC subtype of TFL being enriched for *BCL10*, *CD79B*, and *MYD88* mutations. Genes with recurrent mutations associated with early progression included *KMT2C*, *TP53*, *BTG1*, and *MKI67*. Thus, transformation and progression can be attributed to disruption of different biological processes.

Our study has several limitations. The small number of cases assessed in the WGS cohort, the lack of reliable copy number information in our extension cohort, and the absence of an additional validation cohort to confirm the prognostic implication of gene mutations associated with early progression provide direction for future complementary follow-up studies. Our findings are nonetheless of critical translational relevance. The divergent modes of evolution of PFL and TFL mirror distinct differences in the clinical presentation of these entities, with transformation being uniquely associated with rapid onset of tumor growth and systemic symptoms, suggesting an underlying shift in tumor biology. As the nature of expansion appears to correlate with rapid-onset symptoms, more granular monitoring of these patients would help to determine the exact timing of the evolutionary inflection point. Furthermore, the defining genetic features of transformation may remain elusive at diagnosis, and at best will require ultra-sensitive detection techniques in order to develop predictive assays. Technical improvements in limits of detection may yet reveal that T2 alleles are always detectable in T1 samples, but our deeply sampled data presented here indicate this may remain a challenge and cannot rule out the possibility that T2 clones arise after diagnosis in some cases. Conversely, primary resistance to upfront combined modality therapy generally occurs by the selection of resistant clones readily found at diagnosis, suggesting that their detection may predict resistance to treatment. In that regard, samples from patients who experience early progression harbor relatively uncommon gene mutations that are associated with early progression (e.g., *KMT2C*, *TP53*, *BTG1*, *MKI67*, and *XBP1* mutations), most of which have not previously been described to predict progression.

Our results have fundamental implications for the study of tumor evolution. Paradoxically, several patients who were managed solely with observation exhibited punctuated clonal dynamics, whereas PFL patients who were treated with multi-agent therapy exhibited relative stability in their clonal make-up. This implies that the evolutionary processes driving FL may be independent of selective pressures imposed by treatment regimens. The association of known driver events (such as *CCND3* mutations) with transformation suggests that such punctuated expansions typical of transformation are under positive selection. This argues against fixation under neutral selection models, which would suggest gradual shifts over protracted periods, for example, under the assumption of emergent neutrality [41]. Rather, in transformation, it is likely that specific alleles overcome offsetting interactions between beneficial and deleterious mutations acquired over time due to increased fitness. Indeed, three cases in the WGS cohort with *CCND3* descendant mutations had widely varying time to transformation: 14.57 y, 5.05 y, and 2.56 y. These mutations showed variant allele frequencies of 0, 0.002, and 0, respectively, at diagnosis and thus emerged from extremely rare populations. Learning precisely how alleles such as *CCND3* mutations exhibit epistatic interactions and modify the effect of founder events such as the t(14:18) translocation to confer higher fitness will be critical to elucidating the mechanism of histological transformation. The pattern is dramatically different in progression, where we might expect clonal dynamics in the presence of a shifting fitness landscape induced by therapy. Rather, clonal architecture at diagnosis remains relatively constant, suggesting that fitness could be attributed to non-genetic factors or that these tumors acquire resistance properties very early in their evolutionary histories and in the absence of therapeutic selective pressure.

Our results place transformation and progression in FL at the extremes of the clonal population dynamics spectrum, at once informing future management strategies and stimulating deeper questions on how FLs mechanistically navigate varied fitness landscapes.

## Supporting Information

S1 Supporting Appendix Full supporting information. This is the full supporting information that includes full details of specimen acquisition, data analysis, supporting figures, and figure/table legends.
(PDF)

S1 Fig Sample overview and timeline of whole genome sequencing cohort. (PDF)

S2 Fig Whole genome sequencing statistics. (PDF)

S3 Fig Correlation of mutation load difference with time between samples. (PDF)

S4 Fig Whole genome sequencing variant allele frequency distribution across all transformed and progressed follicular lymphoma patients. WGS T2 versus T1 variant allele frequencies for TFL and PFL patients. The resulting fraction of predicted time point-specific mutations is listed in the bottom right-hand corner of each patient plot (Shared; T1; T2).
(PNG)

S5 Fig Clonal phylogenies of transformed follicular lymphoma samples. From mutation cellular prevalences to clonal phylogenies and clonal prevalences for TFL patients not represented in Fig 3. For each given patient, the leftmost plot shows the PyClone cellular prevalence of each validated sSNV (i.e., somatic in the T1 and/or T2 sample) at T1 (*x*-axes) and T2 (*y*-axes), with each mutation colored by the cluster it belongs to. The next plot to the right represents the cluster cellular prevalence (mean cellular prevalence of all mutations in the cluster), with the size of the circle representing the number of mutations in the cluster. This is followed by a clonal phylogeny and then a stacked bar plot representing the clonal prevalence of each clone in the T1 and T2 sample. The colors of the clusters have no meaning across patients. The *n* in parentheses beside the cluster color and number represents the number of sSNVs in that cluster.
(PDF)

S6 Fig Ultra-sensitive detection of low prevalence clones in T1 samples. Shown are five mutations (A–E) in four patients (FL1004, FL1012, FL1019, and FL2001) in which PyClone suggested that the expanded T2-dominant mutation clusters were present at near zero prevalence at T1. Background refers to the variant allele frequencies of all possible single nucleotide changes in the vicinity of the mutation of interest (defined as up to 50 base pairs upstream and up to 50 base pairs downstream). The results are confirmed by digital droplet PCR (rightmost plots). Color coding in the digital droplet PCR plots is as follows: grey = empty droplets; blue = single-positive droplets for wild-type allele; purple = double-positive droplets; red = single-positive droplets for mutant allele.
(PDF)

S7 Fig Transformed follicular lymphoma capture sequencing PyClone mutational cellular prevalence trajectories. (PDF)

S8 Fig Ancestral and derivative mutations. Proportion of gene mutations that are ancestral or derivative based on clonal analysis of the whole genome sequencing cohort (transformed and progressed cases). Shown are only genes that are significantly mutated based on a MutSigCV *q-*value < 0.05 in the combined analysis of our data and the data from Okosun et al. [20] and Pasqualucci et al. [21].
(PDF)

S9 Fig Mutational load in the coding sequence of 86 genes in T1 and T2 samples from transformed follicular lymphoma cases. Non-synonymous single nucleotide variants as well as small insertions or deletions were considered in this analysis.
(PDF)

S10 Fig Mutations in regions of somatic hypermutation in follicular lymphoma and transformed follicular lymphoma samples. (A) Number of mutations per sample found in 20 genes, by time point (128 FL samples, 149 TFL samples; total number of patients, *n* = 159). (B) Number of mutations per sample, by gene and by time point.
(PDF)

S11 Fig Progression-free and overall survival in patients with early versus late/never progression. (PDF)

S12 Fig Mutations in regions of somatic hypermutation in samples from patients with early versus late/never progression. (A) Number of mutations per sample found in 20 genes, by outcome category (41 patients with early progression, 84 patients with late/never progression). (B) Number of mutations per sample, by gene and by outcome category.
(PDF)

S13 Fig Mutational load in the coding sequence of 86 genes in T1 samples from patients with early versus late/never progression. Non-synonymous single nucleotide variants as well as small insertions or deletions were considered in this analysis.
(PDF)

S14 Fig Bioinformatics workflow for predicting somatic single nucleotide variants from whole genome sequencing data. (PDF)

S15 Fig Tumor content estimation in transformed and progressed follicular lymphoma patients. A scatterplot of the mean T2 versus T1 variant allele frequency of each cluster (identified from variational Bayesian binomial mixture model [VBBMM] clustering) in each TFL and PFL patient, with the size of the cluster representing the number of sSNVs in the cluster. The cluster most representative of clonally dominant diploid heterozygous sSNVs in each patient is indicated by an asterisk in the patient legend. Tumor content is calculated by multiplying the mean variant allele frequency of this cluster by two in each time point. The resulting predicted tumor content is listed in the bottom right-hand corner of each patient plot (T1; T2).
(PDF)

S16 Fig Tumor content estimation in non-progressed follicular lymphoma patients. T1 variant allele frequency density plots of each cluster (identified from a VBBMM). The cluster most representative of the diploid heterozygous sSNVs in each patient is indicated by an asterisk in the patient legend.
(PDF)

S17 Fig Bioinformatics workflow for predicting somatic copy number alterations from whole genome sequencing data. (PDF)

S18 Fig Selection of somatic single nucleotide variant positions for deep sequencing validation. At least 192 positions were selected for deep sequencing validation. This selection included all coding sIndels and non-synonymous coding sSNVs (B), as well as synonymous coding sSNVs (C). To backfill positions to meet the 192-position requirement, we then proportionally sampled non-coding sSNVs from the different clusters (D) identified by VBBMM (A) of the T1 and T2 variant allele frequencies.
(PDF)

S19 Fig Inference of clonal phylogenies from sequencing data workflow. sSNVs, predicted from the T1 (*x*-axis) and/or T2 (*y*-axis) samples from whole genome sequencing (A), were selected for targeted deep sequencing validation (B). Validated positions were used as input into PyClone to determine the mutational cellular prevalence of each sSNV (C). sSNVs with similar mutational cellular prevalences were clustered with PyClone, with each cluster’s cellular prevalence being represented by the mean cellular prevalence of all sSNVs in the cluster (D). These cluster cellular prevalences were used as input into Citup to construct clonal phylogenies (E) and clonal prevalences for the T1 and T2 samples (F). sSNVs in each node were propagated down to their children nodes.
(PDF)

S20 Fig Bioinformatics workflow for predicting somatic copy number alterations from capture sequencing data. (PDF)

S1 Table Whole genome sequencing alignment summary statistics. Sequencing statistics for each WGS library generated by Picard tools CollectAlignmentMetrics and CollectWgsMetrics.
(XLSX)

S2 Table Sample information and pathological and clinical annotation. (XLSX)

S3 Table TITAN copy number state overview. States from TITAN are collapsed in one of ten different summary states listed in the state summary column for analysis.
(XLSX)

S4 Table Targeted deep amplicon sequencing, PyClone, and Citup results. (XLSX)

S5 Table Clinical characteristics of transformed follicular lymphoma patients. (XLSX)

S6 Table Clinical characteristics of patients with early versus late/never progression. (XLSX)

S7 Table TITAN final selected parameters. (XLSX)

S8 Table Germline copy number variation mask. Germline copy number variation segments found from normal samples in METRABRIC [42], the Database of Genetic Variants, and peripheral blood lymphocyte samples [43].
(XLSX)

S9 Table Primers and probes for digital droplet PCR. (XLSX)

S10 Table Capture sequencing gene panel (86 genes, coding region sequenced). Genes in which the coding sequence was sequenced and analyzed in the extension cohort, and criteria by which they were selected.
(XLSX)

S11 Table Capture sequencing gene panel (20 genes, 5′ regions sequenced). Genes in which the 5′ sequences were analyzed for coding and non-coding mutations.
(XLSX)

S12 Table Capture sequencing alignment summary statistics. Sequencing statistics for each capture sequencing library generated by the Picard tool CalculateHsMetrics.
(XLSX)

S1 STROBE Checklist (DOC)

## Abbreviations

ABC: activated B-cell-like
FL: follicular lymphoma
FLIPI: Follicular Lymphoma International Prognostic Index
GCB: germinal center B-cell-like
NPFL: non-progressed follicular lymphoma
PFL: progressed follicular lymphoma
sCNA: somatic copy number alteration
sIndel: somatic small insertion or deletion
sSNV: somatic single nucleotide variant
TFL: transformed follicular lymphoma
VBBMM: variational Bayesian binomial mixture model
WGS: whole genome sequencing
