# Supplementary material for: Histological Transformation and Progression in Follicular Lymphoma: A Clonal Evolution Study
Source: PLoS Med. 2016 Dec 13;13(12):e1002197. doi: 10.1371/journal.pmed.1002197 (PMC5154502; doi:10.1371/journal.pmed.1002197)
Supplement: S1 Supporting Appendix — This is the full supporting information that includes full details of specimen acquisition, data analysis, supporting figures, and figure/table legends. (PDF) [file pmed.1002197.s001.pdf]

Supporting Appendix: Histological Transformation and Progression in Follicular Lymphoma: a Clonal Evolution Study

Contents

1 Specimen acquisition and sample preparation **5**

2 Pathology **6**

3 Availability of sequencing data **6**

4 Whole genome sequencing data analysis **6**

4.1 Library construction, alignment, and filtering 6

4.2 Somatic single nucleotide variant and small insertion-deletion predictions 7

4.3 Tumor content estimation 8

4.4 Somatic copy number alteration prediction 9

4.5 Somatic structural rearrangement prediction 10

5 Digital droplet PCR **11**

6 Targeted deep amplicon sequencing data analysis **12**

6.1 Selection of positions for deep amplicon sequencing 12

6.2 Primer design, library construction, and alignment 12

6.3 Somatic single nucleotide variant validation 12

7 Clonal dynamics analysis **13**

7.1 Estimation of mutational cellular prevalences 13

7.2 Estimation of clonal prevalence and phylogenies 15

7.3 Filtering of PyClone clusters for clonal phylogeny construction 15

8 Neutral evolution modeling **16**

9 Capture-based targeted sequencing **16**

9.1 Capture panel and library construction . . . 16

9.2 Alignment and sequencing statistics . . . 17

9.3 Somatic single point mutations analysis . . . 17

9.4 Copy number analysis . . . 18

9.5 Determination of mutational cellular prevalence . . . 18

10 Code availability **19**

1 Specimen acquisition and sample preparation

The whole-genome sequencing (WGS) cohort consists of 41 patients that were selected to fall into three groups: 1) A "Transformed" group of 15 patients (TFL) diagnosed with FL and subsequent or concomitant (patient FL1014) transformation to large cell lymphoma; these cases were selected irrespective of type of treatment received. 2) A "Progressed" group of 6 patients (PFL) that were diagnosed with FL and subsequently experienced progressive disease without histological evidence of transformation; 5 out of these 6 patients progressed within 2.5 years after starting first-line immuno-chemotherapy with R-CVP (rituximab, cyclophosphamide, vincristine and prednisone). 3) A "Good outcome" group of 20 patients (NPFL) who were diagnosed with FL and did not experience progression for at least 5 years. All cases were selected irrespective of clinical stage, grade and t(14;18) translocation status in order for the cohort to be reflective of the clinical and pathological heterogeneity that is inherent to FL. Samples with a tumor content of less than 50% and available frozen single cell suspensions were flow-sorted to purify tumor (CD19+ kappa or lambda+ CD3-) and germline cells (CD19- kappa or lambda- CD3+). Germline DNA was obtained from flow-sorted CD3+ lymphocytes or from peripheral blood cells. All germline samples were confirmed to be free of tumor contamination by the absence of PCR-amplifiable patient-specific t(14;18) and/or VDJ rearrangements.

The capture sequencing cohort refers to the samples from 277 patients (39 patients overlapping with the WGS cohort), in which germline DNA was available for 80 patients. These patients were divided into three groups: 1) A "Transformed" group of 159 patients (sample at primary FL timepoint available in 128 cases, sample at transformed FL timepoint available in 149 cases and samples from both timepoints available in 118 cases). 2) A "Progressed" group of 41 FL samples from patients who presented with early progressive disease within 2.5 years after starting immuno-chemotherapy with R-CVP. Progression was defined as radiological evidence of progressive disease and requirement for initiation of second-line therapy. 3) A "Good outcome" group of 84 patients without progression for at least 5 years after either observation or R-chemotherapy and R maintenance.

For both whole-genome sequencing and capture sequencing, DNA was extracted from frozen tissue or single cell suspensions using Qiagen DNA/RNA AllPrep kits. Timepoint specificity is indicated for each sample by the suffixes -T1 for the primary timepoint (by definition FL) or -T2 for the secondary timepoint in the TFL or the PFL cohorts (transformed or treatment-resistant FL). Clinical annotation for all samples can be found in **S2 Table**, **S6 Table**, and **S5 Table**.

2 Pathology

All samples were centrally reviewed by expert hematopathologists at the BC Cancer Agency (AM, PF, KT, RDG), and the following histopathologic co-variates were recorded: FL grade (1, 2 or 3A), histological diagnosis at transformation (DLBCL, composite or B-cell lymphoma not otherwise classifiable (BCLU)), cell of origin for all TFL cases with a DLBCL histology, and immunohistochemistry for expression of TP53, IRF4, CD8, and B2M. Composite histology was defined as any evidence of underlying low grade lymphoma in a sample that concomitantly harbored large cell lymphoma. The Lymph2Cx assay was performed as previously described [1,2], with the exception that it was applied in 4 cases to RNA extracted from fresh-frozen blocks, using 100 ng as input. Immunohistochemical stained slides for the T cell marker CD8 (antibody clone C8/144B, Dako, catalogue number M7103) were scanned with an Aperio ScanScope XT at 20x magnification. Analysis was performed using the Aperio ImageScope viewer (v12.1.0; Aperio Technologies). Only cores and areas containing tumor were scored by applying the Positive Pixel Count algorithm with an optimized color saturation threshold. Any staining was considered positive and the number of positive pixels was divided by the total pixel count. Scores from both cores were subsequently averaged and multiplied by 100 to obtain the percentage of positive pixels. Images from tissue cores stained for CD8 and B2M (rabbit polyclonal antibody, Dako, catalogue number A0072) were taken using a Nikon Eclipse 80i microscope equipped with a Nikon DS-Ri1 camera and NIS Elements Imaging Software, D3.10.

3 Availability of sequencing data

Genome data is available at the European Genome-phenome Archive (`http://www.ebi.ac.uk/ega`) under accession number EGAS00001001709.

4 Whole genome sequencing data analysis

4.1 Library construction, alignment, and filtering

Whole genome sequencing (WGS) libraries were constructed from genomic DNA using PCR-free library construction protocols, with the exception of libraries from cases FL1001, FL1002, FL2001, and FL2002 that were constructed during an earlier phase of the project using PCR-containing protocols. Libraries were sequenced on Illumina HiSeq 2500 instruments, generating on average 1822261237 paired-end sequence reads of 100-125 length per library. Libraries were aligned and processed using the standard Canada's Michael

Smith Genome Sciences Centre WGS alignment pipeline. The BWA (v0.5.7) aligner [3] was used to align the paired-end reads to the human reference genome GRCh37 (`http://www.bcgsc.ca/downloads/genomes/9606/hg19/1000genomes/bwa_ind/genome/GRCh37-lite.fa`) . PCR duplicates were marked using Picard tools (v1.126) using MarkDuplicates and sequencing statistics (**S2 Fig**; **S1 Table**) were collected using CollectWgsMetrics and CollectMultipleMetric. No joint realignment of the reads within this cohort was performed.

While assessing the number and type of somatic single nucleotide variant (sSNV) substitutions across all our samples, it became apparent that samples FL1005T1, FL1009T1, FL1009T2, FL1012T1, FL1012T2, FL1014T1, FL1014T2, FL2005T1, FL2005T2 and FL3014T1 had an overwhelming representation of C to A substitutions at low allelic ratios (between 10-15%). The cases appeared to be randomly affected. The low allelic ratio C to A substitutions were documented to be artifactual by amplicon sequencing in sample FL1007T1 (data not shown) and deemed to be compatible with oxidative damage during DNA shearing, as described by Costello et al. [4] We therefore filtered these positions out by applying allelic ratio filters for C to A substitutions in the affected samples (filter set at 0.20 for FL1005T1, FL1009T1, FL1009T2, FL1012T2, FL1014T1, FL3014T1 and at 0.25 for FL1012T1, FL1014T2, FL2005T1, FL2005T2).

All sequencing data will be deposited at the European Genome-phenome Archive (`http://www.ebi.ac.uk/ega`) under accession number EGAS00001001709.

4.2 Somatic single nucleotide variant and small insertion-deletion predictions

The full bioinformatics workflow for predicting somatic single nucleotide variants (sSNVs) from the whole genome sequencing data is shown in **S14 Fig**. MutationSeq [5] (v4.1.0) was used to predict sSNVs for each tumor-normal pair using the parameters "-m model_v4.0.2.npz". To increase the sensitivity for sSNVs, Strelka [6] (v1.0.13) was also used to predict sSNVs for each tumor-normal pair using the default parameters. Mutations reported by MutationSeq and/or Strelka (i. e. appeared in the passed.somatic.snvs.vcf file) were aggregated to form a timepoint-specific list of candidate sSNV positions. For TFL and PFL patients, a patient-centric candidate list of sSNV positions was generated by aggregating the candidate sSNVs positions across both timepoints (i.e. T1 + T2 candidate sSNVs). For NPFL, the patient-centric candidate list is equivalent to the T1 candidate list as there is only one timepoint sample.

MutationSeq was then re-run specifically interrogating the patient-centric sSNV candidate list across both timepoints (for TFL and PFL patients) and single timepoint for NPFL patients. This effectively allowed us to retrieve sSNV information at candidate positions predicted by MutationSeq and Strelka in a consistent

output format, and also across timepoints. Final putative timepoint centric sSNVs lists were constructed based on the following criterion: 1) the sSNV had a MutationSeq probability >= 0.9 and MutationSeq filter field = "PASS", 2) the sSNV was predicted by Strelka and MutationSeq filter field = "PASS", or 3) sSNV was predicted by Strelka, MutationSeq filter = "INDL" and MutationSeq probability >= 0.9.

SnpEff (v3.5) was then used to annotate each sSNV with respect to the canonical transcript with the parameters "-canon -no-downstream -no-intergenic -no-upstream" using the GRCh37.72 SnpEff database. In the scenario where a position may have multiple SnpEff effects, the effect with the most impact was chosen. The effect impact was ordered as follows from lowest to highest: 1) intragenic, 2) intron, 3) exon, 4) utr 3 prime, 5) utr 5 prime, 6) splice site region, 7) synonymous coding, 8) synonymous stop, 9) non synonymous start, 10) splice site donor, 11) splice site acceptor, 12) non synonymous coding, 13) stop lost, 14) stop gained, 15) start lost. For downstream analyses, coding sSNV effects were considered to be: 1) non synonymous coding, 2) stop gained, 3) start lost, 4) stop lost, 5) splice site donor, 6) splice site acceptor, 7) non synonymous start, and 8) splice site region. Truncating effects were considered to stop gained and start lost.

The same runs of Strelka for predicting sSNVs also predicted small somatic insertions and deletions (sIndels) with the passing results placed into the passed.somatic.indels.vcf file. These indels all passed the Strelka internal filters that are listed at `https://sites.google.com/site/strelkasomaticvariantcaller/home/somatic-variant-output`. SnpEff was used to annotate each sIndel with the same parameters as used in the sSNV annotations. For downtream analyses, coding sIndel effects were considered to be: 1) codon change plus codon deletion, 2) codon change plus codon insertion, 3) codon deletion, 4) codon insertion, 5) frame shift, 6) splice site acceptor, 7) splice site donor, and 8) splice site region.

4.3 Tumor content estimation

To estimate the tumor content of each tumor-normal pair, we used the sSNV results from the whole genome sequencing data. More specifically, we applied a variational bayes binomial mixture model clustering (VBBMM) on the sSNV allele count data of each patient. For TFL and PFL patients, we clustered in 2 dimensions (T1 and T2; **S15 Fig**). For NPFL patients, we clustered in 1 dimension (T1 only; **S16 Fig**). Next, we identified the cluster most representative of the clonally dominant diploid heterozygous mutations in each patient. To calculate the sample-specific tumor content, we took the mean variant allele fraction (VAF) of that cluster in that sample and multiplied by 2. These tumor/normal content estimation values were used as input into PyClone [7] and TITAN [8]. For TitanCNA, there were a few exceptions where

the tumor/normal content estimations from sSNV data are not used. See “Somatic copy number alteration prediction” in the Supplemental Appendix for specific details on these samples.

4.4 Somatic copy number alteration prediction

The full bioinformatics workflow for predicting somatic copy number alterations (sCNA) from the WGS data is shown in **S17 Fig**. Germline heterozygous single nucleotide polymorphisms (snp) in the normal sample were first predicted with samtools (v0.1.18) and bcftools (v0.1.18). Specifically, the normal bam file was first filtered for PCR duplicates and non-unique mapping reads using “samtools view -F 1024”. This filtered bam was used as input into “samtools mpileup -u -I -f GRCh37.fa” and then “bcftools view -vcg”. The positions are then filtered to only keep snps reported in dbSNP (v137) using “SnpSift filter ‘isHet(GEN[0])”’ (v3.5; available as part of the SnpEff package). Read count data for the reference and variant alleles were then retrieved for all these snp positions in both the tumor and normal bam files. Reads considered PCR duplicates and non-unique were filtered “samtools view -F 1024” and not considered in the allele read counting.

HMMcopy [9] (v0.1.1) was used to generate coverage wig files for the tumor and normal samples using a window size of 1000 base pairs (“readCounter -w 1000”). Additionally, HMMcopy was also used to calculate GC content of the GRCh37 genome (“gcCounter -w 1000”). Finally, a GRCh37 mappability file was generated by first running HMMcopy’s “generateMap -w 35” to generate a BigWig file which is used as input into HMMcopy’s “mapCounter -w 1000” to generate a final mappability wig file.

The tumor-normal pair’s read count, coverage data, and normal content estimations (see “Tumor content estimation” in the Supplemental Appendix for details on estimation) along with the GRCh37 GC content and mappability data were used as input into TITAN [8] bioconductor R package (named TitanCNA; v1.5.7) to predict sCNAs. The TitanCNA “loadDefaultParameters” function was used with the parameters “copyNumber = 4, numberClonalClusters = 1. . . 5, symmetric = TRUE” followed with setting ploidy = 2 to get the initial parameters. The numberClonalClusters was set to a value between 1 to 5 (more details below) Data was filtered for low and high depth positions (“filterData with parameters minDepth = 10, maxDepth = 200”).

TITAN was then run with runClonalEM with the parameters “maxiter = 20, maxiterUpdate = 1500, txnExpLen = 1e9, txnZstrength = 1e9, useOutlierState = FALSE, normalEstimateMethod = fixed, estimateS = TRUE, estimatePloidy = TRUE”). For each tumor-normal pair, TITAN was run 5 times with each run differing by the numberClonalClusters parameter as mentioned earlier. The run with the minimum S_Dbw_validity_index is chosen as the optimal model for each tumor-normal pair. In several tumor-

normal pairs (FL1001T2, FL1007T2, FL1008T2, FL1012T2, FL2008T1, FL3004T1, FL3006T1, FL3012T1, FL3015T1), the optimal model selection was not a pragmatic solution. In these pairs, we investigated the model for every numberClonalClusters value and selected the model selection with the most pragmatic results. For FL1001T1 and FL3012T1, the TITAN results were uninterpretable indicating that TITAN was having difficulty converging on a pragmatic solution. For these patients, we ran TITAN across a range of normal content initialization values (i.e. 0.1 to 1) to determine if normal content was contributing to the non-convergence. We discovered that a normal content of 1 as an initialization value gave a pragmatic solution and chose to go forward with the results of this initialization value. The final parameters values selected are listed in **S7 Table**.

The output TITAN results were then converted into TITAN segments using the "createTITANsegmentfiles.pl" script with each segment taking on 1 of 25 possible copy number states which were then collapsed into 1 of 10 possible summary states: 1) Homozygous Deletion, 2) Hemizygous Deletion, 3) Neutral, 4) 3N Gain, 5) 4N Gain, 6) 5N Gain, 7) 6N Gain, 8) 7N Gain, 9) 8N Gain, 10) Somatic LOH. These TITAN copy number states and summary state mappings are listed in **S3 Table**.

To increase our specificity for sCNAs, we masked our segment data with a copy number variant mask (mask is listed in **S8 Table**). This mask was constructed using the gold standard data from the Database of Genomic Variants (vJuly 2015), peripheral blood samples [10], and normal breast tissues (METABRIC [11]). TITAN segments were overlapped with the copy number variation mask and any segment that overlapped with >= 25% of its length with any mask segment was labeled as a mask segment and assigned a neutral summary state.

To assign gene-centric copy number, genes coordinates (Ensembl v72) were overlapped with the TITAN segment coordinates. Genes were considered overlapping with a TITAN segment if >= 50% of the gene overlapped with the segment and then would be assigned the state of the overlapping TITAN segment.

4.5 Somatic structural rearrangement prediction

Destruct [12] (v0.2.0) was used to predict structural rearrangements for each patient. For TFL and PFL patients, the normal, T1 and T2 samples were used as input into Destruct to simultaneously predict rearrangements across all samples. For NPFL patients, the normal and T1 samples were used. The following filters were applied for Destruct predictions: 1) 0 read in the matching normal sample, 2) distance to any other breakpoint is > 50 basepairs, 3) log likelihood > -20, 4) minimum template length > 120, 5) matescore ≤ 10, 6) both rearranged partners must be on an autosomal or sex chromosome, 7) not found as a variant

in the database of genomic variants database, and 8) number of split reads > 0 if the rearrangement does involve the IGH locus. When considering rearrangements that may affect a gene, the breakpoint must be inside the gene itself and not upstream or downstream of the gene. A rearrangement was considered to be shared if there was ≥ 1 read in both timepoints and timepoint specific if it contained 0 reads in one timepoint.

5 Digital droplet PCR

Digital droplet PCR (ddPCR) was performed on selected pairs of T1 and T2 biopsies. As controls, we used 200ng of genomic DNA from 3 reactive lymph node samples from unrelated individuals and the data from these samples was pooled. For the test samples, we used a minimum of 600ng and 200ng of genomic DNA for T1 and T2 samples, respectively. Primers and FAM- or HEX-tagged probes were designed by Integrated DNA Technologies (IDT) and their sequences are reported in **S9 Table**. PCR was performed in droplets generated using the Biorad QX200 droplet generator and each 20 μL reaction mix contained 900nM forward and reverse primers, 250nM FAM and HEX probes and 5 units of HindIII, in addition to 1x ddPCR Supermix for Probes (No dUTP) (Biorad), genomic DNA and water. For each pair of probes, the optimal annealing temperature was determined using a temperature gradient. PCR conditions were as follows: 95C for 10 minutes, (94C for 30 seconds, optimal annealing temperature for 90 seconds) x 39, 98C for 10 minutes. Droplets were assessed for FAM or HEX fluorescence using the Biorad QX200 droplet reader. In all instances, we verified that no signal for either wild-type or mutant DNA could be detected in the non-template control wells. We clustered results from T2 samples using Gaussian mixture models and the R package mclust (v5.1), setting the number of mixture components to 4 and initializing hierarchical clustering on a random sample of 1000 datapoints. If clustering using this approach did not reveal 4 distinct clusters corresponding do empty droplets, wild-type only droplets, mutant only droplets or double positive droplets, we repeated the clustering until an appropriate solution was found. The seed of the first successful clustering was then set for all other samples corresponding to the same case. As DNA degradation with time potentially competes with the detection of rare alleles, we only considered single, mutant signal-positive droplets to be positive events, as described in Wong *et al.* [13].

6 Targeted deep amplicon sequencing data analysis

6.1 Selection of positions for deep amplicon sequencing

For all cases, we selected at least 192 predicted mutations to be taken forward for targeted deep amplicon sequencing. These mutations included all non-synonymous (**S18 Fig b**) and synonymous coding sSNVs (**S18 Fig c**) as well as coding sIndels. As this sum fell typically short of 192, we backfilled the list of positions for deep amplicon sequencing to a total of 192 by also including non-coding sSNVs that were proportionally selected from mutational clusters generated from each patient's sSNV VAF (**S18 Fig d**). These clusters are the same clusters identified through the VBBMM used for tumor content estimation (**S18 Fig a**).

6.2 Primer design, library construction, and alignment

Primers were designed using an in-house automated primer design pipeline using the following input parameters for Primer3: [14, 15] primer size 18-26 (optimum 22), primer Tm 57-63 (optimum 59), primer GC% 30-70% (optimum 50%), product size ranges 150-180 and 140-200. Primers were verified to amplify only a single PCR product using the in-silico PCR tool from the UCSC Genome Browser [16]. Forward primers were tagged with 5′- CGCTCTTCCGATCTCTG-3′ and reverse primers with 5′- TGCTCTTCCGATCTGAC-3′. Primer sequences are available on request. PCR was performed in 192 uniplex reactions per normal and tumor sample using 2ng of genomic DNA as input and 0.1 μL (0.2 units) Q5 High-Fidelity DNA Polymerase (NEB) in a 10 μL reaction. PCR conditions were as follows: 98C for 3 minutes, (98C for 80 seconds, 64C for 30 seconds, 72C for 30 seconds)x35, 72C for 2 minutes. Amplicons were pooled per sample and subjected to a second round of PCR using primer pairs containing a single 5 nucleotide index within the reverse primer. Amplicon pools from the second round of PCR were then pooled to a maximum of 20 samples per pool and sequenced on an Illumina MiSeq instrument using 300v2 kits and generating 150bp paired end reads. The BWA aligner (v0.7.5a) was used to align the deep amplicon sequencing reads against the GRCh37 reference genome. Bioinformatics details pertaining to somatic single nucleotide variant validation, clonal dynamics including phylogenetic analysis, prevalence estimation and neutral evolution modeling are presented in the supplementary materials.

6.3 Somatic single nucleotide variant validation

Reads were fitered out if they either 1) aligned >10 base pairs away from an amplicon's start or end position, 2) had >5 mismatched bases, or 3) mapping quality <30. Read counts supporting the reference and variant

base are then extracted for each predicted position from WGS. Only reads that had a base quality ≥ 30 are considered in the counting. After filtering, the mean $\pm$ standard deviation of targeted positions was 10733.37 ± 9025.205 for each sample.

For each targeted position, we calculated the background error rate by interrogating 30 base pairs up- and downstream of the targeted position by calculating the allelic ratio of the most frequent base. The mean allelic ratio of these positions was considered the background error rate after ignoring germline and somatic mutation positions. We next used a binomial exact test to test if the predicted variant allele was present using a p-value of threshold of < 0.000001. A position was considered somatic if the variant allele was present in tumor and absent in matching normal.

In some normal samples, there was evidence of contaminating tumor DNA leading to the presence of the variant allele in the matching normal. To deal with these situations, we performed a one-tailed fisher exact test on the tumor and normal read counts testing if the allelic ratio was higher in the tumor. A significance level of < 0.05 was set to specify if a position was somatic. The mean $\pm$ standard deviation validation rate (precision) was $96.3\% \pm 5.4\%$.

7 Clonal dynamics analysis

For clonal dynamics analysis, we chose to use sSNVs as genetic markers for clonal lineage tracking. There were 2 major reasons for this: 1) the mutational burden of sSNVs is far higher (7133.29 ± 3107.02) than sIndels (512.63 ± 296.67) giving us an adequate number of genetic markers for clonal lineage tracking, 2) using sSNV as a genetic marker for clonal lineage tracking is much less technically challenging than using other genetic markers (e.g. indels). Specifically, the first step in the process of clonal analysis requires the ascertainment of accurate VAFs. This in turn requires accurate alignment of indels which is known to be a difficult problem in the field of sequence aligners. As a result, indel VAF measurements are generally more susceptible to technical noise which in turn affects cellular prevalence, clonal prevalence and ultimately the clonal lineage tracking.

7.1 Estimation of mutational cellular prevalences

For each TFL and PFL patient, we inferred the mutational cellular prevalence of each validated sSNV (aka. proportion of cancer cells with sSNV) for both the T1 and T2 sample. Specifically, any mutation that was validated in a T1 and/or T2 sample ("Somatic single nucleotide variant validation" in the Supplemental

Appendix; **S19 Fig a-b**) was used as an input into PyClone (v0.12.7). For NPFL patients, only the T1 sample was considered. PyClone requires for each sSNV: 1) read count of the variant allele, 2) read count for the reference allele, and 3) major and minor copy number. For 1) and 2), these data were retrieved from the targeted deep amplicon sequencing ("Targeted deep amplicon sequencing data analysis" in the Supplemental Appendix). The major and minor copy number data of each feature was taken from the TITAN segments ("Somatic copy number alteration prediction" in the Supplemental Appendix). In addition to these inputs for each sSNV, the tumor content estimated from the sSNV data ("Tumor content estimation" in the Supplemental Appendix) was also used. Any sSNV without matching copy number data or in a homozygous deleted region is not considered for PyClone analysis.

The PyClone "build_mutations_file" with the parameters "–var_prior parental_copy_number" was used to construct sample-centric mutation yaml files. Patient configuration yaml files were constructed manually with sample-specific tumor content estimations and "error_rate = 0.001" along with the following parameters:

```
density: pyclone_beta_binomial
num_iters: 100000
base_measure_param:
  alpha: 1.0
  beta: 1.0
concentration:
  value: 1.0
  prior:
    shape: 1.0
    rate: 0.001
beta_binomial_precision_params:
  value: 1000.0
  prior:
    shape: 1.0
    rate: 0.0001
  proposal:
    precision: 0.01
```

These yaml files were then used as input into the “analyze” function of PyClone with the parameters “–seed 1000”. The “build_table” function with the parameter “–burnin 50000” was then used to retrieve the estimated mutational cellular prevalence of each sSNV (**S19 Fig c**). As some sSNVs had large uncertainty in their estimated mutational cellular prevalence, we performed a credible interval analysis using the “CI_filter.py” script from PostPy (v0.1) using the parameters “-b 50000 -i 90 -r 0”. Any sSNVs with a 90% credible interval larger than 0.3 in any sample of a patient were filtered out. In addition, positions in the IGH location with low coverage were also filtered out. PyClone was then re-run without the filtered sSNVs.

7.2 Estimation of clonal prevalence and phylogenies

sSNVs with similar mutational cellular prevalences were clustered by PyClone. We next calculated each cluster's mean cellular prevalence by taking the mean of cellular prevalence of all sSNVs in the cluster (**S19 Fig d**). After filtering out singleton clusters (i.e. clusters with only a single sSNV) and clusters with subclonal subclonal copy number changes, cluster means were then used as input into Citup [17] (v0.2) to estimate clonal prevalences and phylogenies (**S19 Fig d-e**).

7.3 Filtering of PyClone clusters for clonal phylogeny construction

The following filters were applied to PyClone clusters to remove clusters before input into Citup: 1) all singleton PyClone clusters (i.e. clusters with only a single sSNV), 2) clusters with subclonal copy number changes as these result in inaccurate VAF and mutational cellular prevalence estimations, 3) clusters that violated phylogenetic construction rules. Specific details on each filtered patient cluster are described below.

For FL1005, cluster 1 has a subclonal deletion that results in the variant alleles being deleted in subset of the tumor cells. This results in a decrease in VAF and mutational cellular prevalence resulting in a different cluster. Similarly, cluster 2 has a subclonal deletion that results in the reference alleles being deleted in subset of the tumor cells. This results in an increase in VAF and mutational cellular prevalence resulting in a different cluster. The sSNVs in these two clusters should belong in the ancestor cluster 3.

For FL2007, cluster 1 has subclonal amplifications of the reference alleles resulting in a decrease in VAF and mutational cellular prevalence resulting in a different cluster. The sSNVs in this cluster should belong in the ancestor cluster 3.

For FL2008, cluster 5 has two sSNVs chr18:75515528 and chr6:2606119 both of which have a subclonal copy number change. Specifically, chr18:75515528 has a subclonal deletion that deletes the variant allele (T). While chr6:2606119 has a subclonal copy number gain that results in the reference allele (C) being gained.

The sSNVs in this cluster should belong in the ancestor cluster 6. Cluster 4 violates the additive rule as the sum of the T2 mutational cellular prevalences of clusters 3 and 10 are greater than cluster 4.

8 Neutral evolution modeling

Genetic drift trajectory modeling was performed using the Wright-Fisher model [18]. Under this model, the following assumptions are made: 1) generations do not overlap, and 2) each copy of an allele in a generation is independently drawn from the previous generation at random, and 3) the number of cells does not change between generations. Simulations were initialized with 10000 diploid cells and a starting mutant VAF of 0.01 for TFL and 0.5 for PFL patients. Each simulation was run for 10000 generations and a total of 1000 simulations were run. K-means clustering, ranging from 2 to 10 clusters (k), was used to cluster together simulations that exhibit similar genetic drift trajectories. To decide on the optimal number of clusters, the total within sum of squares vs. k-clusters was plotted and the optimal number of cluster was selected based on the elbow of the curve. This resulted in 5 and 4 optimal clusters for TFL and PFL patients respectively.

After identifying the cluster with the genetic drift trajectory most similar to the observed patterns in TFL patients, the proportion of simulations of that trajectory was used as the background trajectory rate. This rate was then used as input into the binomial exact test to quantify the probability of 13 out of 15 TFL patients following the observed patterns.

9 Capture-based targeted sequencing

9.1 Capture panel and library construction

We selected 86 genes (**S10 Table**) for capture-based targeted sequencing, based on the following 6 criteria: 1) recurrence in FL of >5%, [19,20], 2) recurence in DLBCL >5% (our own data) or reported to be consistently mutated in Burkitt lymphoma across studies, [21–23], 3) genes significantly mutated (q <0.05) in the compiled dataset from our study and others [24,25] applying the MutSigCV algorithm [26] (v1.4), 4) T1 gene mutations associated with early transformation/progression (<5 years) versus no progression (for at least 5 years) based on our data and external cohorts [24,25], 5) genes that were found to be T2-specific in at least 3 cases from our and external cohorts, [24, 25] and 6. *IL4R*, *PTPN1*, *NOTCH2* and *RFX5*. Furthermore, we selected 20 genes, 12 of which overlapped with the 86 above-mentioned genes, to assess mutations in areas that are targets of somatic hypermutation (**S11 Table**). Libraries were constructed from either 500ng of fresh-frozen

genomic DNA or 200ng of FFPET-derived genomic DNA, and captured using custom SureSelectXT2 baits (Agilent). Captured libraries were pooled to a maximum of 46 libraries per pool and each pool was sequenced on one Illumina HiSeq lane, generating 125bp indexed reads (V4 chemistry). In total, the capture space was $452,129$ base pairs. Analysis details of capture-based sequencing are provided in the supplementary materials.

9.2 Alignment and sequencing statistics

Paired end reads were aligned using BWA (v0.5.7) against the GRCh37 genome (`http://www.bcgsc.ca/downloads/genomes/9606/hg19/1000genomes/bwa_ind/genome/GRCh37-lite.fa`). PCR duplicates were marked using Picard tools (v1.126) using MarkDuplicates and sequencing statistics were collected using CalculateHsMetrics. Samples with less than 50x mean target coverage (only FFPET, $n = 22$) were excluded from all subsequent analysis. For samples available for analysis, the mean $\pm$ standard deviation coverage was 1046.96 ± 229.88 for fresh-frozen samples (tumor and normals) and 192.56 ± 120.49 for FFPET samples.

Full sequencing statistics can be found in **S12 Table**.

9.3 Somatic single point mutations analysis

MutationSeq [5] (v4.3.8) was used to predict sSNVs in deep sequencing mode in single sample and in tumor-normal pair mode (when applicable). The models “model_deep_single_v0.1.npz” and “model_deep_v0.2.npz” were used for single and paired mode respectively. sSNV predictions were filtered as follows:

- based on mutation probability and coverage:
  - fresh-frozen samples: mutation probabibilty > 0.8 and coverage ≥ 100, or mutation probability > 0.9 and coverage < 100;
  - FFPET samples: predictions were filtered as follows: mutation probability > 0.7 and coverage ≥ 50, or mutation probability > 0.9 and coverage < 50.
- filtering out of putative SNPs:
  - for cases with available germline DNA sequenced ($n = 80$), variants were filtered out if they were present in the germline samples;
  - for cases without available germline DNA sequenced, SNVs were filtered out if they were found in at least 2 normal (unrelated) samples or in dbSNP (v147);

  - for 8 cases without germline DNA sequenced by targeted capture sequencing, SNPs were filtered out using matching germline whole-genome sequencing libraries;
  - variants that occurred more than 4 times and had a mean variant allele frequency > 0.45 and < 0.55, with a standard deviation of < 0.1 were filtered out.

- any variant that occurred within a distance of < 10 base pairs of an indel was filtered out.
- putative artifacts were filtered out, defined as as variants that occurred more than 5 times in either fresh-frozen or FFPET samples, with a mean variant allele frequency of < 0.15 or > 0.9; or variants that occured in more than 10% of either fresh-frozen of FFPET samples.

sSNVs were annotated with SnpEFF and if multiple effects were called for a single position, the effect with the putatively greatest impact was chosen as described above for SNV calling in our WGS cases.

For samples with matching germline, indels were called using Strelka (v1.0.13) and the output was not filtered. For samples without matching germline, indels were called using VarScan and germline variants were filtered out using dbSNP (v137) and the 1000 genomes. Artifacts were filtered out in an analogous fashion to sSNVs.

9.4 Copy number analysis

Copy number analysis was performed using CNVkit [27] (v0.7.11) using the workflow depicted in **S20 Fig**. Only the FF samples were usable for this analysis as the fragmentation of FFPET samples resulted in poor quality copy number estimates (data not shown). SNPs were generated using VarScan and restricted to positions found in dbSNP (v137). These SNPs were then used as input to generate parental copy number in CNVkit. Copy number segments were projected onto gene scaffords (Ensembl v72) to generate gene-centric copy number estimates.

9.5 Determination of mutational cellular prevalence

This analysis was restricted to samples from those 32 patients in which fresh-frozen samples had been sequenced from both timepoints by capture-based sequencing and that had not been subjected to WGS. Mutational cellular prevalence was determined per sSNV using PyClone, using the following inputs:

- variant allele frequency from repeat MutationSeq run, providing variant allele frequencies even if the variant had not been called by our SNV prediction pipeline;

- tumor content estimated from clustering of variant allele frequencies across both samples from each patient;
- copy number status determined using CNVkit.

sSNVs were filtered out of samples from both timepoints for a given patient if the standard deviation of the estimate of cellular prevalence was > 0.1 in any of the two samples.

10 Code availability

The software used in this project, along with their version indicated with "v" prefix, is listed below. URL addresses are provided to indicate where to obtain the software.

- BWA [3] (v0.5.7): `http://bio-bwa.sourceforge.net`
- Bcftools [28] (v0.1.18): `http://samtools.sourceforge.net`
- Citup [17] (v0.2): `https://bitbucket.org/dranew/citup`
- CNVkit [27] (v0.7.11): `https://github.com/etal/cnvkit`
- Destruct (v0.2.0): `https://bitbucket.org/dranew/destruct`
- HMMcopy [9] (v0.1.1): `https://bioconductor.org/packages/release/bioc/html/HMMcopy.html`
- MutSigCV [26] (v1.4): `https://www.broadinstitute.org/cancer/cga/mutsig`
- MutationSeq [5] (v4.1.0): `http://compbio.bccrc.ca/software/mutationseq`
- Picard Tools (v1.126): `http://broadinstitute.github.io/picard`
- Samtools [28] (v0.1.18): `http://samtools.sourceforge.net`
- SnpEff [29]: `http://snpeff.sourceforge.net/`
- SnpSift [29]: `http://snpeff.sourceforge.net/`
- Strelka [6] (v1.0.13): `https://sites.google.com/site/strelkasomaticvariantcaller/`
- TITAN [8] (v1.5.7): `https://www.bioconductor.org/packages/release/bioc/html/TitanCNA.html`
- PostPy (v0.1): `http://compbio.bccrc.ca/software/postpy`

- PyClone [7] (v1.0.13): `http://compbio.bccrc.ca/software/pyclone/`
- VBBMM: `https://github.com/tinyheero/vbbmm`

Software parameters are listed in the relevant sections above when they differ from the default values or to add clarity to the generation of results.

References

1. Scott DW, Wright GW, Williams PM, Lih CJ, Walsh W, Jaffe ES, et al. Determining cell-of-origin subtypes of diffuse large B-cell lymphoma using gene expression in formalin-fixed paraffin-embedded tissue. Blood. 2014;123(8):1214–7. doi:10.1182/blood-2013-11-536433.

2. Kridel R, Mottok A, Farinha P, Ben-neriah S, Ennishi D, Zheng Y, et al. Cell of origin of transformed follicular lymphoma. Blood. 2015;126(18):2118–2128. doi:10.1182/blood-2015-06-649905.

3. Li H, Durbin R. Fast and accurate short read alignment with Burrows-Wheeler transform. Bioinformatics. 2009;.

4. Costello M, Pugh TJ, Fennell TJ, Stewart C, Lichtenstein L, Meldrim JC, et al. Discovery and characterization of artifactual mutations in deep coverage targeted capture sequencing data due to oxidative DNA damage during sample preparation. Nucleic Acids Res. 2013;41(6):e67. doi:10.1093/nar/gks1443.

5. Ding JJ, Bashashati AA, Roth AA, Oloumi AA, Tse KK, Zeng TT, et al. Feature-based classifiers for somatic mutation detection in tumour-normal paired sequencing data. Bioinformatics. 2012;28(2):167–175.

6. Saunders CT, Wong WSW, Swamy S, Becq J, Murray LJ, Cheetham RK. Strelka: accurate somatic small-variant calling from sequenced tumor-normal sample pairs. Bioinformatics. 2012;28(14):1811–1817.

7. Roth A, Khattra J, Yap D, Wan A, Laks E, Biele J, et al. PyClone: statistical inference of clonal population structure in cancer. Nat Methods. 2014;11(4):396–398.

8. Ha G, Roth A, Khattra J, Ho J, Yap D, Prentice LM, et al. TITAN: inference of copy number architectures in clonal cell populations from tumor whole-genome sequence data. Genome Res. 2014;24(11):1881–1893.

9. Lai D, Ha G, Shah S. HMMcopy: Copy number prediction with correction for GC and mappability bias for HTS data. Bioconductor. 2012;.

10. Green MR, Monti S, Rodig SJ, Juszczynski P, Currie T, O'Donnell E, et al. Integrative analysis reveals selective 9p24.1 amplification, increased PD-1 ligand expression, and further induction via JAK2 in nodular sclerosing Hodgkin lymphoma and primary mediastinal large B-cell lymphoma. Blood. 2010;116(17):3268–3277.

11. Curtis C, Shah SP, Chin SF, Turashvili G, Rueda OM, Dunning MJ, et al. The genomic and transcriptomic architecture of 2,000 breast tumours reveals novel subgroups. Nature. 2012;486(7403):346–352.

12. McPherson AW, Wu C, Wyatt A, Shah SP, Collins C, Sahinalp SC. nFuse: Discovery of complex genomic rearrangements in cancer using high-throughput sequencing. Genome Res. 2012;.

13. Wong TN, Ramsingh G, Young AL, Miller Ca, Touma W, Welch JS, et al. Role of TP53 mutations in the origin and evolution of therapy-related acute myeloid leukaemia. Nature. 2014;doi:10.1038/nature13968.

14. Koressaar T, Remm M. Enhancements and modifications of primer design program Primer3. Bioinformatics. 2007;23(10):1289–1291. doi:10.1093/bioinformatics/btm091.

15. Untergasser A, Cutcutache I, Koressaar T, Ye J, Faircloth BC, Remm M, et al. Primer3-new capabilities and interfaces. Nucleic Acids Res. 2012;40(15):1–12. doi:10.1093/nar/gks596.

16. Kent JW, Sugnet CW, Furey TS, Roskin KM, Pringle TH, Zahler AM, et al. The human genome browser at UCSC. Genome Res. 2002;12(6):996–1006. doi:10.1101/gr.229102. Article published online before print in May 2002.

17. Malikic S, McPherson AW, Donmez N, Sahinalp CS. Clonality inference in multiple tumor samples using phylogeny. Bioinformatics. 2015;31(9):1349–1356.

18. Ewens WJ. Mathematical Population Genetics. vol. 27 of Interdisciplinary Applied Mathematics. New York, NY: Springer New York; 2004.

19. Green MR, Kihira S, Liu CL, Nair RV, Salari R, Gentles AJ, et al. Mutations in early follicular lymphoma progenitors are associated with suppressed antigen presentation. Proc Natl Acad Sci U S A. 2015; p. 201501199. doi:10.1073/pnas.1501199112.

20. Pastore A, Jurinovic V, Kridel R, Hoster E, Staiger AM, Szczepanowski M, et al. Integration of gene mutations in risk prognostication for patients receiving first-line immunochemotherapy for follicular lymphoma: a retrospective analysis of a prospective clinical trial and validation in a population-based registry. Lancet Oncol. 2015;2045(15):1–12. doi:10.1016/S1470-2045(15)00169-2.

21. Schmitz R, Young RM, Ceribelli M, Jhavar S, Xiao W, Zhang M, et al. Burkitt lymphoma pathogenesis and therapeutic targets from structural and functional genomics. Nature. 2012;490(7418):116–20. doi:10.1038/nature11378.

22. Love C, Sun Z, Jima D, Li G, Zhang J, Miles R, et al. The genetic landscape of mutations in Burkitt lymphoma. Nat Genet. 2012;44(12):1321–5. doi:10.1038/ng.2468.

23. Richter J, Schlesner M, Hoffmann S, Kreuz M, Leich E, Burkhardt B, et al. Recurrent mutation of the ID3 gene in Burkitt lymphoma identified by integrated genome, exome and transcriptome sequencing. Nat Genet. 2012;44(12):1316–20. doi:10.1038/ng.2469.

24. Pasqualucci L, Khiabanian H, Fangazio M, Vasishtha M, Messina M, Holmes A, et al. Genetics of Follicular Lymphoma Transformation. Cell Rep. 2014;6(1):130–140. doi:10.1016/j.celrep.2013.12.027.

25. Okosun J, Bödör C, Wang J, Araf S, Yang CY, Pan C, et al. Integrated genomic analysis identifies recurrent mutations and evolution patterns driving the initiation and progression of follicular lymphoma. Nat Genet. 2014;46(2):176–81. doi:10.1038/ng.2856.

26. Lawrence MS, Stojanov P, Polak P, Kryukov GV, Cibulskis K, Sivachenko A, et al. Mutational heterogeneity in cancer and the search for new cancer-associated genes. Nature. 2013;499(7457):214–8. doi:10.1038/nature12213.

27. Talevich E, Shain AH, Botton T, Bastian BC. CNVkit: Genome-Wide Copy Number Detection and Visualization from Targeted DNA Sequencing. PLOS Computational Biology. 2016;12(4):e1004873. doi:10.1371/journal.pcbi.1004873.

28. Li H, Handsaker B, Wysoker A, Fennell T, Ruan J, Homer N, et al. The Sequence Alignment/Map format and SAMtools. Bioinformatics. 2009;25(16):2078–2079.

29. Cingolani P, Platts A, Wang LL, Coon M, Nguyen T, Wang L, et al. A program for annotating and predicting the effects of single nucleotide polymorphisms, SnpEff: SNPs in the genome of Drosophila melanogaster strain w1118; iso-2; iso-3. Fly. 2012;6(2):80–92.
