# Supplementary material for: Histological Transformation and Progression in Follicular Lymphoma: A Clonal Evolution Study
Source: PLoS Med. 2016 Dec 13;13(12):e1002197. doi: 10.1371/journal.pmed.1002197 (PMC5154502; doi:10.1371/journal.pmed.1002197)
Supplement: S1 Fig — (PDF) [file pmed.1002197.s002.pdf]

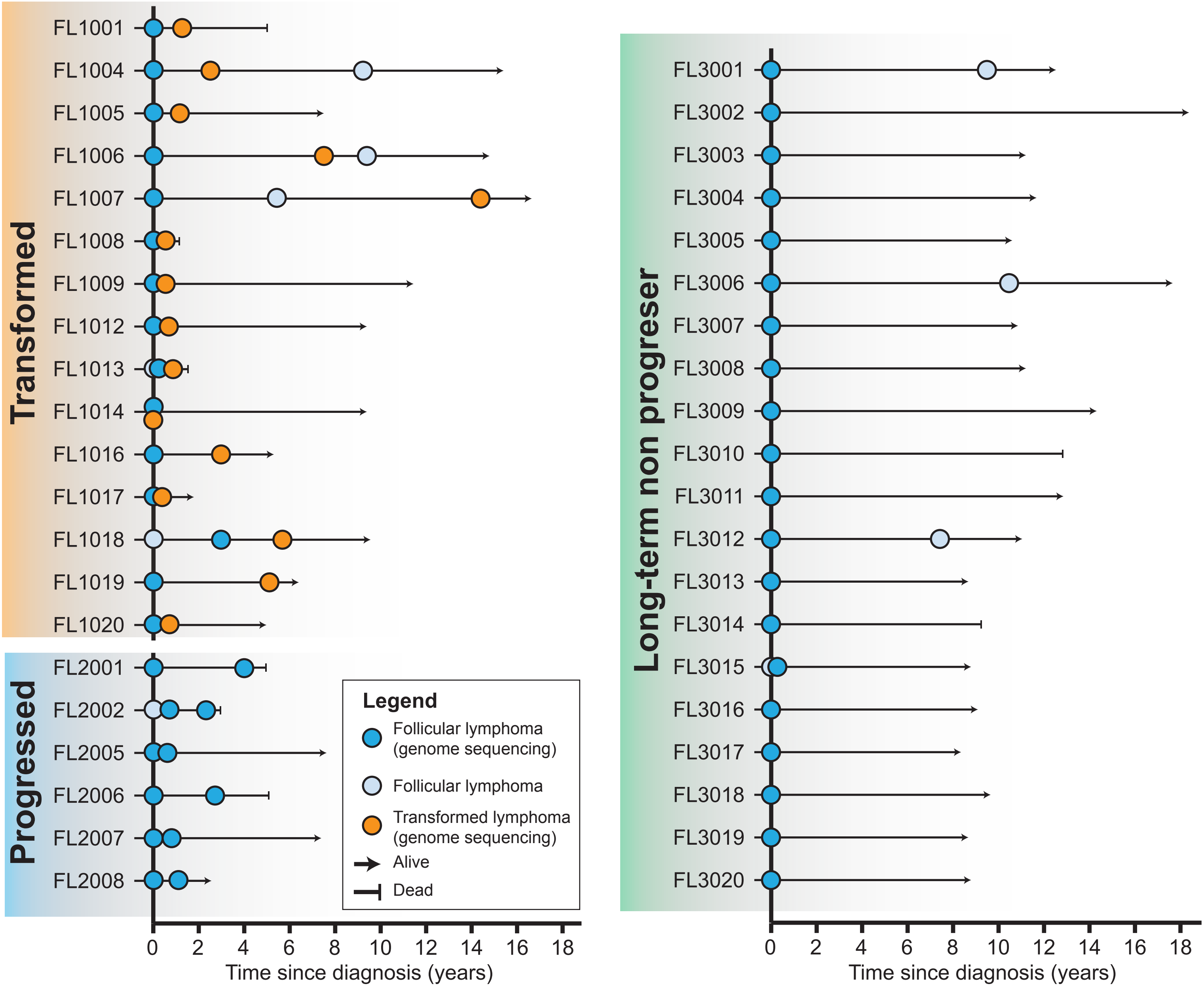

Transformed
FL1001
FL1004
FL1005
FL1006
FL1007
FL1008
FL1009
FL1012
FL1013
FL1014
FL1016
FL1017
FL1018
FL1019
FL1020
Progressed
FL2001
FL2002
FL2005
FL2006
FL2007
FL2008
Legend
Follicular lymphoma (genome sequencing)
Follicular lymphoma
Transformed lymphoma (genome sequencing)
Alive
Dead
0 2 4 6 8 10 12 14 16 18
Time since diagnosis (years)
Long-term non progreser
FL3001
FL3002
FL3003
FL3004
FL3005
FL3006
FL3007
FL3008
FL3009
FL3010
FL3011
FL3012
FL3013
FL3014
FL3015
FL3016
FL3017
FL3018
FL3019
FL3020
0 2 4 6 8 10 12 14 16 18
Time since diagnosis (years)
