# Supplementary figures and images for: Histological Transformation and Progression in Follicular Lymphoma: A Clonal Evolution Study
Source: PLoS Med. 2016 Dec 13;13(12):e1002197. doi: 10.1371/journal.pmed.1002197 (PMC5154502; doi:10.1371/journal.pmed.1002197)

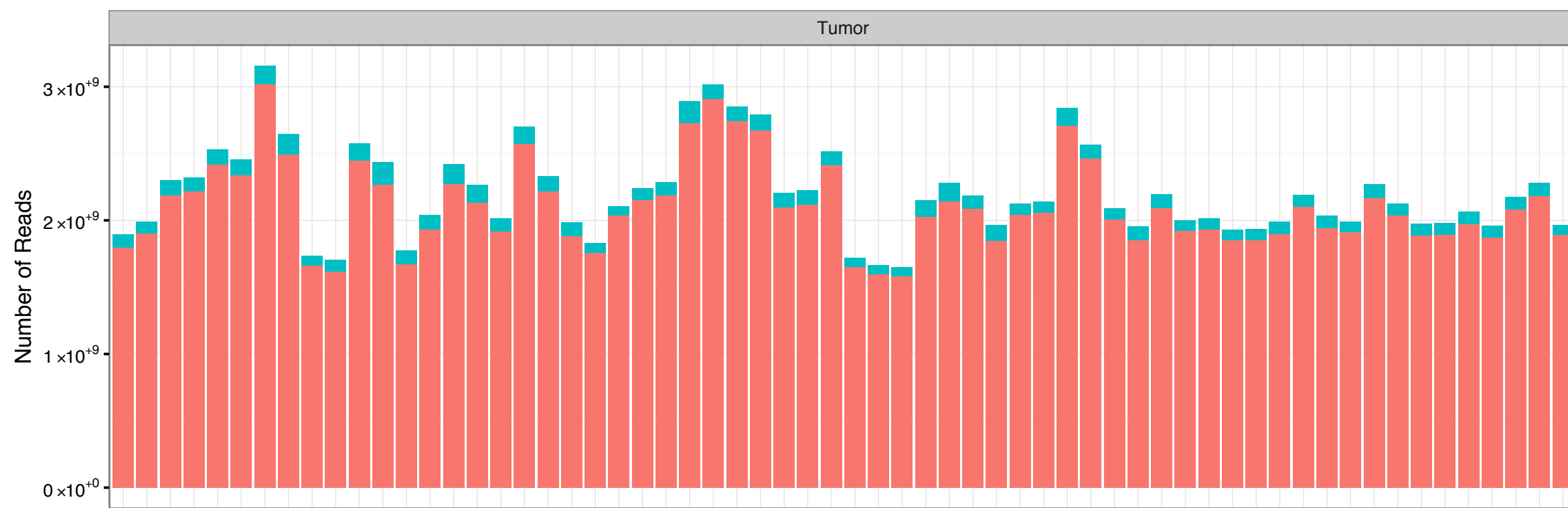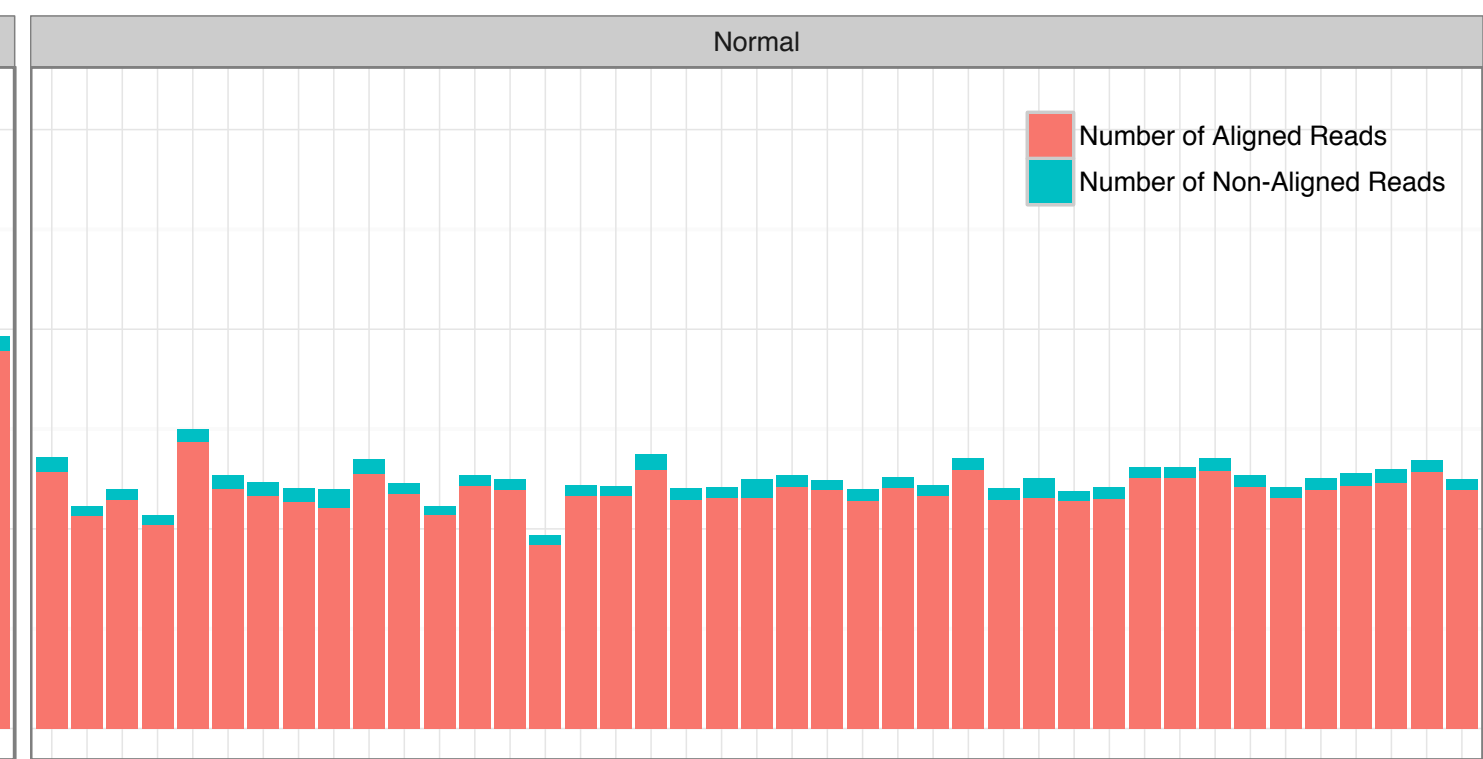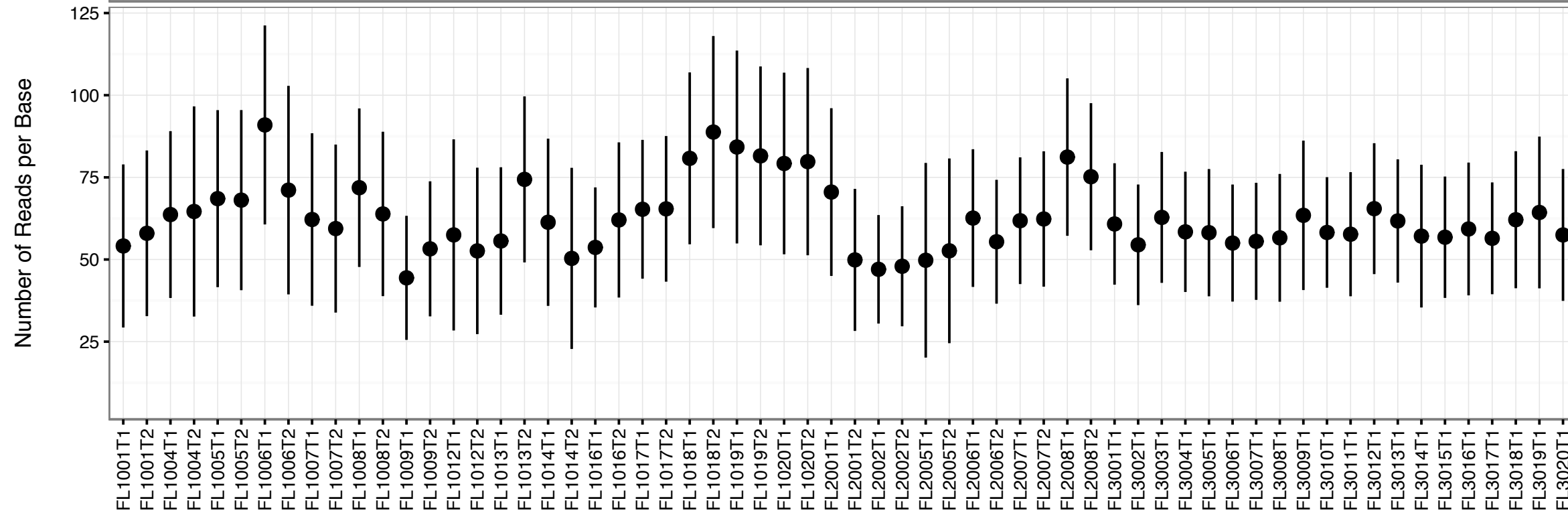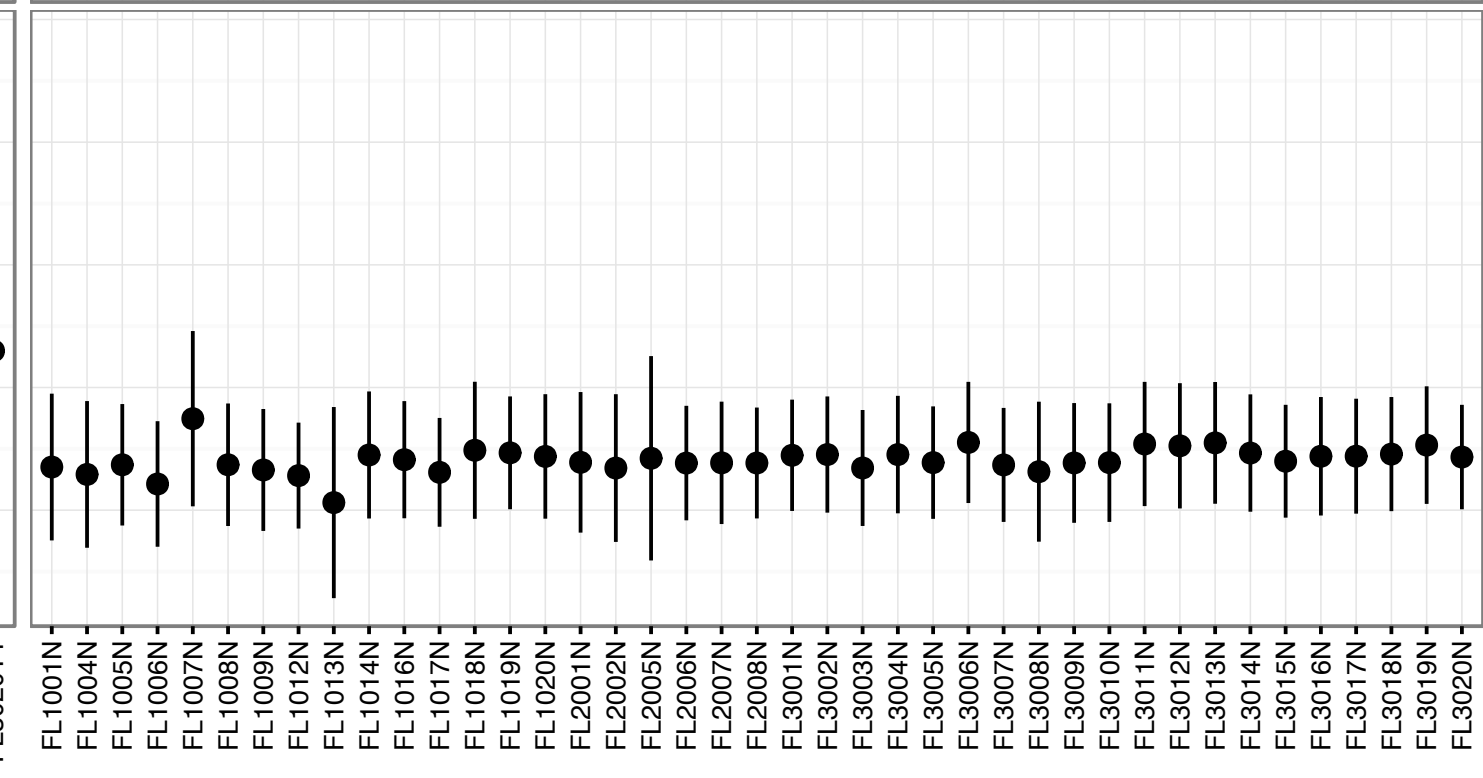

Supplement: S2 Fig — (PDF) [file pmed.1002197.s003.pdf]

Difference in T2 and T1 Mutation Load

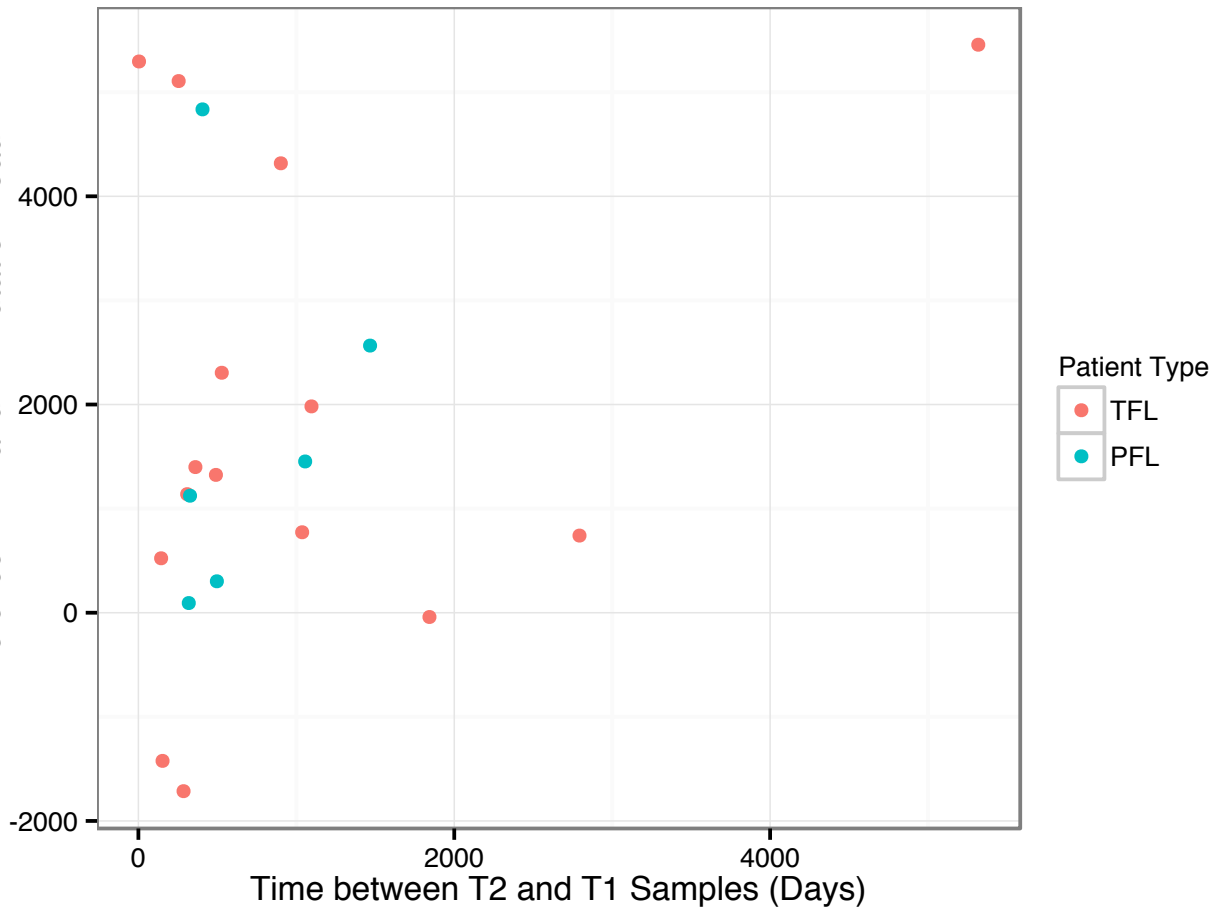

Supplement: S3 Fig — (PDF) [file pmed.1002197.s004.pdf]

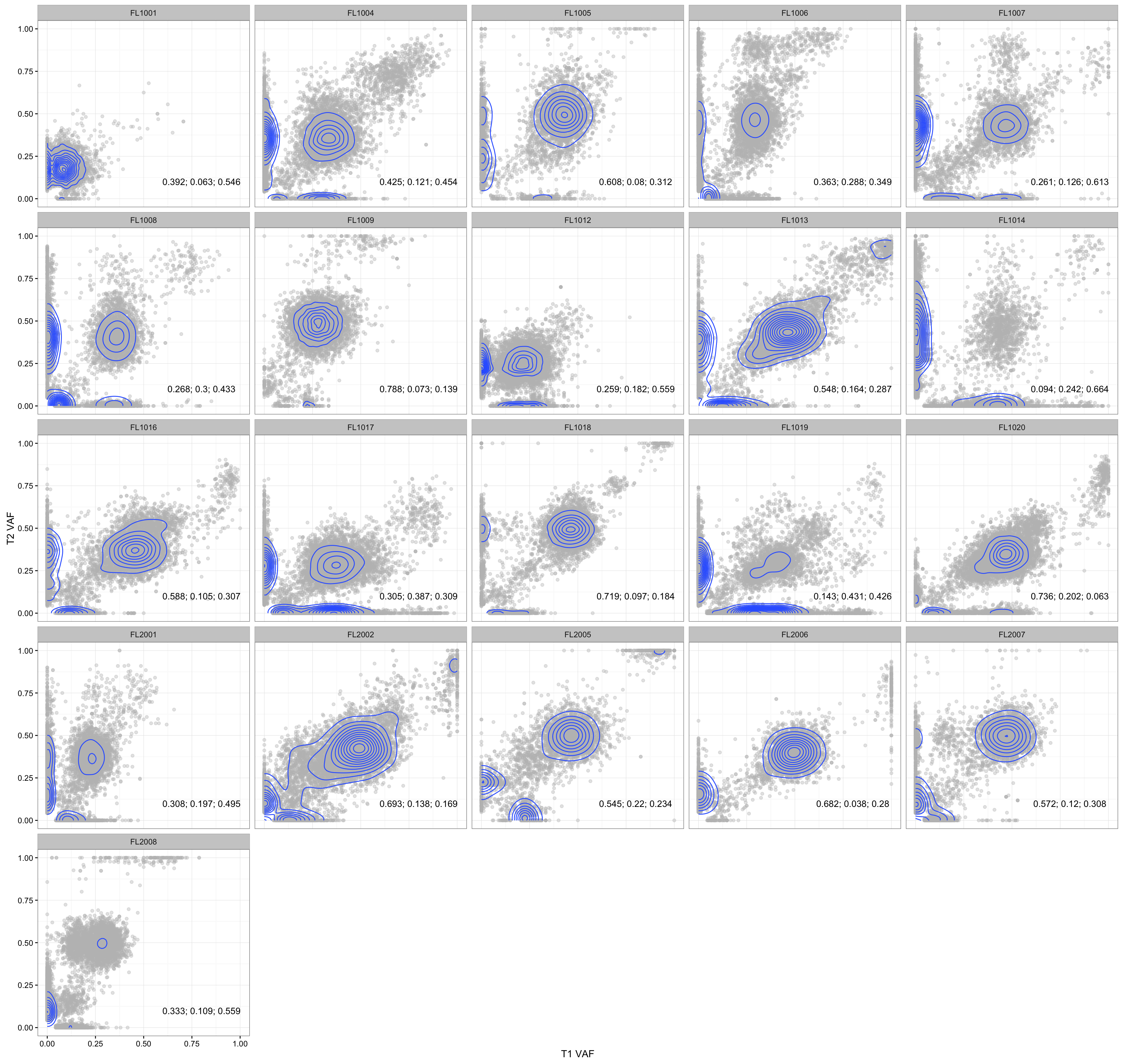

Supplement: S4 Fig — WGS T2 versus T1 variant allele frequencies for TFL and PFL patients. The resulting fraction of predicted time point-specific mutations is listed in the bottom right-hand corner of each patient plot (Shared; T1; T2). (PNG) [file pmed.1002197.s005.png]

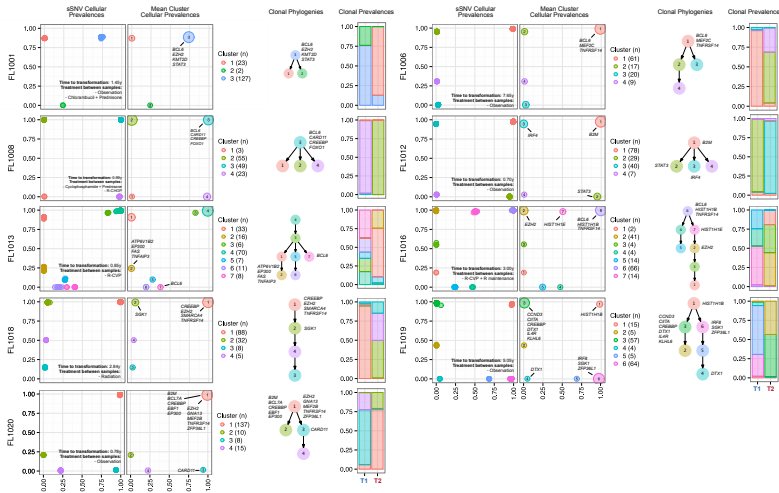

Supplement: S5 Fig — From mutation cellular prevalences to clonal phylogenies and clonal prevalences for TFL patients not represented in Fig 3. For each given patient, the leftmost plot shows the PyClone cellular prevalence of each validated sSNV (i.e., somatic in the T1 and/or T2 sample) at T1 (x-axes) and T2 (y-axes), with each mutation colored by the cluster it belongs to. The next plot to the right represents the cluster cellular prevalence (mean cellular prevalence of all mutations in the cluster), with the size of the circle representing the number of mutations in the cluster. This is followed by a clonal phylogeny and then a stacked bar plot representing the clonal prevalence of each clone in the T1 and T2 sample. The colors of the clusters have no meaning across patients. The n in parentheses beside the cluster color and number represents the number of sSNVs in that cluster. (PDF) [file pmed.1002197.s006.pdf]

Mutation cellular prevalence

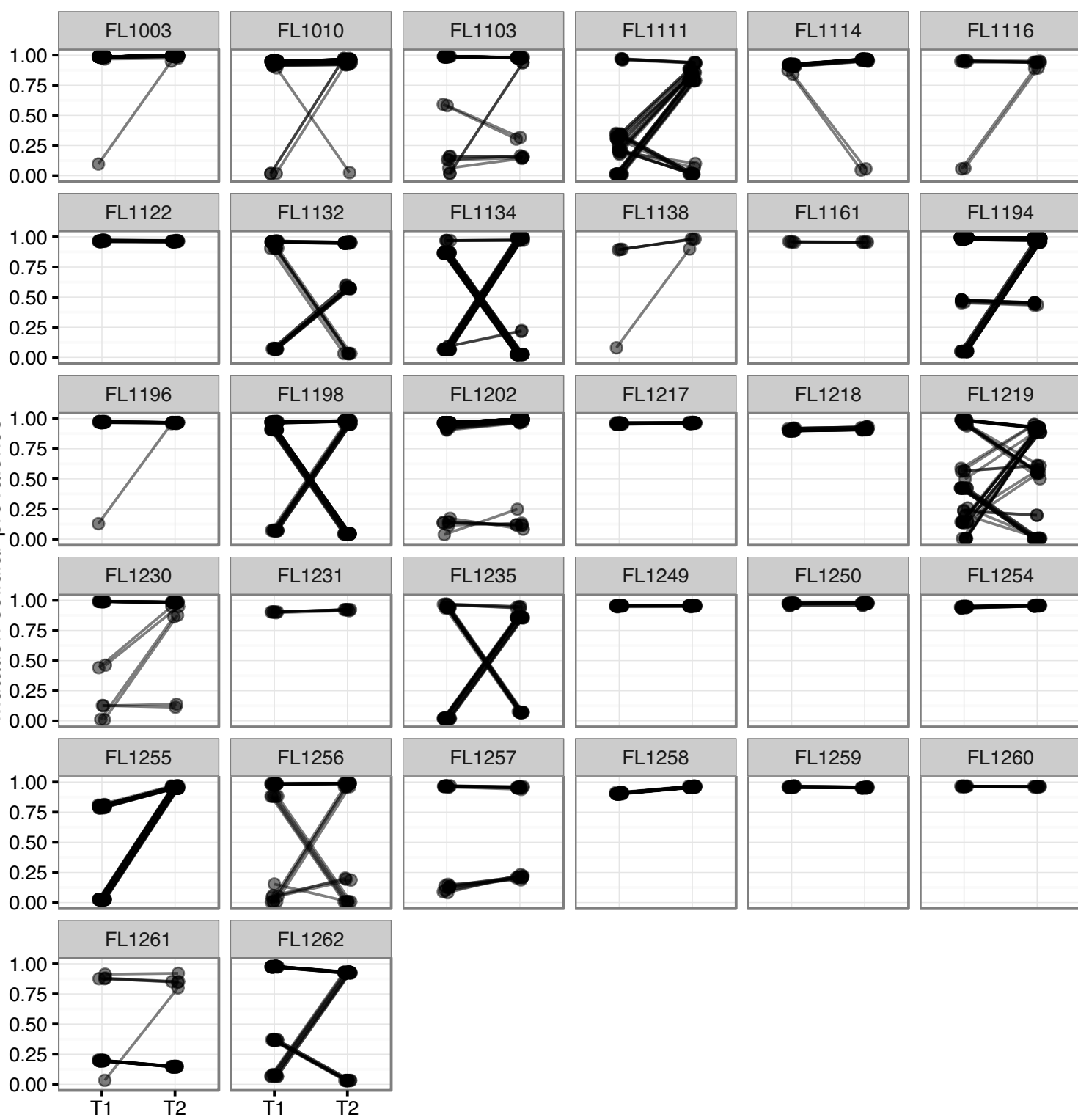

Supplement: S7 Fig — (PDF) [file pmed.1002197.s008.pdf]

proportion

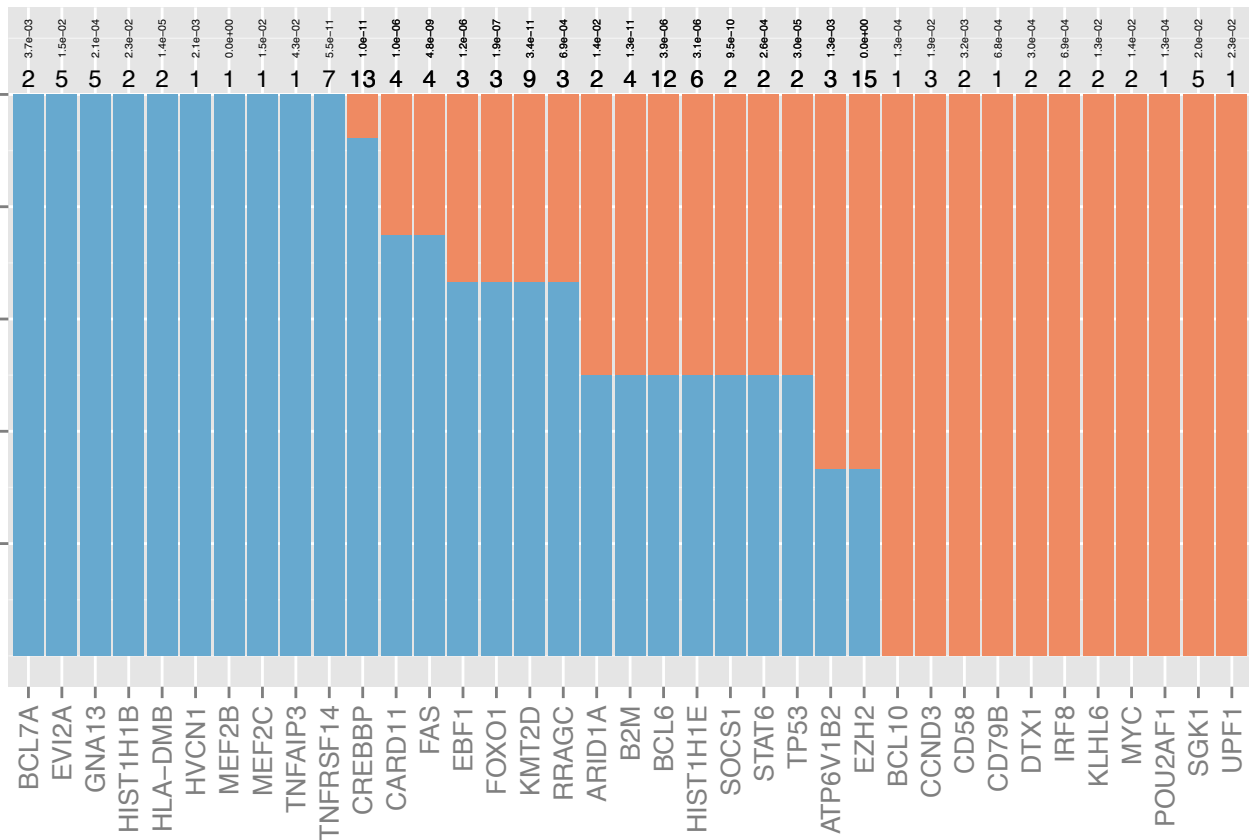

← MutSigCV fdr value

← number of mutations per gene

type

ancestral  
derivative

Supplement: S8 Fig — Proportion of gene mutations that are ancestral or derivative based on clonal analysis of the whole genome sequencing cohort (transformed and progressed cases). Shown are only genes that are significantly mutated based on a MutSigCV q-value < 0.05 in the combined analysis of our data and the data from Okosun et al. [20] and Pasqualucci et al. [21]. (PDF) [file pmed.1002197.s009.pdf]

Mutated Genes per Patient (n)

40

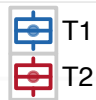

T1

T2

$P=5.75e-05$

T1

Text

T2

Timepoint

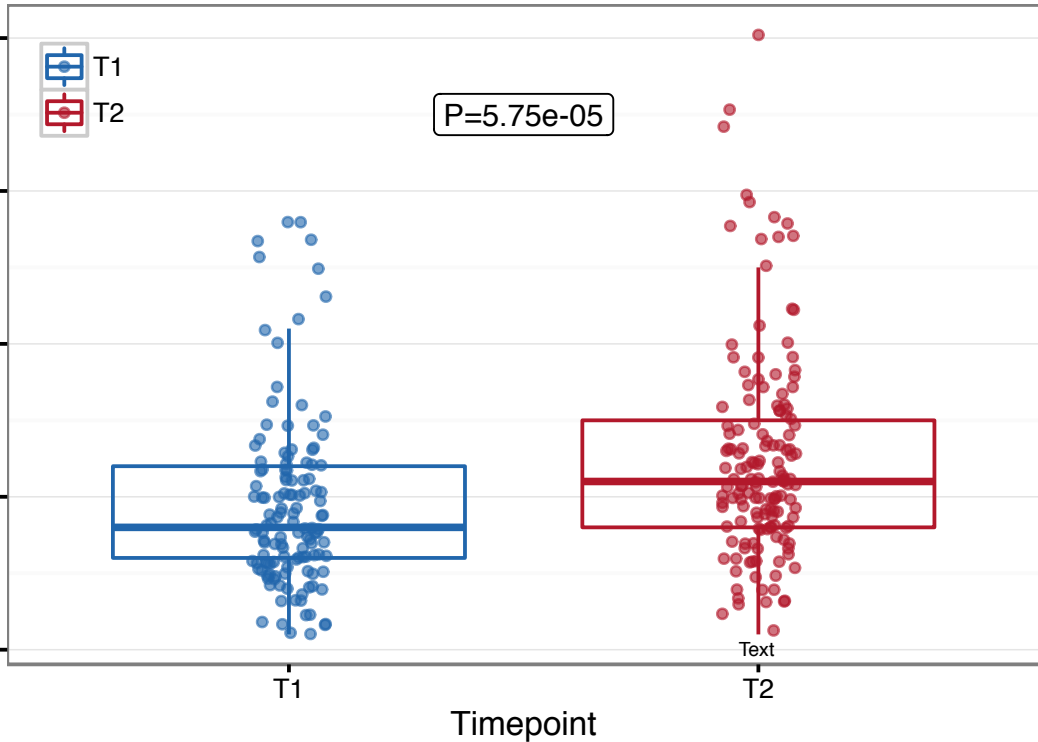

Supplement: S9 Fig — Non-synonymous single nucleotide variants as well as small insertions or deletions were considered in this analysis. (PDF) [file pmed.1002197.s010.pdf]

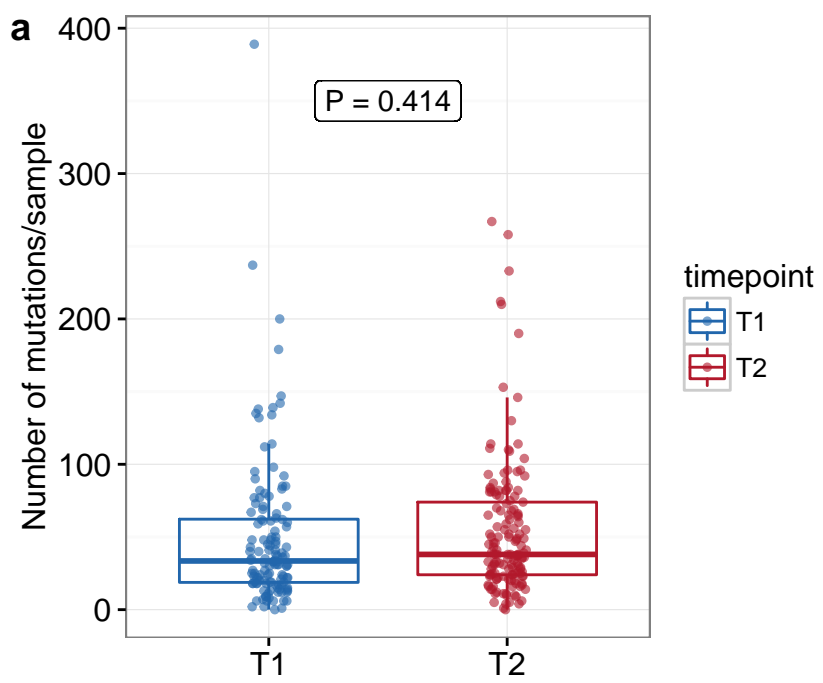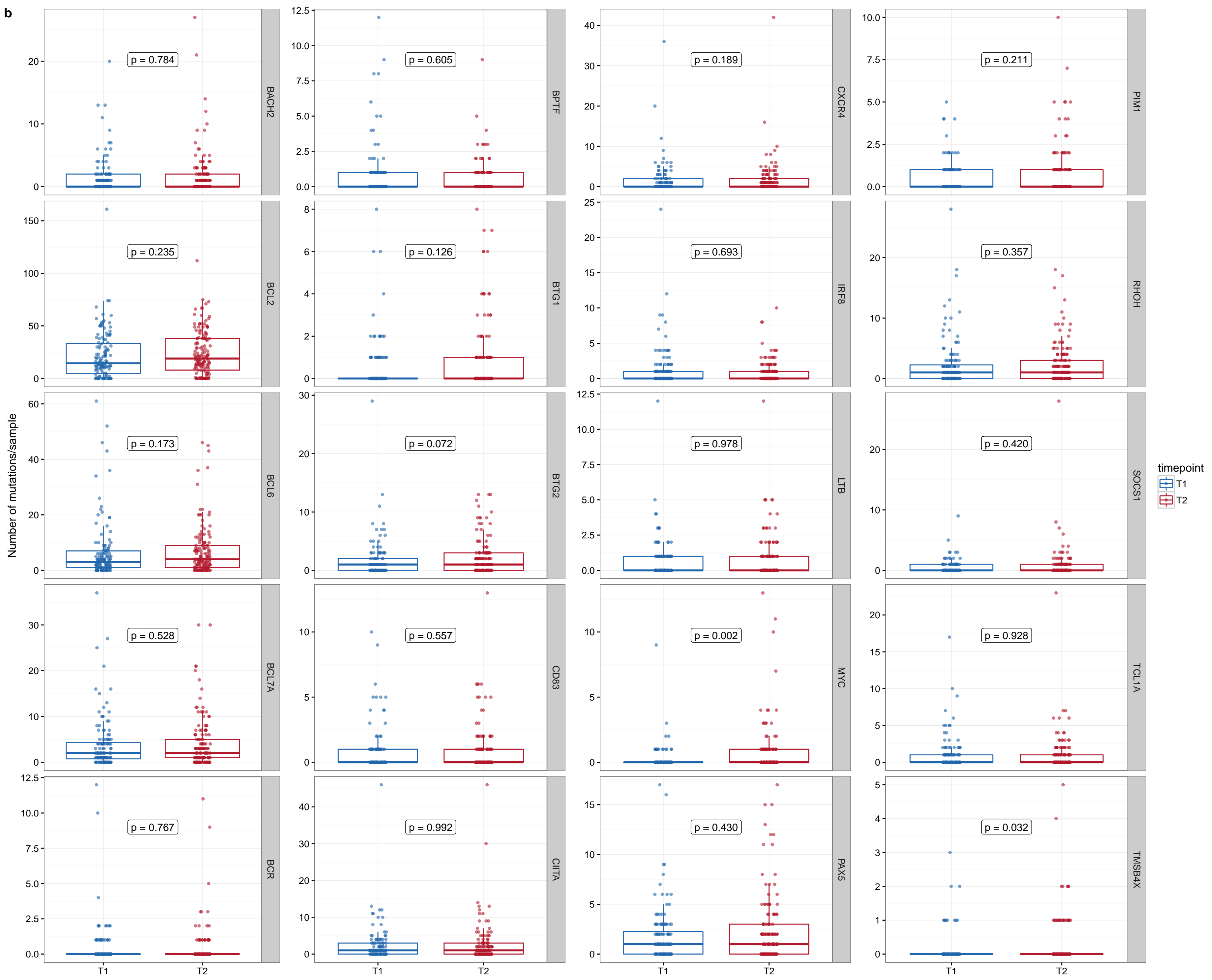

Supplement: S10 Fig — (A) Number of mutations per sample found in 20 genes, by time point (128 FL samples, 149 TFL samples; total number of patients, n = 159). (B) Number of mutations per sample, by gene and by time point. (PDF) [file pmed.1002197.s011.pdf]

### Progression-free survival

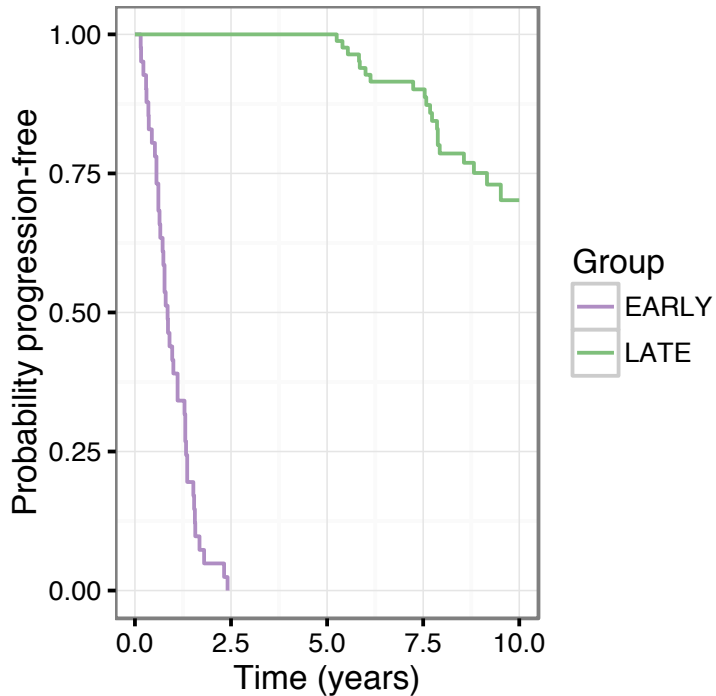

### Overall survival

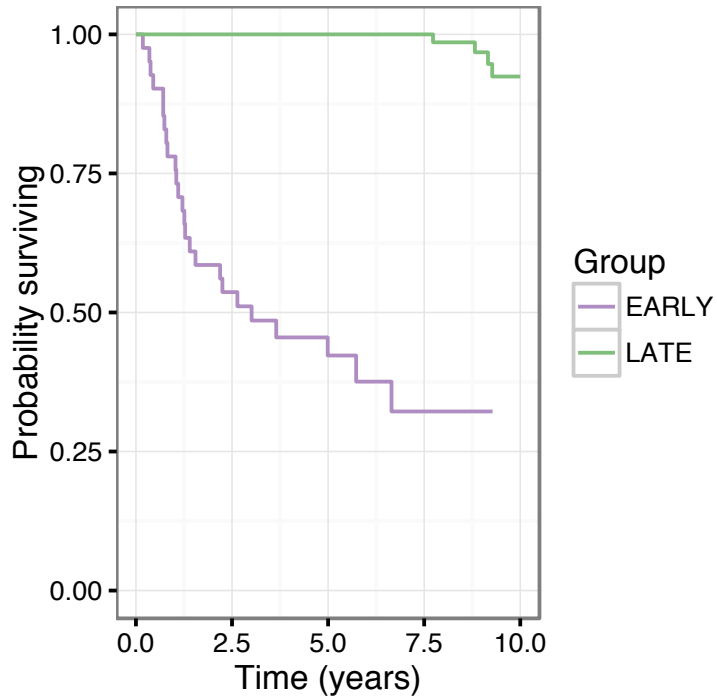

Supplement: S11 Fig — (PDF) [file pmed.1002197.s012.pdf]

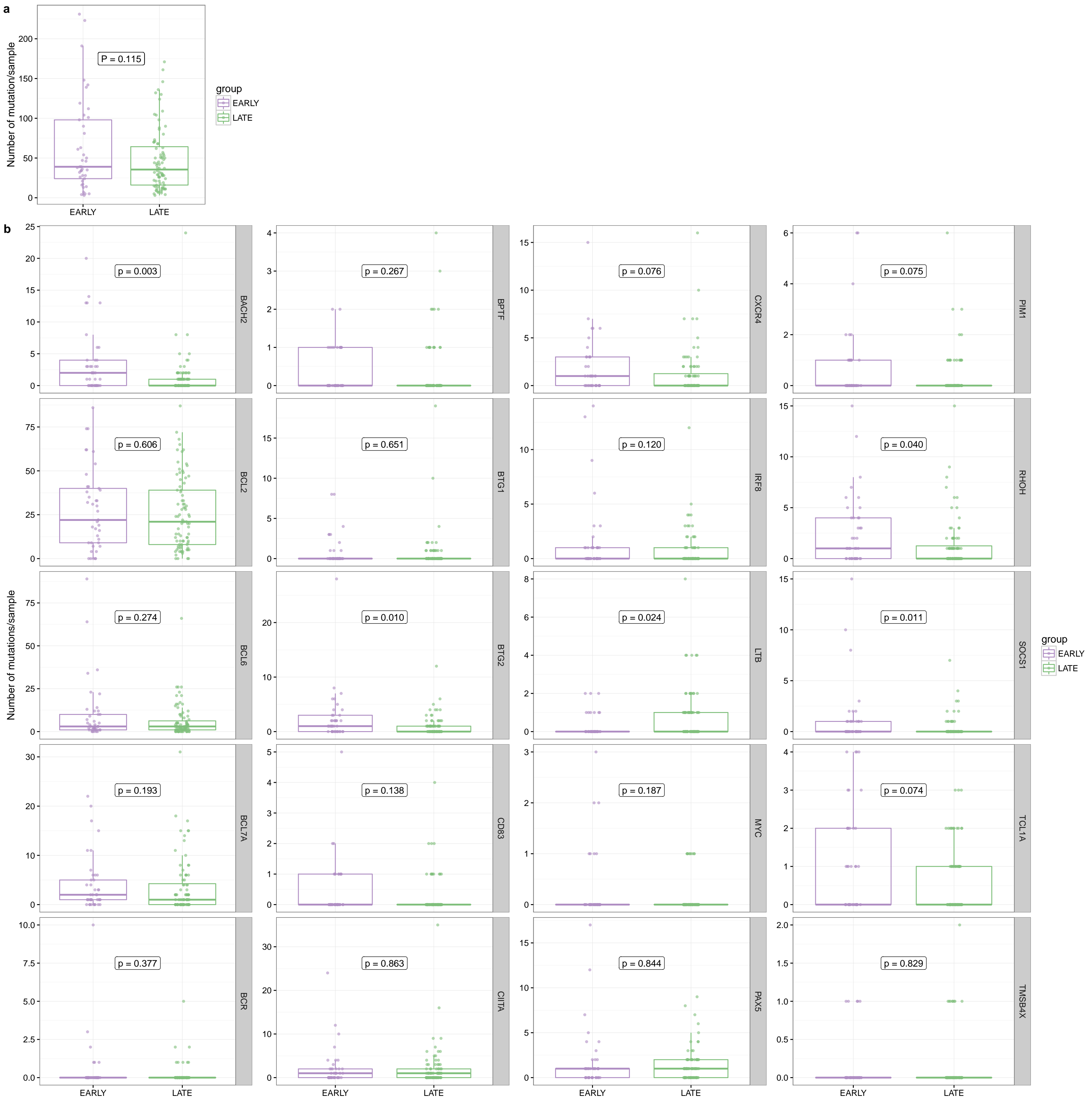

Supplement: S12 Fig — (A) Number of mutations per sample found in 20 genes, by outcome category (41 patients with early progression, 84 patients with late/never progression). (B) Number of mutations per sample, by gene and by outcome category. (PDF) [file pmed.1002197.s013.pdf]

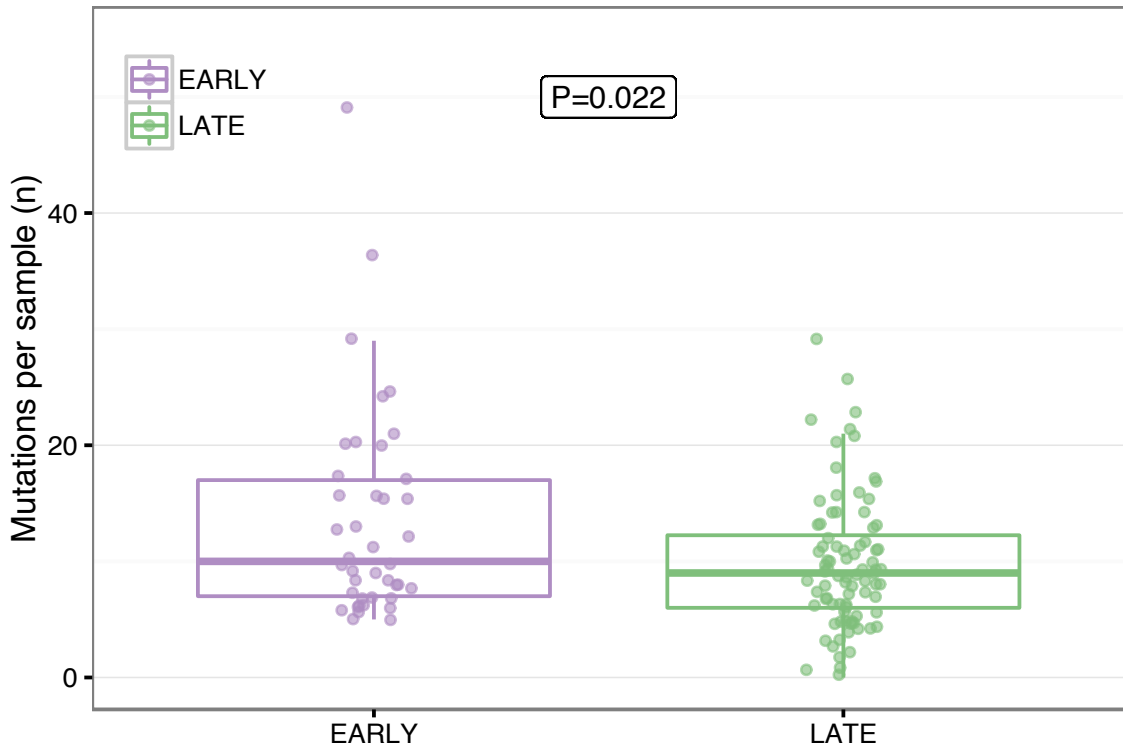

Supplement: S13 Fig — Non-synonymous single nucleotide variants as well as small insertions or deletions were considered in this analysis. (PDF) [file pmed.1002197.s014.pdf]

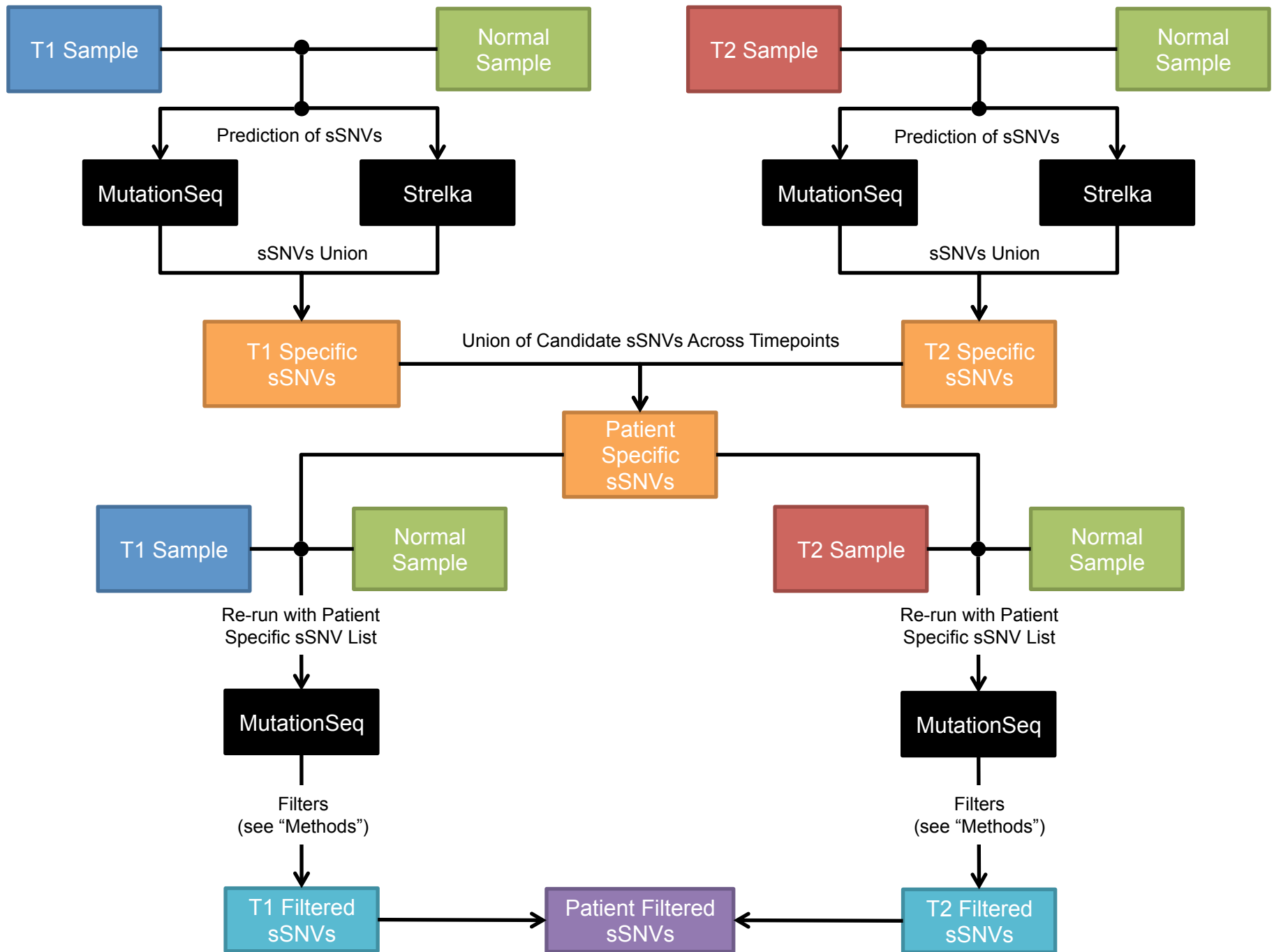

Supplement: S14 Fig — (PDF) [file pmed.1002197.s015.pdf]

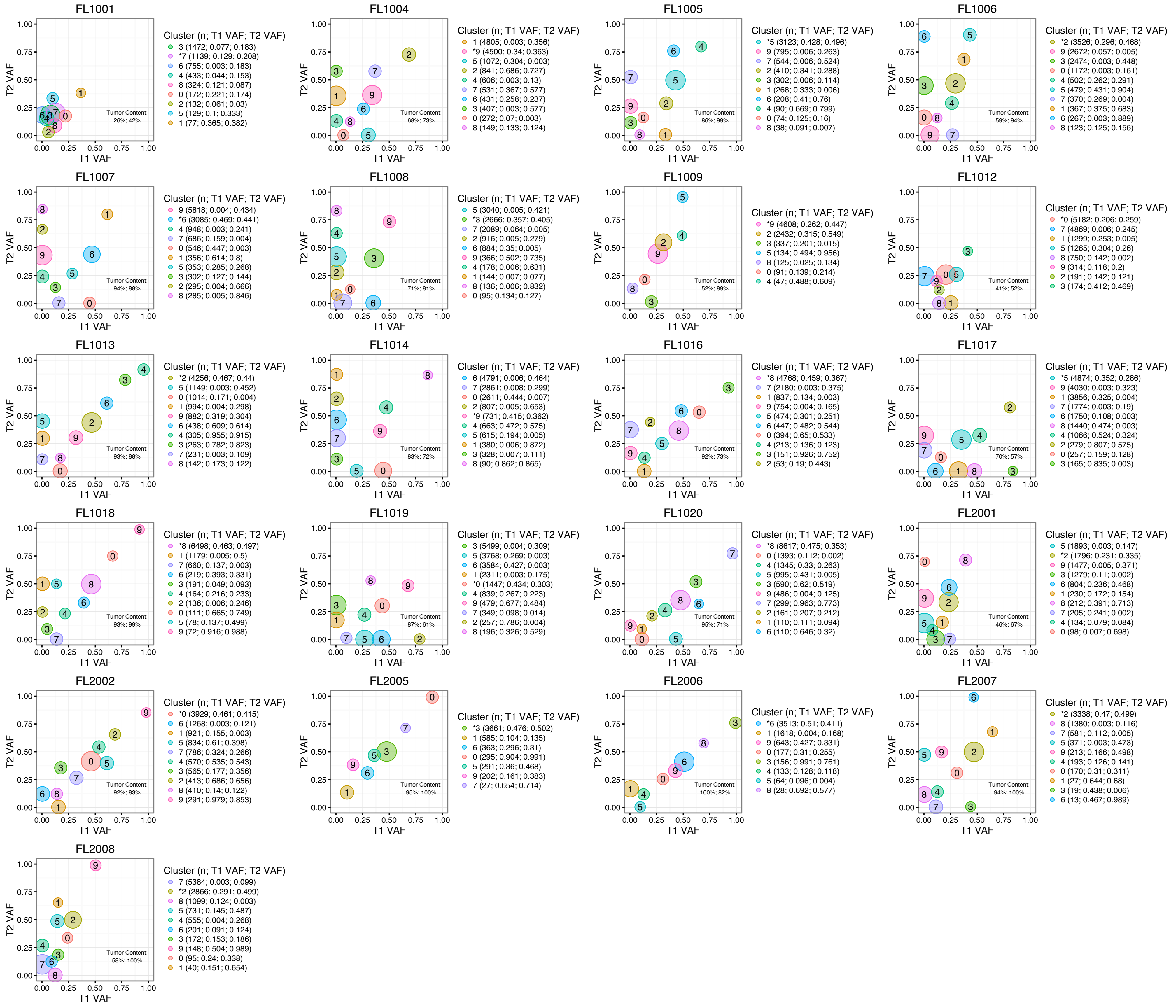

Supplement: S15 Fig — A scatterplot of the mean T2 versus T1 variant allele frequency of each cluster (identified from variational Bayesian binomial mixture model [VBBMM] clustering) in each TFL and PFL patient, with the size of the cluster representing the number of sSNVs in the cluster. The cluster most representative of clonally dominant diploid heterozygous sSNVs in each patient is indicated by an asterisk in the patient legend. Tumor content is calculated by multiplying the mean variant allele frequency of this cluster by two in each time point. The resulting predicted tumor content is listed in the bottom right-hand corner of each patient plot (T1; T2). (PDF) [file pmed.1002197.s016.pdf]

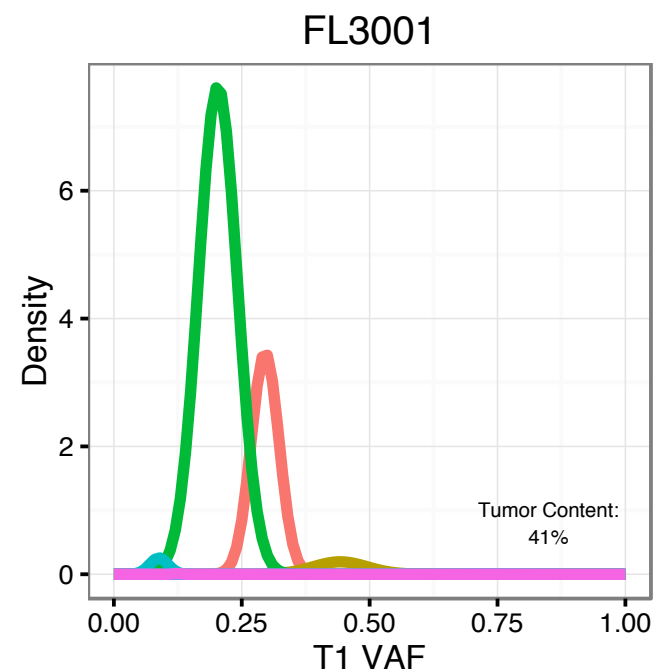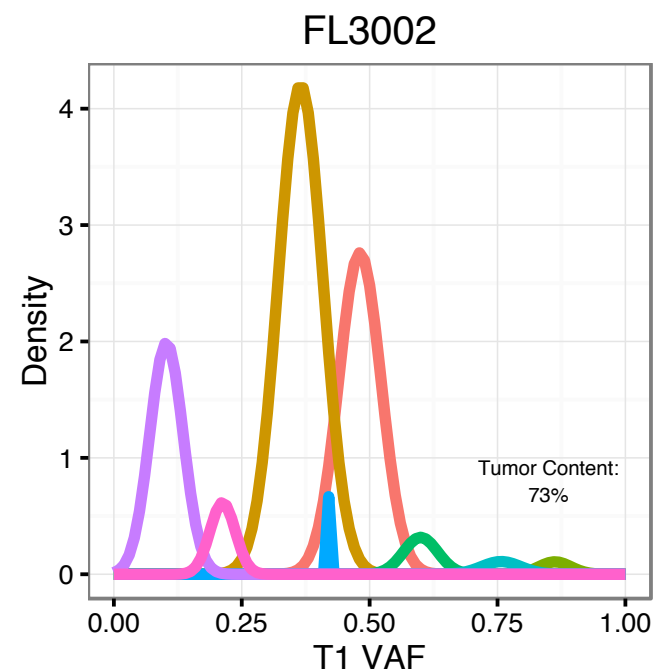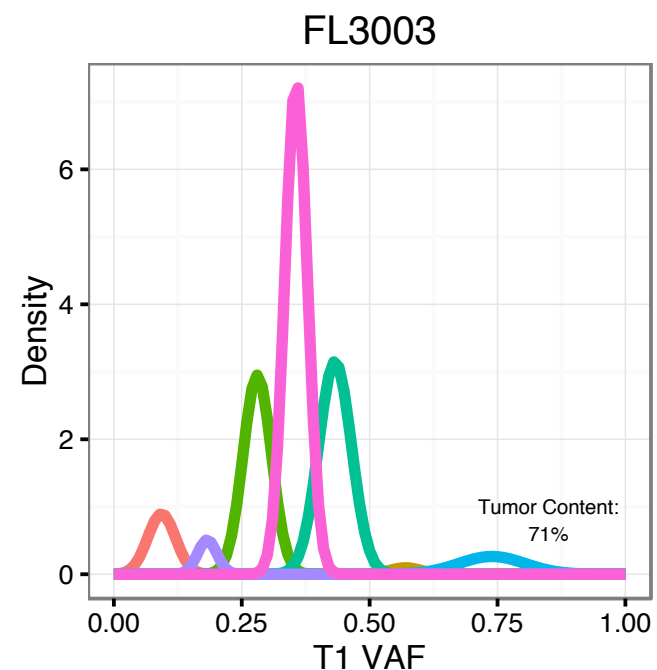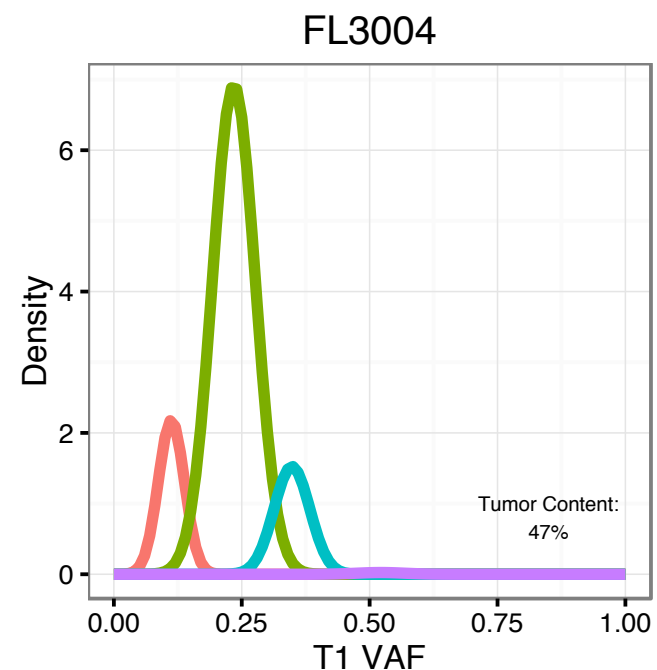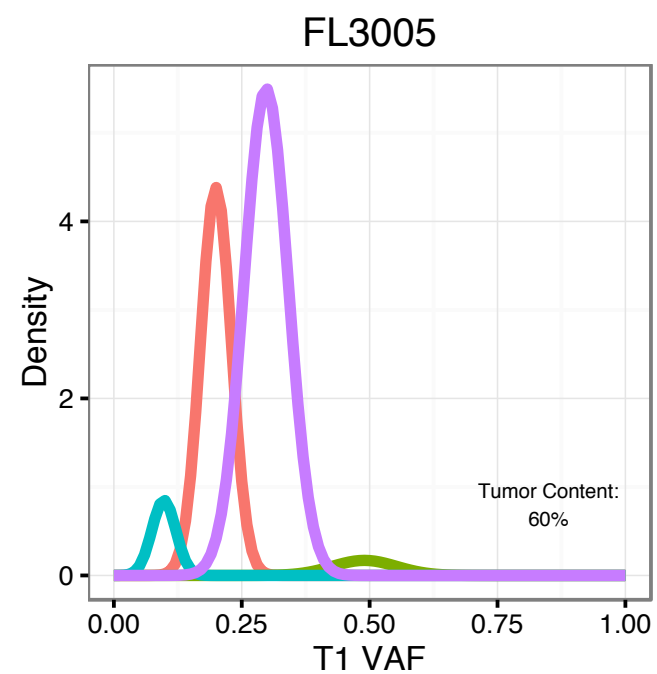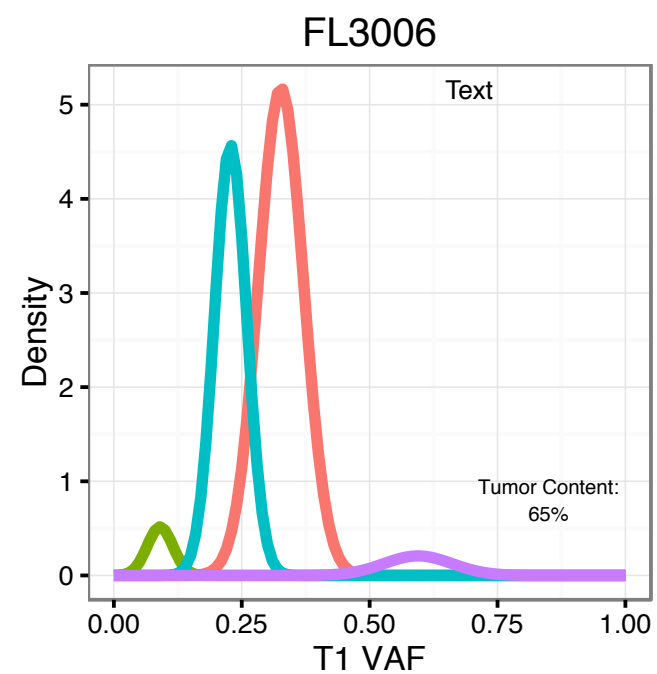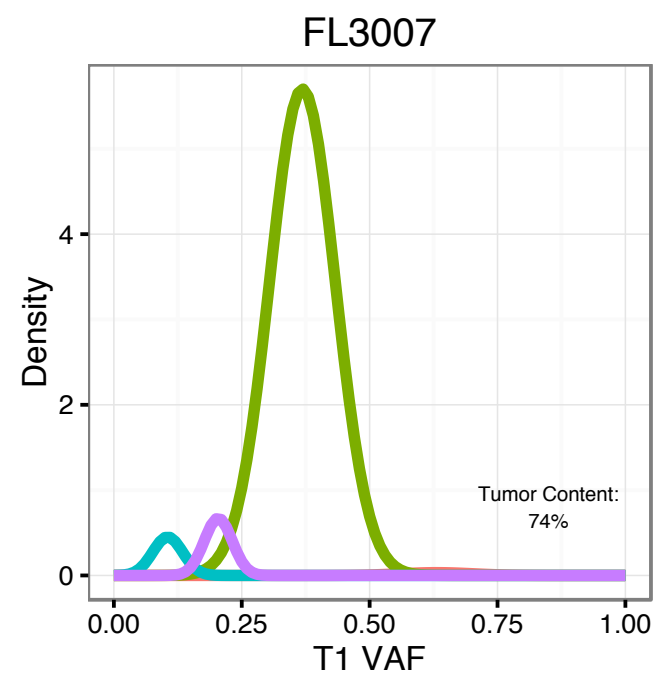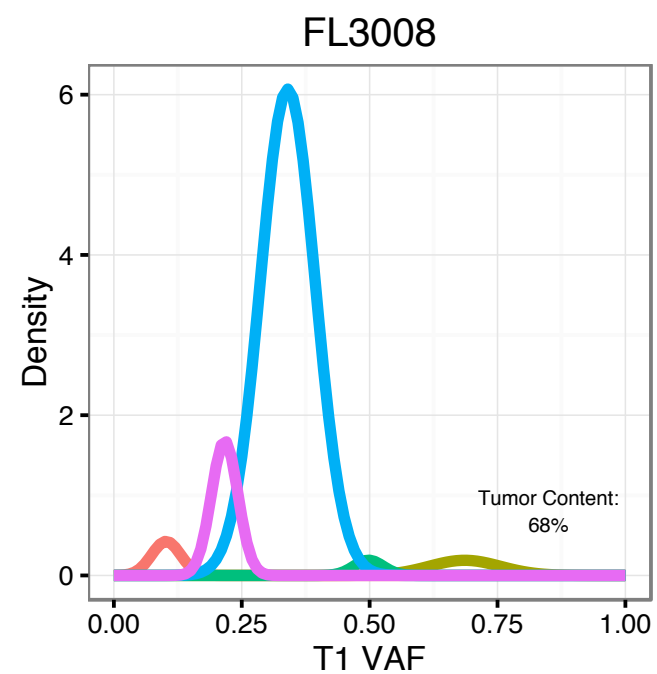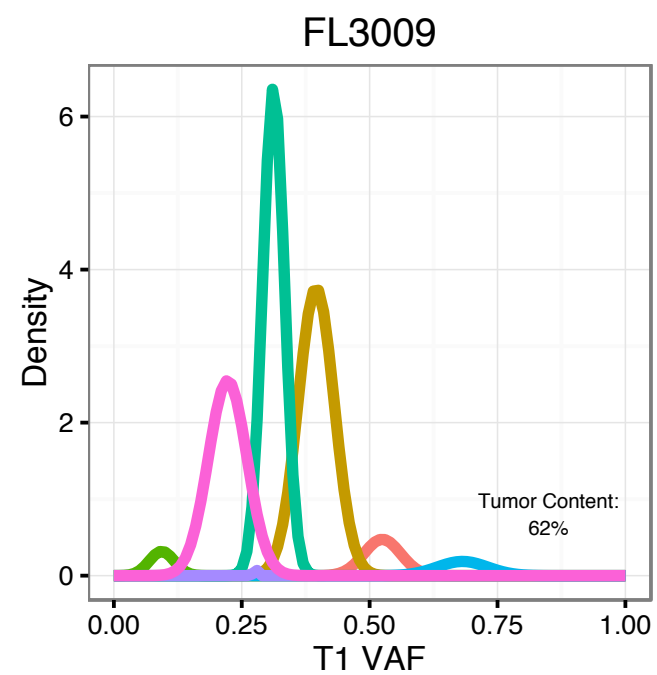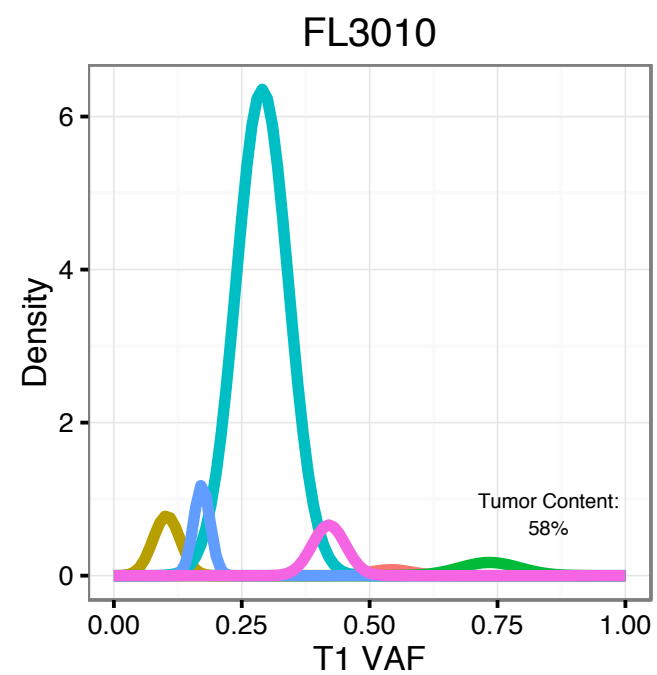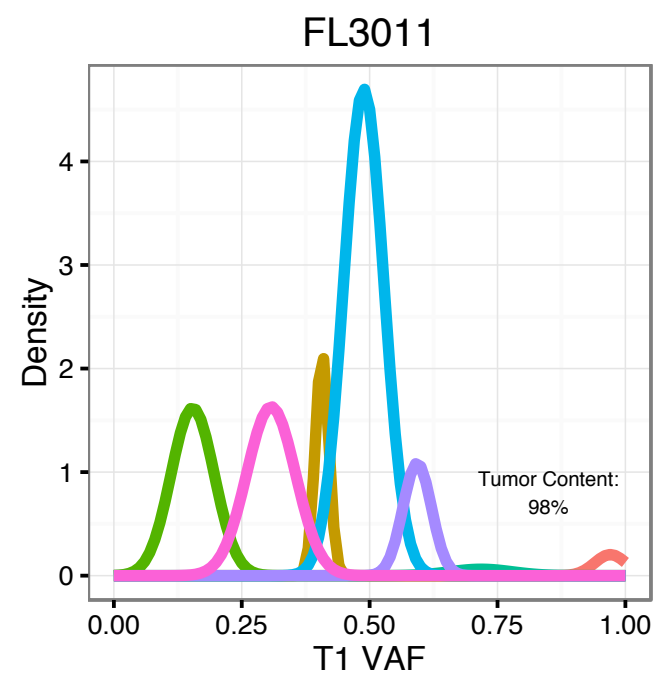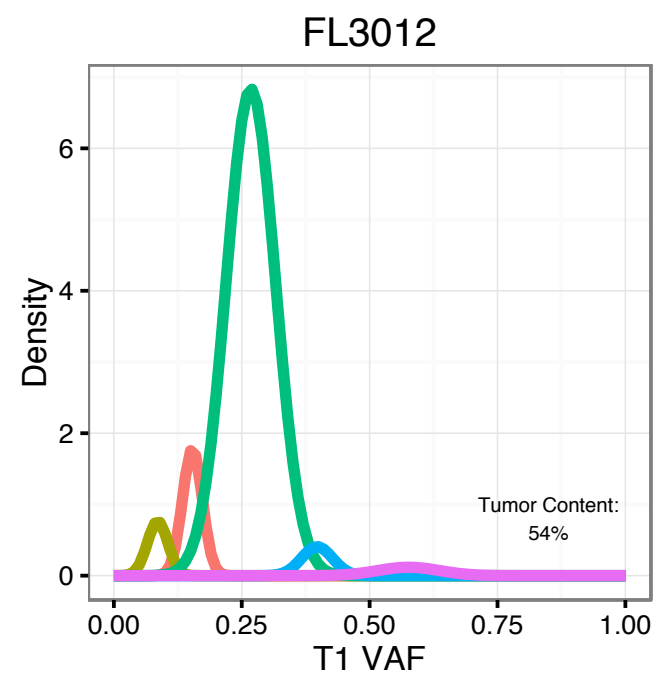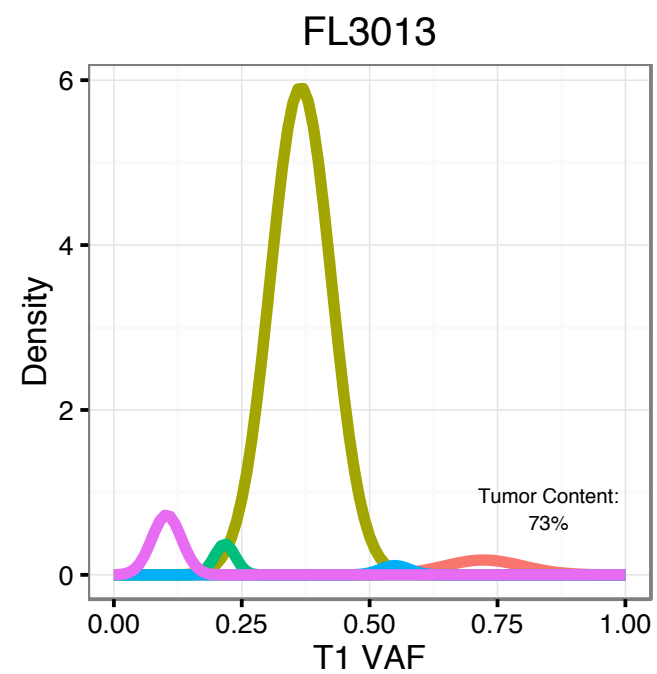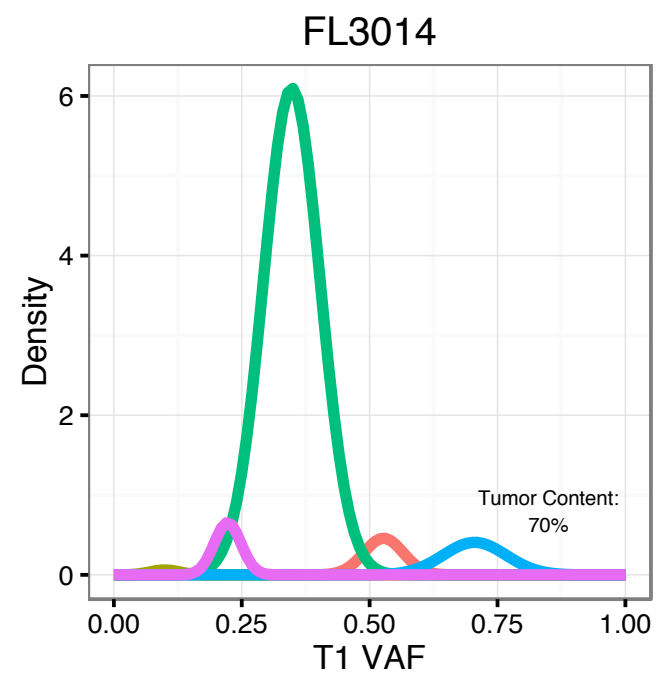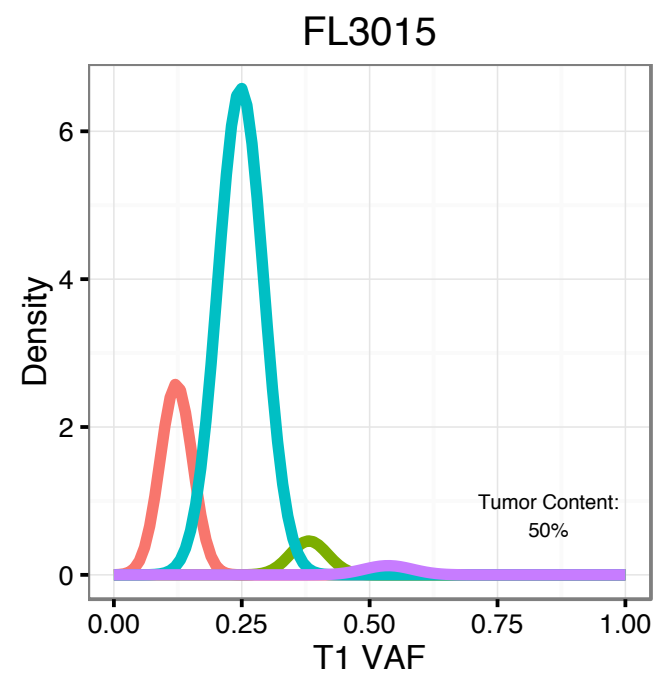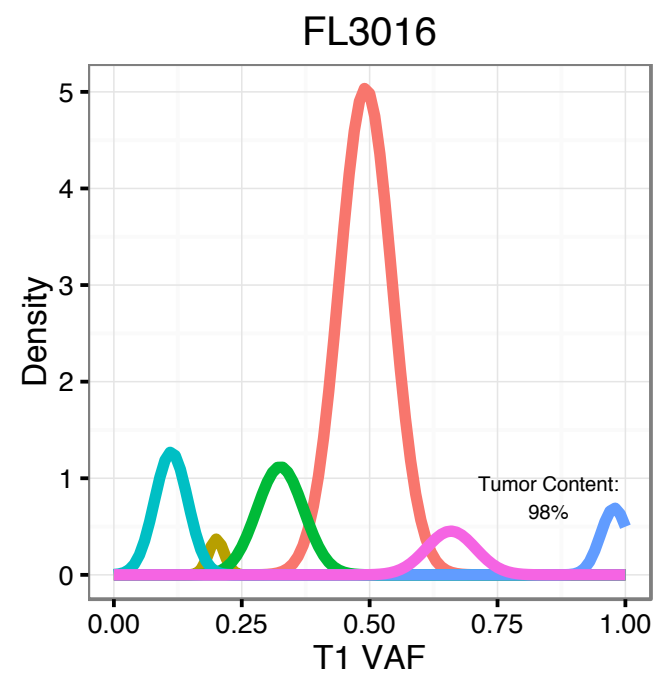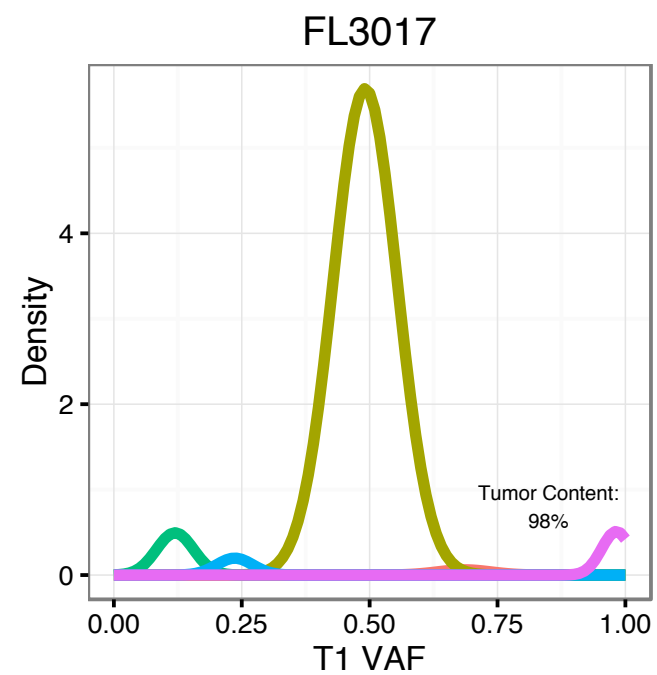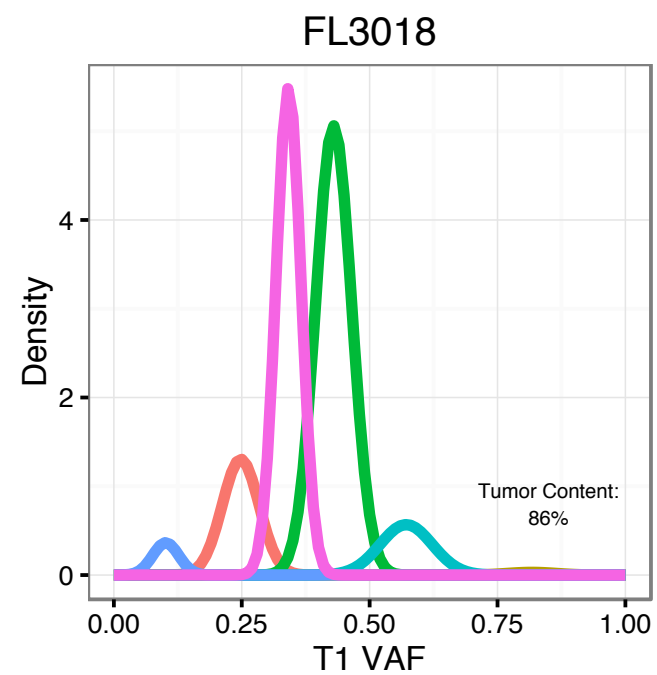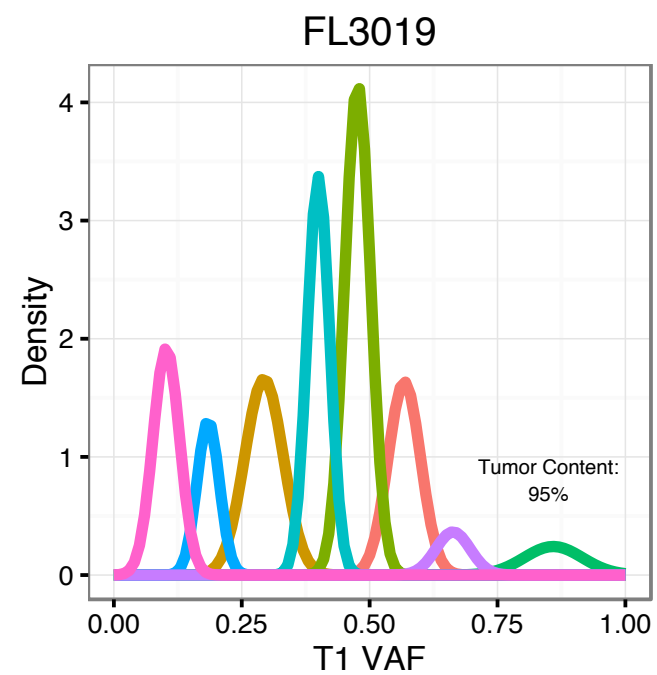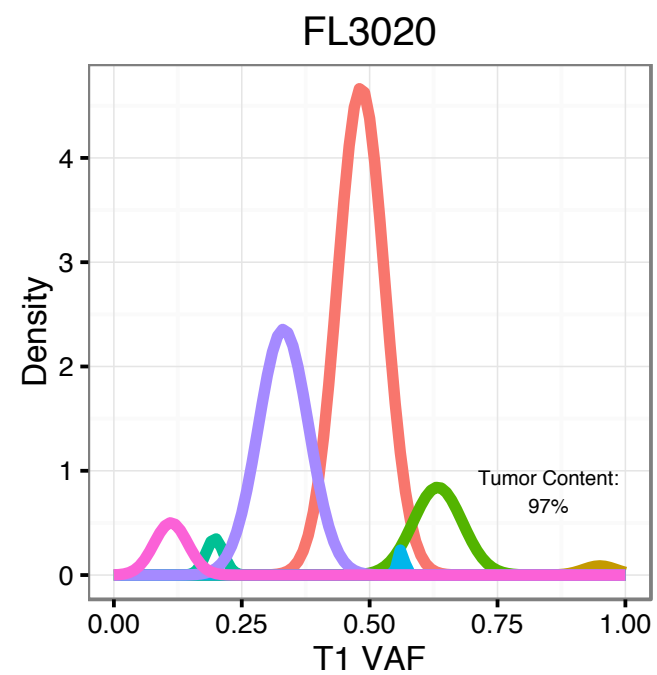

Supplement: S16 Fig — T1 variant allele frequency density plots of each cluster (identified from a VBBMM). The cluster most representative of the diploid heterozygous sSNVs in each patient is indicated by an asterisk in the patient legend. (PDF) [file pmed.1002197.s017.pdf]

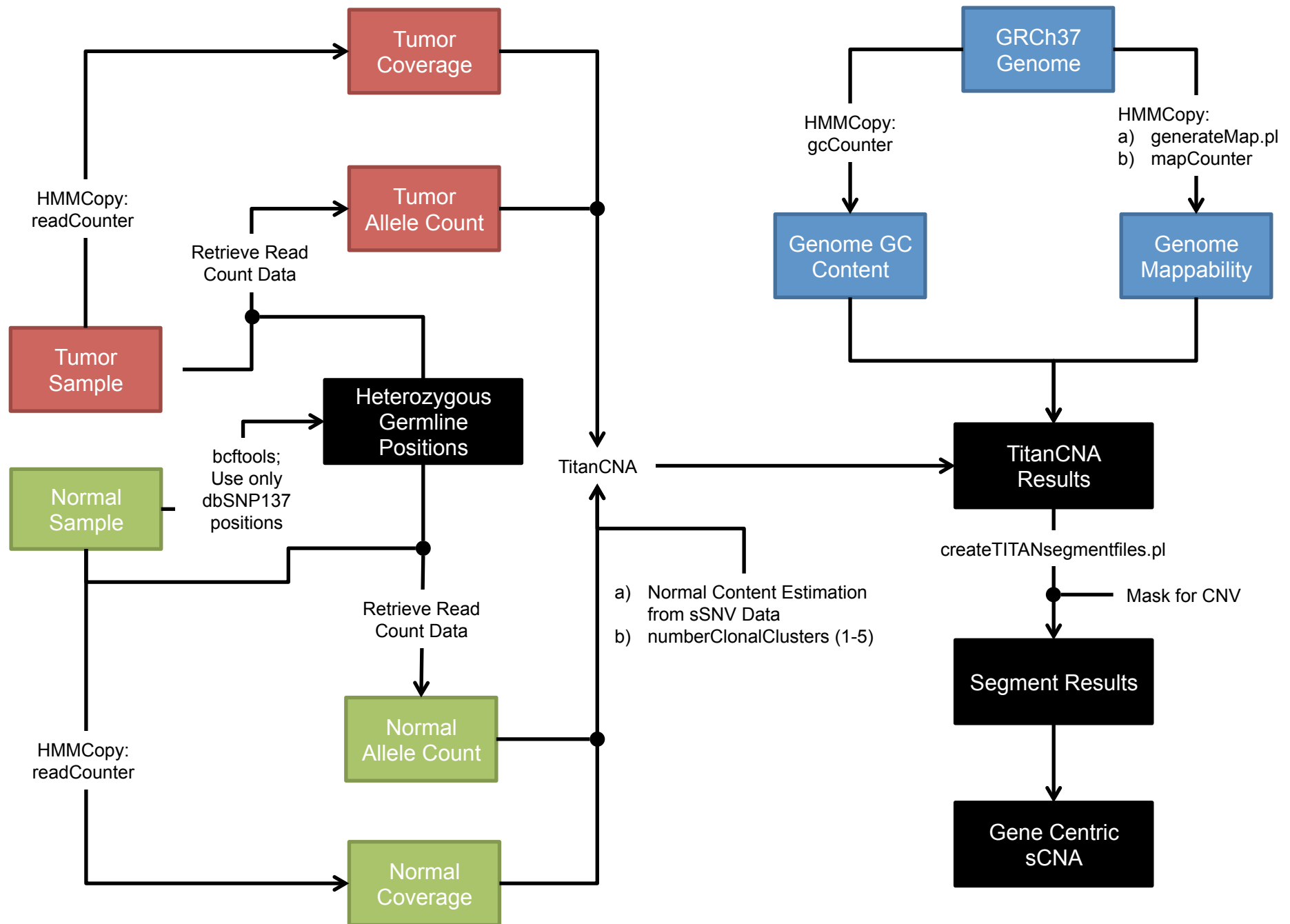

Supplement: S17 Fig — (PDF) [file pmed.1002197.s018.pdf]

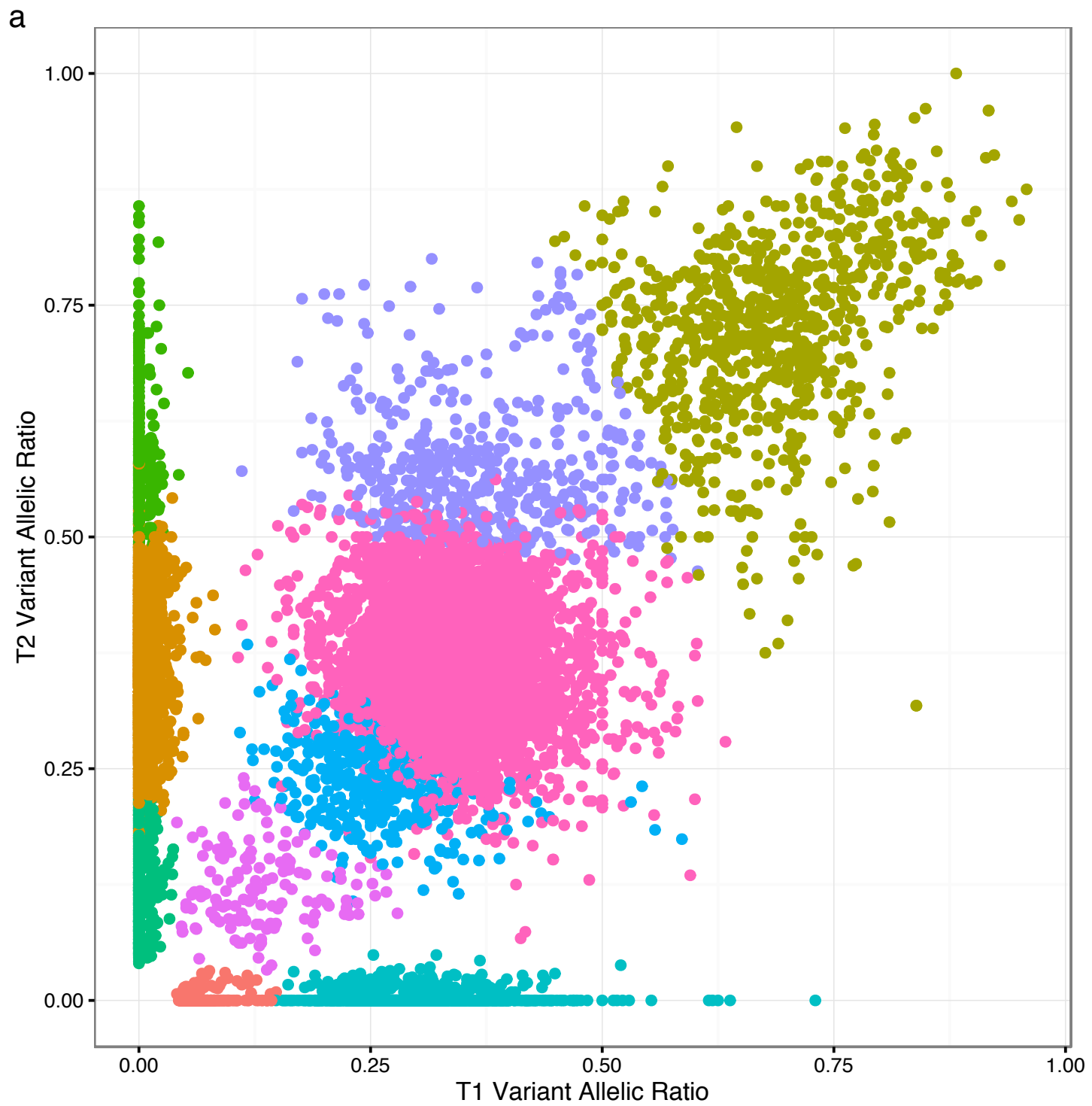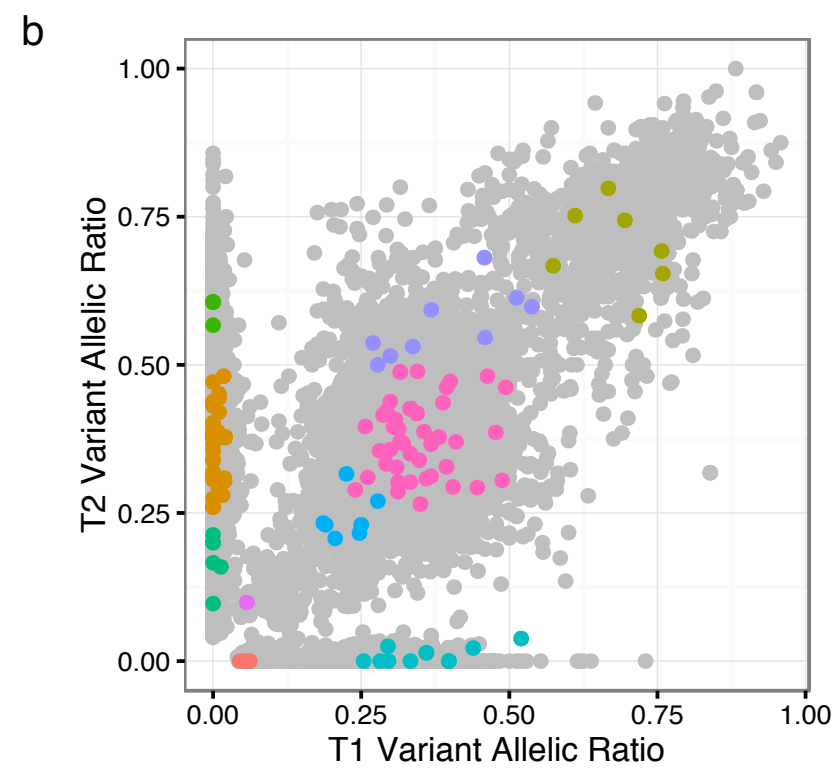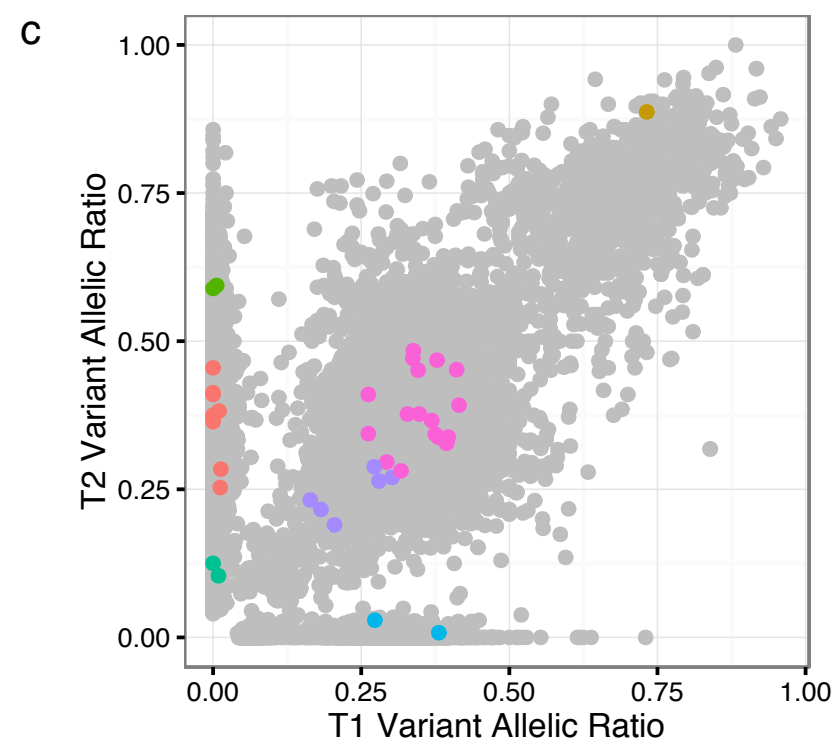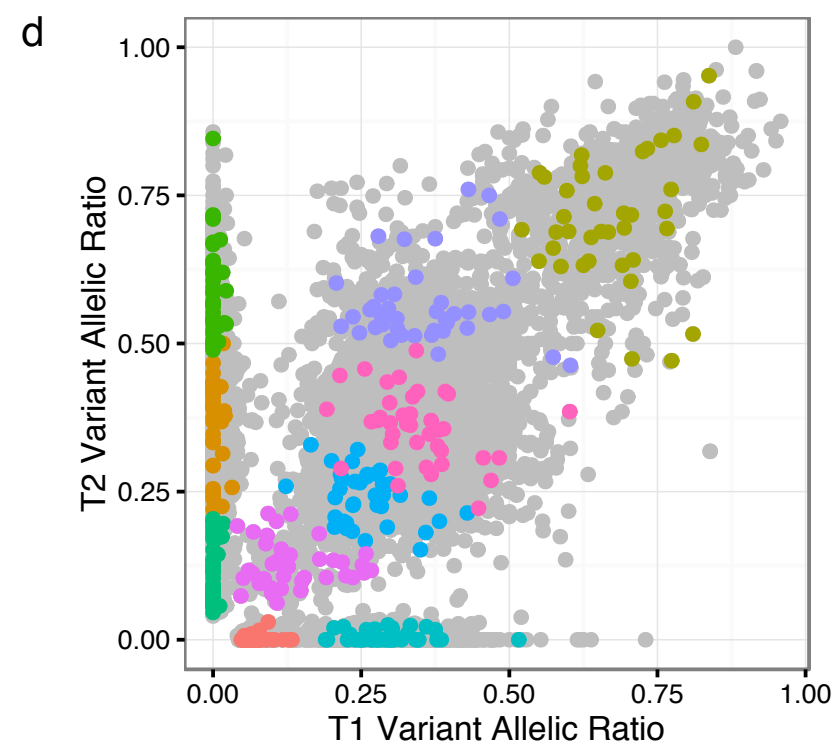

Supplement: S18 Fig — At least 192 positions were selected for deep sequencing validation. This selection included all coding sIndels and non-synonymous coding sSNVs (B), as well as synonymous coding sSNVs (C). To backfill positions to meet the 192-position requirement, we then proportionally sampled non-coding sSNVs from the different clusters (D) identified by VBBMM (A) of the T1 and T2 variant allele frequencies. (PDF) [file pmed.1002197.s019.pdf]

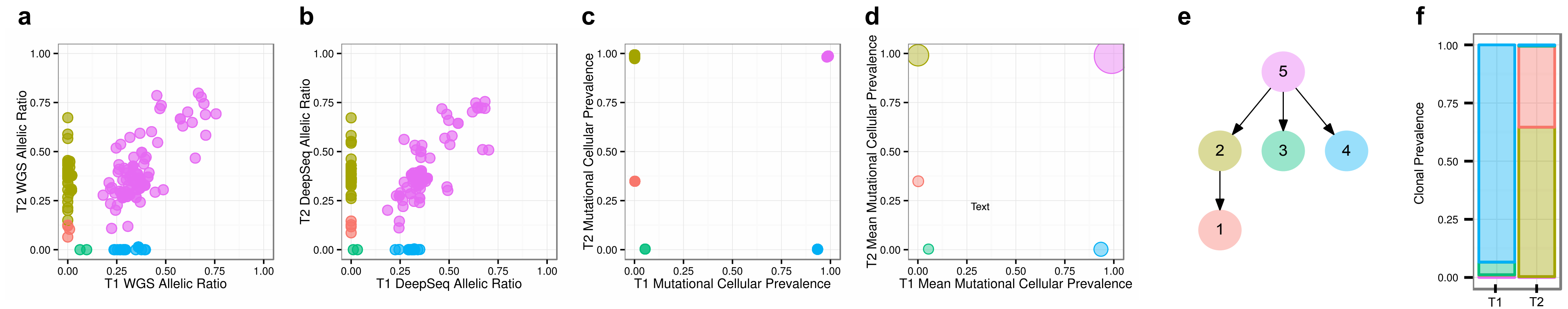

Supplement: S19 Fig — sSNVs, predicted from the T1 (x-axis) and/or T2 (y-axis) samples from whole genome sequencing (A), were selected for targeted deep sequencing validation (B). Validated positions were used as input into PyClone to determine the mutational cellular prevalence of each sSNV (C). sSNVs with similar mutational cellular prevalences were clustered with PyClone, with each cluster’s cellular prevalence being represented by the mean cellular prevalence of all sSNVs in the cluster (D). These cluster cellular prevalences were used as input into Citup to construct clonal phylogenies (E) and clonal prevalences for the T1 and T2 samples (F). sSNVs in each node were propagated down to their children nodes. (PDF) [file pmed.1002197.s020.pdf]

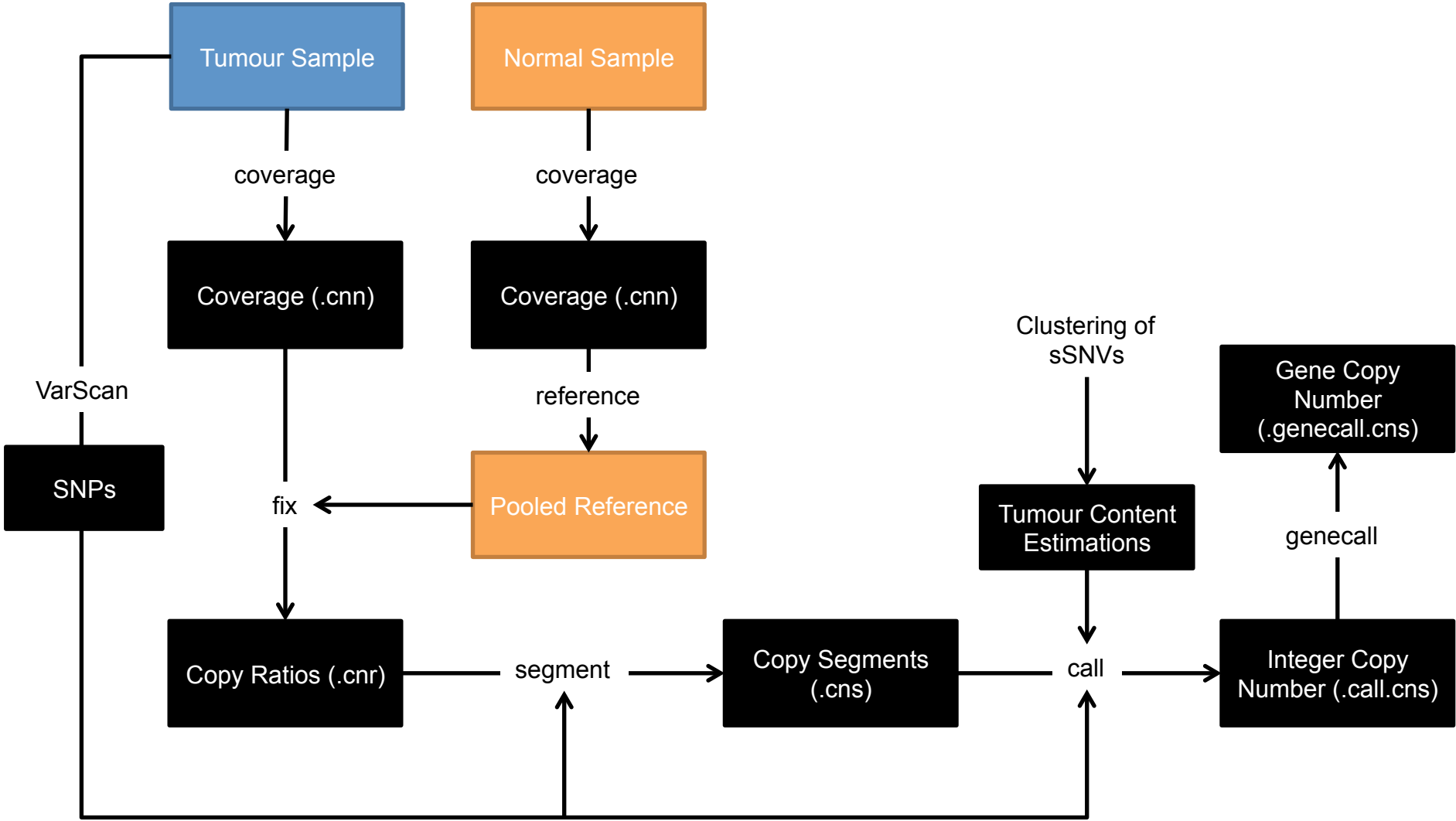

Supplement: S20 Fig — (PDF) [file pmed.1002197.s021.pdf]
