# Supplementary material for: Histological Transformation and Progression in Follicular Lymphoma: A Clonal Evolution Study
Source: PLoS Med. 2016 Dec 13;13(12):e1002197. doi: 10.1371/journal.pmed.1002197 (PMC5154502; doi:10.1371/journal.pmed.1002197)
Supplement: S6 Fig — Shown are five mutations (A–E) in four patients (FL1004, FL1012, FL1019, and FL2001) in which PyClone suggested that the expanded T2-dominant mutation clusters were present at near zero prevalence at T1. Background refers to the variant allele frequencies of all possible single nucleotide changes in the vicinity of the mutation of interest (defined as up to 50 base pairs upstream and up to 50 base pairs downstream). The results are confirmed by digital droplet PCR (rightmost plots). Color coding in the digital droplet PCR plots is as follows: grey = empty droplets; blue = single-positive droplets for wild-type allele; purple = double-positive droplets; red = single-positive droplets for mutant allele. (PDF) [file pmed.1002197.s007.pdf]

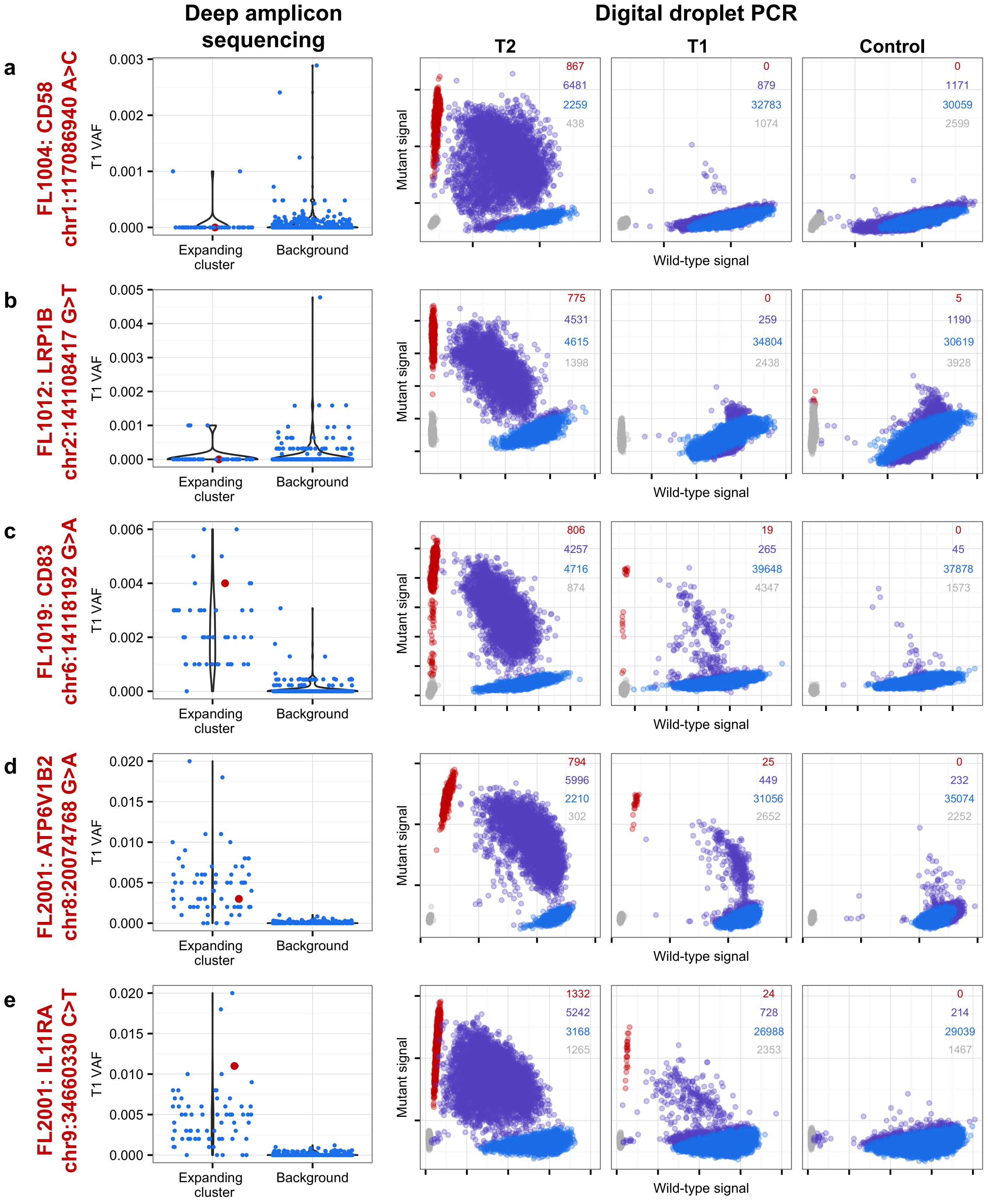

Deep amplicon sequencing
Digital droplet PCR
T2
T1
Control
a
FL1004: CD58
chr1:117086940 A>C
T1 VAF
Expanding cluster
Background
Mutant signal
Wild-type signal
867
6481
2259
438
0
879
32783
1074
0
1171
30059
2599
b
FL1012: LRP1B
chr2:141108417 G>T
775
4531
4615
1398
0
259
34804
2438
5
1190
30619
3928
c
FL1019: CD83
chr6:14118192 G>A
806
4257
4716
874
19
265
39648
4347
0
45
37878
1573
d
FL2001: ATP6V1B2
chr8:20074768 G>A
794
5996
2210
302
25
449
31056
2652
0
232
35074
2252
e
FL2001: IL11RA
chr9:34660330 C>T
1332
5242
3168
1265
24
728
26988
2353
0
214
29039
1467
